# Multi-Sensor Fusion SLAM Based on LiDAR, IMU and GPS for Structured Urban Scenes

**DOI:** 10.3390/jimaging12080345

**Published:** 2026-07-30

**Authors:** Jiajia Lu, Yue Shen, Xu Wang, Fuyang Ke

**Affiliations:** 1 School of Internet of Things Engineering, Wuxi University, Wuxi 214105, China; lujiajia@cwxu.edu.cn; 2 School of Computer Science, School of Software, Nanjing University of Information Science and Technology, Nanjing 210044, China; 3 School of Automation, Nanjing University of Information Science and Technology, Nanjing 210044, China; shenyue0900@163.com (Y.S.); 15306189671@163.com (X.W.); 4 Jiangsu Engineering Research Center of Hyperconvergence Application and Security of loT Devices, Wuxi 214000, China

**Keywords:** multi-sensor fusion, point cloud feature extraction, localization, map building, loop closure detection

## Abstract

Aiming at the current SLAM (Simultaneous Localization and Mapping) algorithms in urban scenarios, which have problems such as elevation drift, odometry drift, and the appearance of false loop closures, a tightly coupled SLAM method with LiDAR and inertial guidance is proposed. In the front-end, a raster-based point cloud feature extraction method is introduced, enabling simultaneous segmentation and extraction of line, surface, and ground features. Utilizing the alignment results of line and surface features as the initial value for ground point alignment, interpolation weights are determined based on roll and pitch angle errors, effectively reducing global elevation errors through frame-by-frame constraints. The back-end employs an error state-based Kalman filter (ESKF) for GPS and IMU data fusion, enhancing the validity of true state estimation. A Scan Context loop closure detection method is designed, augmented by GPS detection as an auxiliary loop closure constraint to mitigate false loop closures. A global factor graph optimization model is also proposed. Experimental results demonstrate that, compared to existing open-source algorithms, the proposed method exhibits improved performance in structured urban scenes, reducing the average RMSE APE by 47.4% compared with LiDAR-only methods and by 22.9% compared with tightly coupled LiDAR-inertial methods. This work highlights the potential of multi-sensor fusion SLAM for achieving high-precision 3D localization and mapping in complex urban environments.

## 1. Introduction

Simultaneous Localization and Mapping (SLAM) has become one of the fundamental technologies enabling autonomous driving, intelligent mobile robots, and unmanned systems to perceive unknown environments and perform autonomous navigation [1,2,3]. Among various SLAM paradigms, LiDAR-based SLAM has attracted extensive attention because LiDAR provides accurate three-dimensional geometric measurements that are robust to illumination variations and environmental appearance changes. Nevertheless, LiDAR-only systems inevitably suffer from accumulated drift during long-distance navigation and performance degradation in feature-poor or highly repetitive environments [4]. Although inertial measurement units (IMUs) provide high-frequency motion observations that effectively compensate for the low acquisition rate of LiDAR, inertial integration inevitably accumulates drift over time [5,6]. GPS provides globally referenced position measurements under favorable satellite visibility, yet its performance deteriorates significantly in urban canyons, tunnels, dense vegetation, and other GNSS-denied environments [7]. Consequently, no individual sensing modality can provide accurate and reliable localization under all operating conditions. Integrating LiDAR, IMU, and GPS measurements has therefore become one of the mainstream solutions for achieving accurate, robust, and long-term localization in autonomous systems [8,9].

In recent years, considerable progress has been achieved in LiDAR-based SLAM. LOAM [4] pioneered a highly influential feature-based odometry and mapping framework by extracting edge and planar features from LiDAR scans, while LeGO-LOAM [10] further exploited ground information to improve localization efficiency on uneven terrain. Other representative systems, including Cartographer [11], HDL-Graph SLAM [12], MULLS [13], and surfel-based SLAM [14], demonstrated the effectiveness of graph optimization, geometric registration, and multi-metric constraints for improving mapping consistency. In addition, point cloud registration techniques such as ICP [15], Generalized ICP (GICP) [16], and Normal Distribution Transform (NDT) [17] have laid the theoretical foundation for accurate scan matching and remain widely adopted in modern LiDAR SLAM systems.

To further improve localization robustness, recent studies have increasingly focused on tightly integrating LiDAR and inertial measurements. IMU preintegration on manifolds proposed by Forster et al. [6] provides an efficient mathematical framework for high-frequency inertial fusion and has become the basis of many modern LiDAR–IMU systems. Representative approaches, including LIO-SAM [18], FAST-LIO [19], FAST-LIO2 [20], and Point-LIO [21], have significantly improved localization accuracy, computational efficiency, and robustness through tightly coupled LiDAR–IMU estimation based on factor graph optimization [22] or iterated Kalman filtering. Meanwhile, visual–inertial estimation methods such as VINS-Mono [23] have further demonstrated the effectiveness of tightly integrated multi-sensor fusion for robust state estimation.

Loop closure detection is another key component affecting long-term localization consistency. Scan Context [24] and its improved version Scan Context++ [25] provide efficient global descriptors for LiDAR place recognition and have become widely adopted in LiDAR SLAM systems. More recently, learning-based approaches such as OverlapTransformer [26] improve place recognition robustness through Transformer-based feature representation, while BoW3D [27] achieves efficient real-time loop detection using a three-dimensional bag-of-words model. LinK3D [28] further enhances feature matching robustness by constructing discriminative linear keypoint descriptors for LiDAR point clouds. Although these methods have significantly improved place recognition performance, false loop closures remain challenging in structured urban environments characterized by repetitive buildings, roads, and similar geometric layouts.

Despite these advances, several challenges remain underexplored in practical urban applications. First, conventional feature extraction methods primarily focus on edge and planar features while underutilizing stable ground information, resulting in cumulative elevation drift during long-distance navigation. Second, many existing front-end methods treat computational efficiency and feature quality independently, making it difficult to simultaneously achieve efficient processing and robust geometric constraints. Third, although Scan Context-based loop detection is computationally efficient, repetitive urban scenes may still produce incorrect loop candidates. Finally, GPS observations are commonly introduced only as global position constraints, while their uncertainty is rarely exploited to improve loop verification reliability and overall system robustness.

To address these limitations, this paper proposes a LiDAR-IMU-GPS multi-sensor fusion SLAM framework for structured urban environments. Rather than introducing an entirely new SLAM architecture, this work focuses on improving several key modules within an existing factor-graph framework to enhance localization accuracy, robustness, and computational efficiency. Specifically, a raster-based feature extraction strategy is proposed to simultaneously extract edge, planar, and ground features while reducing computational complexity. A ground-constrained elevation optimization method is designed to suppress cumulative vertical drift by adaptively combining conventional feature registration with ground-point alignment. Furthermore, a dual IMU preintegration strategy is introduced to improve state estimation consistency within the factor-graph optimization framework. Finally, a GPS-assisted Scan Context loop verification strategy is designed by jointly exploiting GPS covariance and spatial consistency to effectively reduce false loop closures in repetitive urban environments.

Existing LiDAR-inertial SLAM systems primarily focus on either front-end feature extraction or back-end optimization independently. While LOAM-series methods achieve efficient feature extraction through curvature-based segmentation, they suffer from information loss in structured urban environments where planar surfaces dominate. Tightly coupled systems such as FAST-LIO2 improve state estimation accuracy through iterated Kalman filtering, yet they lack explicit mechanisms to suppress elevation drift in long-term operation. Meanwhile, GPS-aided SLAM systems typically integrate global positioning through simple factor injection without considering the quality and reliability of GPS measurements. These limitations motivate our work, which aims to develop a principled integration strategy that combines raster-based feature extraction, ground-constrained optimization, dual-time-scale IMU preintegration, and GPS-assisted loop verification within a unified theoretical framework.

The main contributions of this paper are summarized as follows:

1. A raster-based point cloud feature extraction strategy is proposed to improve both computational efficiency and geometric feature quality by simultaneously extracting edge, planar, and ground features.
2. A ground-constrained elevation optimization method is developed to effectively suppress long-term elevation drift through adaptive ground feature registration.
3. A dual IMU preintegration strategy is incorporated into the LiDAR–IMU–GPS factor-graph optimization framework to improve localization consistency and robustness.
4. A GPS-assisted Scan Context loop verification strategy is proposed to reduce false loop closures by integrating GPS covariance information with geometric consistency constraints while maintaining real-time performance.

The remainder of this paper is organized as follows. Section 2 introduces the theoretical foundations of the proposed system. Section 3 presents the proposed front-end feature extraction and multi-sensor fusion framework. Section 4 describes the back-end optimization and GPS-assisted loop closure strategy. Section 5 evaluates the proposed method on both public datasets and self-collected campus datasets. Finally, Section 6 concludes the paper and discusses future work.

## 2. System Framework

**Figure 1:**
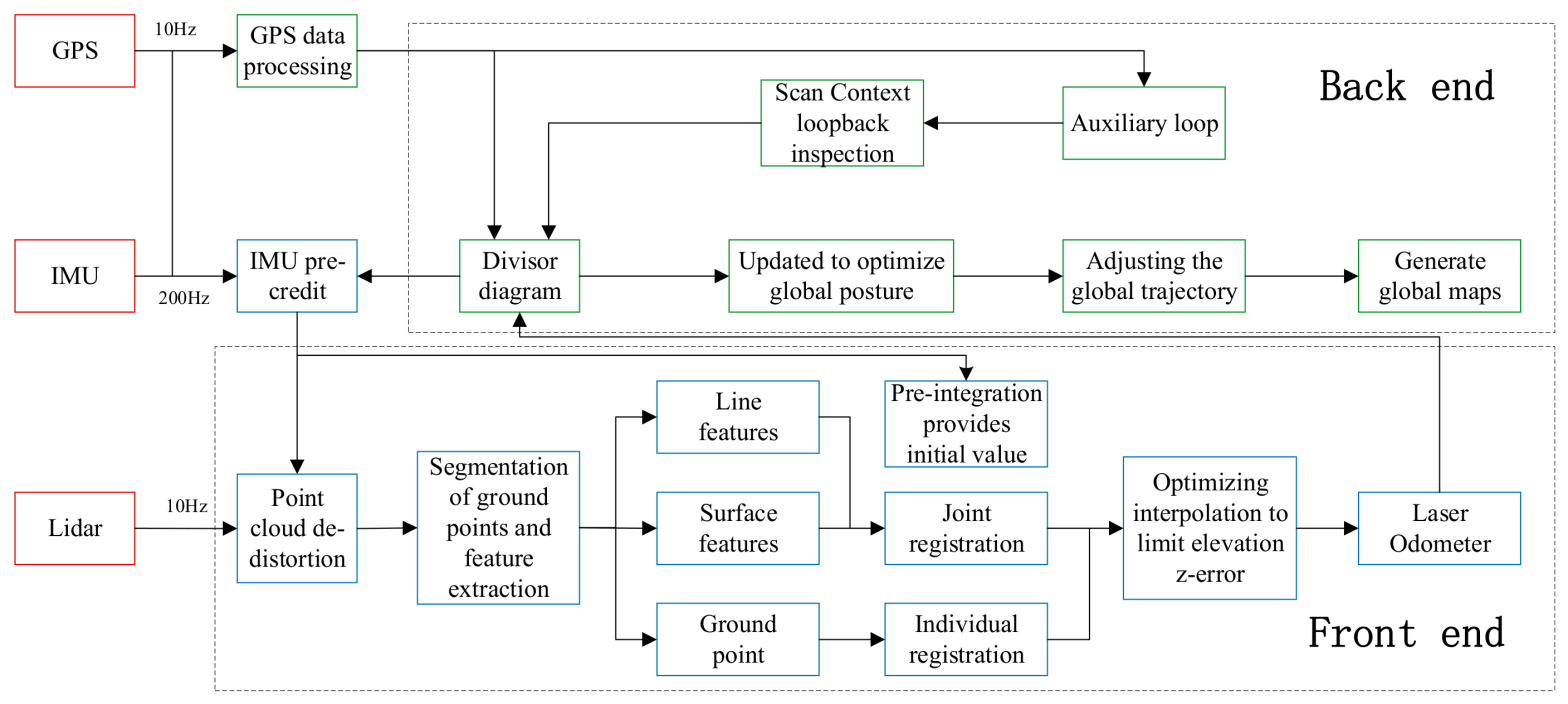
System framework diagram.

The overall framework of the tightly coupled SLAM algorithm based on LiDAR combined inertial guidance designed for structured urban scenes in this paper is shown in Figure 1.

The blue part of the figure shows the front-end design of the SLAM algorithm, which proposes a fast raster-based point cloud feature extraction method, extracting line features, surface features and ground points at the same time; the extracted ground points are individually aligned by using Scan-to-Map’s surface feature solving method; the results obtained by the joint optimization of the line features and surface features are used as the initial value of the ground point alignment; the weighted interpolation is carried out for the results obtained by combining. The results of joint alignment are weighted interpolation; the IMU ppreintegrationmodule is designed, and the results of IMU pre-integration are used for the point cloud to remove motion aberrations and the initial value of point cloud alignment, so as to obtain a more accurate front-end laser odometer.

The green section illustrates the back-end design of the SLAM algorithm, which adopts the ESKF method to fuse the GPS and IMU data and adds the fused results to the factor map for optimization; designs the back-end loop closure detection and introduces the GPS detection as an auxiliary loop closure constraint to determine whether there is a false loop closure or not; and designs the global factor map optimization model to optimize the global pose and adjust the global trajectory to obtain a more consistent global map.

## 3. Tightly Coupled Front-End Odometers

### 3.1. Raster-Based Point Cloud Feature Extraction

Point cloud feature extraction is an important part of point cloud data preprocessing, including line feature and surface feature extraction for a frame of point cloud. In this paper, a frame of point cloud is divided into several subsets equally, and each subset is processed as follows: select a point $p_{i}$ in the point cloud $P_{t}$ at time t. Find 5 points in the same vertical direction of point $p_{i}$, left and right, and construct a set S. The smoothing for each point is calculated as

(1) $$c=\frac{1}{\left|S\right|*\left\|r_{i}\right\|}\left\|\sum_{j\in S,j\neq i}\left(r_{j}-r_{i}\right)\right\|$$

where $r_{i}$ is the distance from the point $p_{i}$ to the origin selected from the point cloud set $P_{t}$, and S is the point cloud set consisting of 5 points before and after the neighboring ones. In order to make the distribution of feature points more uniform, the smoothness of each subset is sorted, taking the 16-line LiDAR used in this paper as an example, there are up to 1800 points in each scanning line, which is divided into six subsets of 300 points per segment, and the points with curvature greater than the threshold value of the corner point are classified as the corner point, and 20 of them are selected in each subset, and the rest of the points are added into the planar point set as the optimization of planar points.

However, when confronted with structured urban scenes, the ground points are a small percentage of a frame of the point cloud, and the elevation will be degraded. With the increasing cumulative error in elevation, it leads to the problem of elevation drift in the construction of the map, and at the same time, the front-end is a process with high real-time requirements, which needs to complete the feature extraction in as short a time as possible and consume fewer resources. In order to solve the above problems, this paper aiming at the characteristics of structured urban scenes, where the ground is generally flat and objects such as buildings, street lamps, and trees are approximately perpendicular to the ground, proposes a raster-based point cloud feature extraction method, which is able to realize the work of extracting the line features, surface features, and ground points at the same time. The core idea of the algorithm design is to divide a frame of point cloud into sectors, utilize structured scene feature points to reduce the amount of computation, and make full use of LiDAR’s own line number information and multi-thread acceleration.

**Figure 2:**
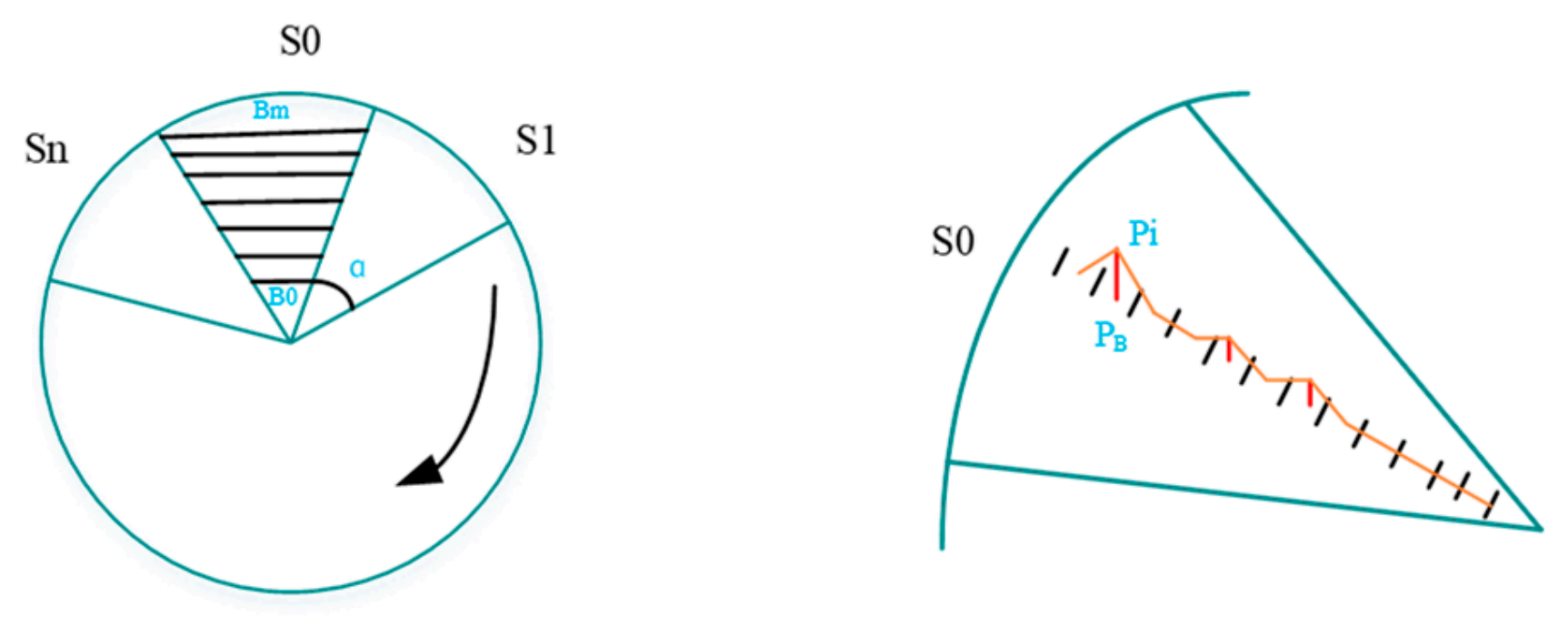
Schematic diagram of point cloud sector segmentation.

Firstly, the single-frame point cloud is preprocessed, and the straight-pass filter is used to filter out the noise points that are obviously below the ground, which mainly come from the anomalies of specular reflection and puddle reflection, and the filter expression is

(2) $$P_{t}\in \left\{p_{i.z}>-(h+2\sigma +\delta )\right\}$$

where h denotes the sensor mounting height, σ denotes the LiDAR noise error, and δ denotes a redundancy margin adjusted according to the actual scene. After the data preprocessing is completed, a frame of the point cloud is segmented into 360 sectors by azimuth; taking 16-line LiDAR as an example, each sector has 16 regions, and a frame of the radar is segmented into 5760 rasters, and the segmentation is shown in Figure 2, where the rasters are labeled with the height minima in each raster, which is used as the reference point of the current raster.

The point $P_{B}$ in the figure represents the reference point of a raster, categorizing each raster in the direction of increasing sector radius. Traverse each raster reference point in the sector, mark the first raster reference point $P_{A}$ as the starting point, and then calculate the slope of the other consecutive raster reference points $P_{B}$ in the same sector with the starting raster reference point $P_{A}$, and the slope calculation formula is shown in Equations (3)–(5):

(3) $$\begin{array}{c} \delta x=\sqrt{{\left(P_{A,x}\right)}^{2}+{\left(P_{A,y}\right)}^{2}} \end{array}-\sqrt{{\left(P_{B,x}\right)}^{2}+{\left(P_{B,y}\right)}^{2}}$$

(4) $$\delta y=P_{A,z}-P_{B,z}$$

**Figure 3:**
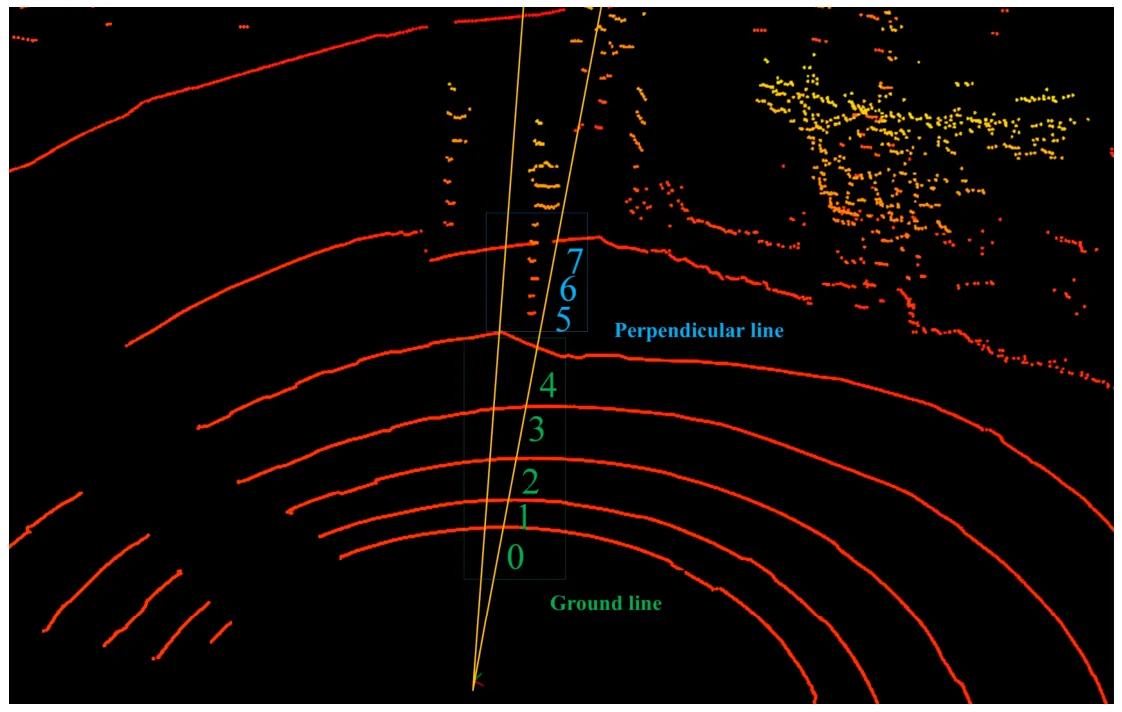
Distribution characteristics of point clouds in structured scenes.

(5) θ=arctan(δx/δy)

where δx denotes the radial distance between the current point and the reference point projected onto the 2D plane, δy denotes the height difference between the two reference points, and θ denotes the angle between the current point $P_{B}$ and the reference point $P_{A}$. As shown in Figure 3, the ground and street lamps in the structured scenario are scanned by LIDAR, with lines 0–4 being the ground line without slope θ small, and lines 5–7 being the street lamp poles perpendicular to the ground θ large. They are classified into the same sector, in the direction of increasing radius, and their belonging to the same class is determined according to the slope threshold θ of the reference point.

**Figure 4:**
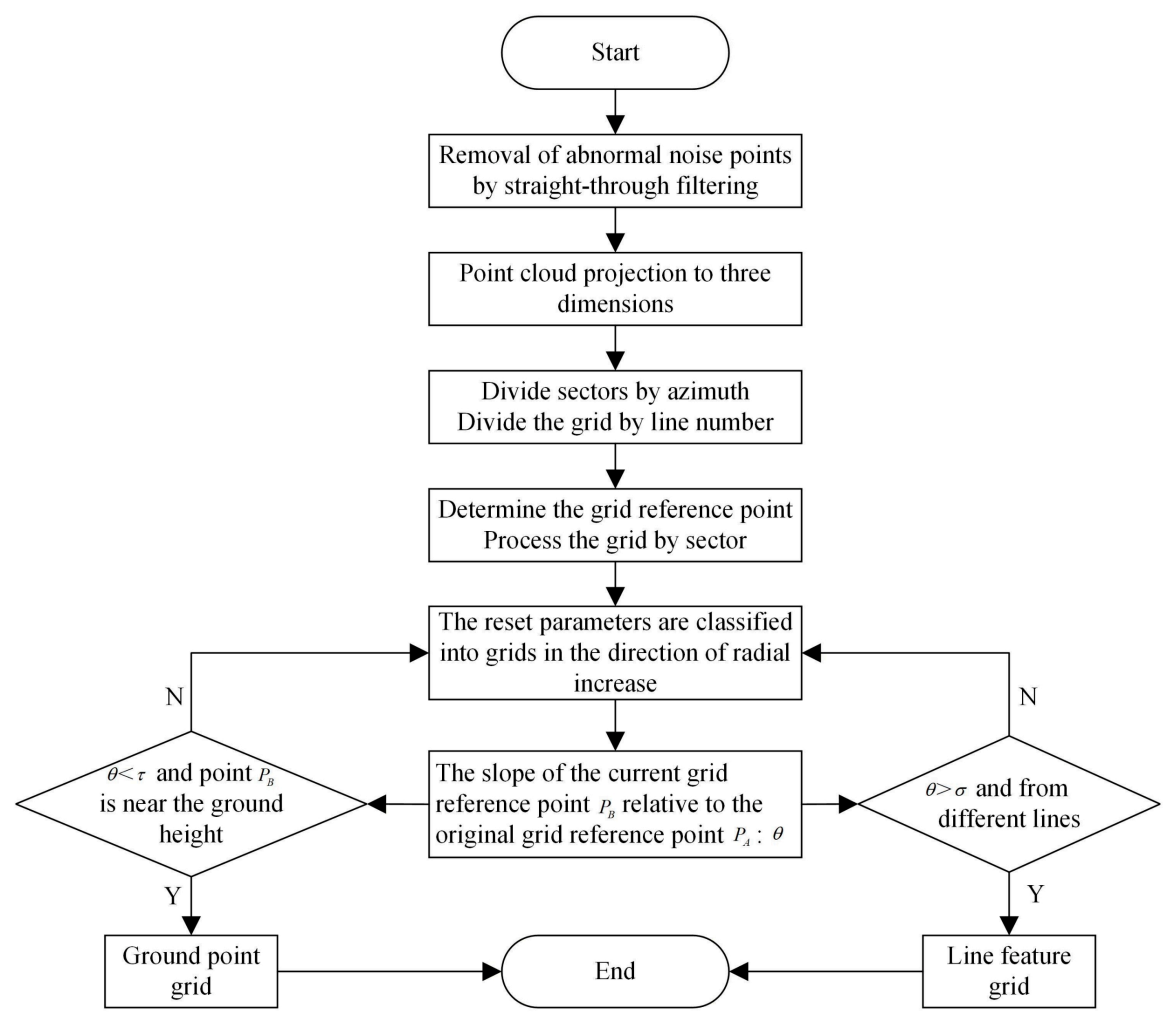
Schematic of rasterized feature extraction.

During the division process, the similar grid slope is greater than a threshold value to end the current category division. Reset the starting point of the new category raster and calculate the slope with the reference point of the new starting point raster as the origin. In Figure 3, line 0 is the starting point of the ground grid, and the slope between lines 4 and 5 is greater than the threshold of the ground grid, ending the current category division, resetting line 5 as the new starting point of the grid, and continuing to calculate the slope division of the same category; the specific process of point cloud segmentation is shown in Figure 4.

Where τ indicates the maximum slope of the ground grid, and μ indicates the maximum slope of the line feature grid, both parameters can be dynamically adjusted according to the segmentation effect, and this paper sets $\tau =6^{\circ }$, $\mu =80^{\circ }$.

**Figure 5:**
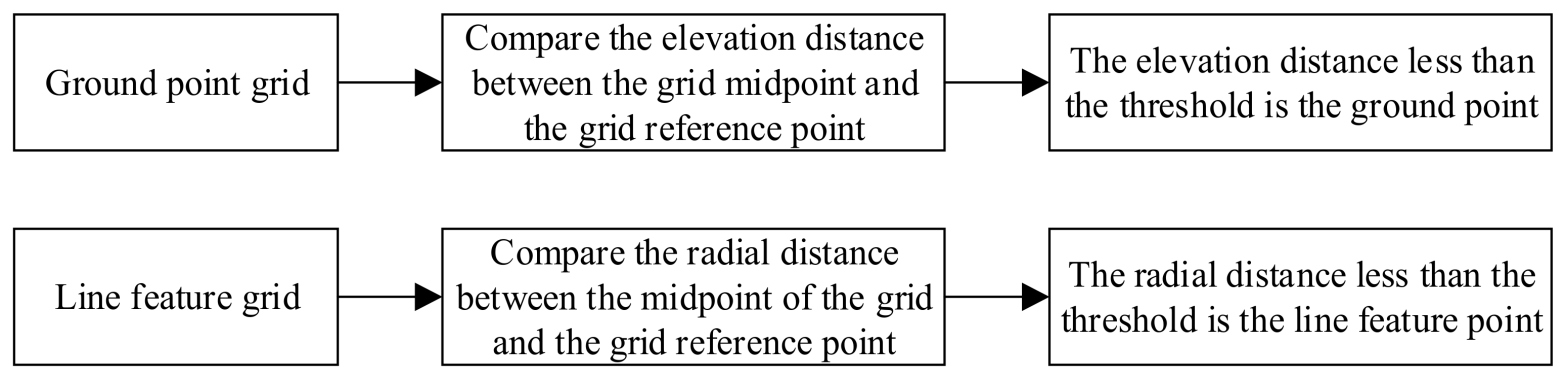
The process of subdividing points in the same class of rasters.

Sector in the raster segmentation and confirm the category; the next need is detailed extraction of raster midpoints, segmented raster with the information, sector number, raster number and belonging to the category. In order to reduce the traversal of the points to find the time in the point cloud projection to calculate the azimuth, according to the same numbering method, using a dictionary plus vector data structure to store the points in the raster in the meticulous division of each point category can quickly locate the current raster points. The process of subdividing the points in the raster of the confirmed category is shown in Figure 5.

Ground point subdivision discrimination is mainly dependent on the height difference, with the reference point being lower than the set threshold, which can be completed after the selection of ground points; the threshold is set according to the accuracy of LiDAR to determine the 16-line LiDAR similar ground point elevation threshold, which is set to 0.06 m. Line feature point subdivision relies on the radial distance of the ring, and the radial threshold set in this paper is 0.1 m. In order to prevent the points from being too dense when extracting line feature points, it is necessary to homogenize the line feature points—each selected line feature point and the previous line feature point for distance determination—select the distance greater than 0.2 m, and at the same time ensure that a raster extracted line feature points less than 4. The judgment distance and the number of feature points are adjusted according to the LiDAR parameters used and the extraction effect, prioritizing the relatively distant points as the final line feature points. After splitting the ground points and line feature points, the remaining point voxels are filtered and used as surface feature points. In the process of point-to-surface alignment, there will be a process of determining whether the plane is flat or not, and this determination will filter out the surface feature points that are not flat.

### 3.2. Scan-to-Map Based Point Cloud Alignment

After completing the point cloud feature extraction, the extracted feature points need to be aligned to obtain the laser odometry at the current moment. In this paper, we use Scan-to-Map’s point cloud inter-frame alignment method [21], which has certain real-time processing capability while ensuring high matching accuracy. Where Scan represents the current frame and Map represents the local map. The local map is stitched together from the point cloud of the previous keyframe as well as the point cloud of the surrounding historical keyframes and is maintained and updated using a sliding window mechanism. By constructing residuals for the current frame and the local map, the ICP algorithm is utilized to accomplish the point cloud alignment task [29].

During the point cloud alignment process, different alignment strategies are used, aiming at different types of feature points. For line features, a point-to-line ICP alignment method is used, while for surface features and ground points, a point-to-surface ICP alignment is used. In this paper, the Levenberg–Marquardt (L-M) method is used to solve the nonlinear least squares problem. In this case, the point-to-line residuals are computed, and the point-to-face residuals are constructed in the same way as LOAM. The residuals of Scan-to-Scan’s point to line are calculated as

(6) $$d_{\varepsilon }=\frac{\left|\left({\tilde{Ρ}}_{\left(k+1,i\right)}^{L}-{\bar{Ρ}}_{\left(k,j\right)}^{L})\times ({\tilde{Ρ}}_{\left(k+1,i\right)}^{L}-{\bar{Ρ}}_{\left(k,l\right)}^{L}\right)\right|}{\left|{\bar{Ρ}}_{\left(k,j\right)}^{L}-{\bar{Ρ}}_{\left(k,l\right)}^{L}\right|}$$

where i is the point to be aligned in the current frame, j, l are the two closest points to i from the previous frame using KD-Tree, and the points, j, l are required not to come from the same line of the laser. The top of the formula can be seen as a cross product of the vector $\vec{il}$ and the vector $\vec{ij}$, the area of the parallelogram. The below the formula is the modulus of the vector $\vec{jl}$, and the residual $d_{\varepsilon }$ from the point to the line can be interpreted as the height of the parallelogram.

The core idea of Scan-to-Map’s line feature residual computation is the same as above, and the vector $\vec{jl}$ is replaced by a feature vector. Unlike Scan-to-Scan, which looks for nearest neighbors in the previous frame, the Scan-to-Map approach looks for nearest neighbors in a local map. Using the five nearest points to point i found in the local map using KD-Tree and making sure that all five points are within 1 m of point i, the covariance matrix and the eigenvalues and eigenvectors corresponding to the covariance matrix are calculated for the nearest neighbor points. If the largest eigenvalue is three times larger than the next largest eigenvalue, the eigenvector corresponding to the largest eigenvalue is considered to be able to represent the line direction of this point cloud, from which the vector $\vec{jl}$ is obtained, and the residuals are calculated in the same way as in Equation (6).

The residuals of the Scan-to-Scan surface features are calculated as

(7) $$d_{\delta }=\frac{\left|({\tilde{Ρ}}_{\left(k+1,i\right)}^{L}-{\bar{Ρ}}_{\left(k,j\right)}^{L})\cdot \left(\left({\bar{Ρ}}_{\left(k,j\right)}^{L}-{\bar{Ρ}}_{\left(k,l\right)}^{L})\times ({\bar{Ρ}}_{\left(k,j\right)}^{L}-{\bar{Ρ}}_{\left(k,m\right)}^{L}\right)\right)\right|}{\left|\left({\bar{Ρ}}_{\left(k,j\right)}^{L}-{\bar{Ρ}}_{\left(k,l\right)}^{L})\times ({\bar{Ρ}}_{\left(k,j\right)}^{L}-{\bar{Ρ}}_{\left(k,m\right)}^{L}\right)\right|}$$

where i is the point to be aligned in the current frame, j, l, m are the three nearest points to the point i from the previous frame using the KD-Tree, and the points j, l are required to be from the same line of the LiDAR, and j, m are from different lines of the LiDAR.

The cross product of the upper and lower parts of Equation (7) represents the area of the parallelogram formed by j, l, m. The upper directional quantity $\vec{ij}$ dot times the area of a parallelogram represents the volume of a hexahedron with the parallelogram formed by j, l, m as the base area and the residual $d_{\delta }$ as the height.

In Scan-to-Map’s face feature alignment, the computational complexity is reduced by using a direct method of solving the plane equation expression, with the residuals expressed as a point-to-face distance formula. This is done by using a KD-Tree to find the five closest points to point i in the local map, and ensuring that all five points are within 1 m of point i. Construct the equation AX = B using five points, where X denotes the unknown quantities $\frac{A}{D}$, $\frac{B}{D}$, $\frac{C}{D}$ in the plane equation $\frac{A}{D}x+\frac{B}{D}y+\frac{C}{D}z+1=0$, solve the plane equation and verify that the resulting plane is flat, ensuring that the distance from any point to the plane is less than 0.1 m. Finally, use the point-to-surface distance formula to represent the residuals.

**Figure 6:**
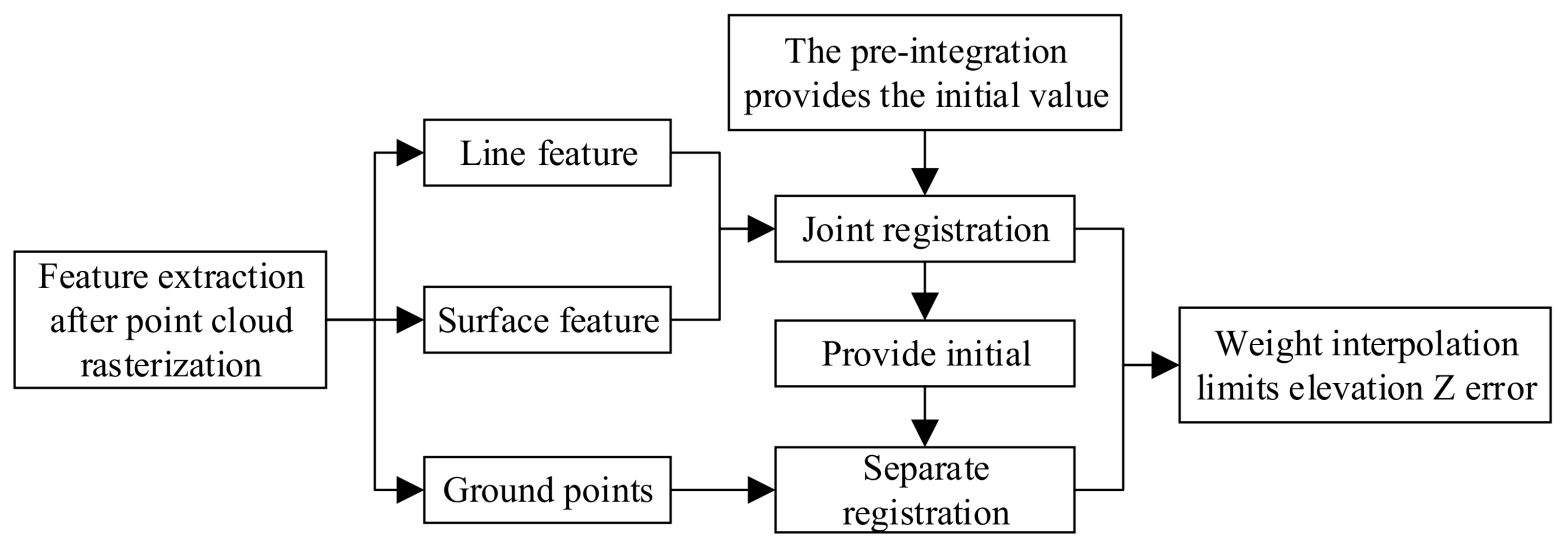
Flow of ground constraint algorithm implementation.

After the point-to-line and point-to-surface residuals are added, the nonlinear least squares problem is constructed and solved using the Levenberg–Marquardt (L-M) method to obtain the updated final laser odometer. In this paper, the method of constructing ground constraints is used to reduce the elevation cumulative error, and the extracted ground feature points are individually aligned using Scan-to-Map’s surface feature solving method. The result obtained from the joint optimization of line features and surface features is used as the initial value for ground point alignment, and a new Z value is estimated based on the height difference between the current frame and the first frame. Finally, the obtained ground point alignment results are weighted and interpolated in combination with the results of the joint alignment. In order to control the interpolation weights to measure the results of ground point alignment, the standard deviation of Roll and Pitch for joint alignment and Roll and Pitch for ground point alignment alone are calculated, respectively. If the difference between roll angle and pitch angle is small, the interpolation weights are increased appropriately, and vice versa, the weights are decreased to ensure the robustness of the system. The flow of the ground constraint algorithm implementation is shown in Figure 6.

The rationale for using the roll/pitch variance difference as a confidence proxy can be understood as follows. When the line-surface registration produces a small roll/pitch variance, the planar surfaces are well-aligned, indicating that the local environment approximately satisfies the ground-plane assumption. In this case, the ground-point alignment (which assumes a flat ground plane) is highly reliable, and its elevation constraint should be weighted more heavily. Conversely, when the roll/pitch variance is large (e.g., on uneven terrain or ramps), the ground-plane assumption is less valid, and the system should rely more on the joint line-surface registration, which does not assume planarity. Formally, let $\sigma_{r,p}^{jo\mathrm{int}}$ and $\sigma_{r,p}^{ground}$ denote the standard deviations of roll and pitch from the joint registration and the ground-only registration, respectively. The interpolation weight α is computed as

(8) $$\alpha =\exp\left(-\frac{\left|\sigma_{r,p}^{jo\mathrm{int}}-\sigma_{r,p}^{ground}\right|}{\beta }\right)$$

where β is a temperature parameter (set to β=0.05 rad in all experiments) controlling the sensitivity of the weighting. When the two variances are similar (indicating consistent alignment), α approaches 1, giving full weight to the ground constraint. When they differ significantly, α decreases, reducing the influence of the potentially unreliable ground constraint. This adaptive strategy is compared against a fixed-weight baseline (α=0.5) in Section 5.5.2, demonstrating superior elevation accuracy.

### 3.3. IMU Preintegration Module Design

The operating frequency of IMUs is usually greater than 100 Hz, which generates a huge amount of data. By pre-integrating IMUs, the workload of repeated calculations in each optimization iteration can be reduced, thus reducing the computational complexity and improving the real-time performance of the system. All IMU observations between the radar keyframes k and k+1, corresponding to the IMU data between $b_{k}$ and $b_{k+1}$, are integrated. Based on the IMU’s position, velocity, and attitude at $b_{k}$ in the world coordinate system at PVQ, the integration is used to obtain the IMU’s position, velocity, and attitude at PVQ at $b_{k+1}$. After obtaining the integration quantities between IMUs from frame k to frame k+1, the noise term is separated out to obtain the quantities to be estimated for each preintegration as shown in Equation (9):

(9) $$\left\{\begin{array}{c} \alpha_{b_{k+1}}^{b_{k}}=\iint_{t\in \left[t_{k}t_{k+1}\right]}(R_{t}^{b_{k}}({\hat{a}}_{t}-b_{a_{t}}-n_{a})dt^{2}\\ \beta_{b_{k+1}}^{b_{k}}=\int_{t\in \left[t_{k},t_{k+1}\right]}R_{t}^{b_{k}}({\hat{a}}_{t}-b_{at}-n_{a})dt\\ \gamma_{b_{k+1}}^{b_{k}}=\int_{t\in \left[t_{k},t_{k+1}\right]}\frac{1}{2}\Omega ({\hat{w}}_{t}-b_{wt}-n_{w})q_{t}^{b_{k}}dt \end{array}\right.$$

where $\alpha_{b_{k+1}}^{b_{k}}$, $\beta_{b_{k+1}}^{b_{k}}$ and $\gamma_{b_{k+1}}^{b_{k}}$ can be interpreted as the pose increment of IMU at $b_{k+1}$ relative to $b_{k}$, and their values are mainly related to the noise of IMU. If the IMU noise at $b_{k+1}$ is not too large, the value at $b_{k+1}$ can be approximated by the linearization method using the value at $b_{k}$ at the previous moment, thus avoiding repeated integration and saving computational resources.

**Figure 7:**
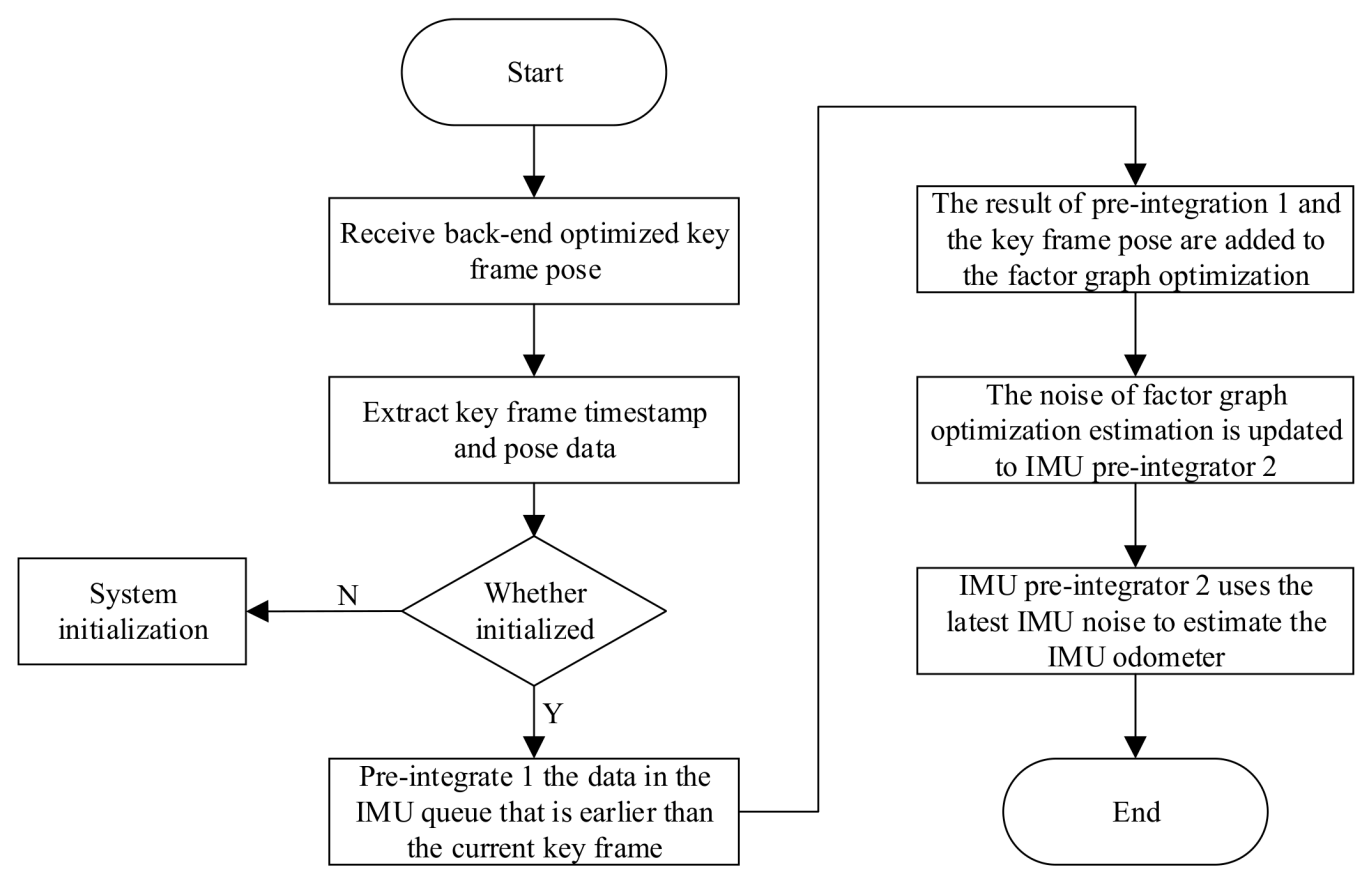
IMU preintegration design.

To ensure the accuracy of preintegration, the IMU noise needs to be re-evaluated after a certain period of time. In this paper, we design two pre-integrators for the pprepreintegrationstem: the first pre-integrator is used to update the IMU noise; when the latest two consecutive keyframe poses estimated by the back-end optimization are received, the IMU data between the two keyframe poses are pre-integrated, and together with the two latest back-end-optimized keyframe poses, they are added into a factor graph for optimization, and the factor graph is used to estimate the IMU noise at the current moment. The second pre-integrator uses the newly estimated IMU noise to pre-integrate the current moment of IMU data in real time to estimate the latest position state of the unmanned vehicle, and the specific design flow is shown in Figure 7. The IMU prpreintegrationesults are used to de-distort the point cloud and provide the initial value for the point cloud alignment, which forms a set of robust IMU pre-integration systems.

The practical implementation of the dual pre-integrator scheme follows these specifications.

Noise Update Frequency: The long-term pre-integrator updates the IMU noise parameters every 2 s, corresponding to 200 IMU samples at the 100 Hz sampling rate. This interval provides a sufficient sliding window for robust noise estimation while maintaining computational efficiency.

Noise Model: The estimated noise model includes both white noise ($n_{a}$ for accelerometer, $n_{g}$ for gyroscope) and bias random walk ($n_{ba}$ for accelerometer bias, $n_{bg}$ for gyroscope bias), following the standard continuous-time IMU stochastic model proposed by Forster et al. [6]. The discrete-time equivalent is used for preintegration covariance propagation.

Covariance Re-Propagation Policy: When noise parameters are updated, the covariance of subsequent ppreintegrationmeasurements is adjusted accordingly. Historical pre-integration factors already inserted into the factor graph are not re-propagated, as this would require re-linearization and significantly increase computational cost. This approximation is valid because the noise update represents a refinement based on accumulated statistical data rather than a correction of past estimates. Empirically, we observe negligible accuracy degradation from this approximation while maintaining real-time performance.

Computational Cost: The short-term pre-integrator runs in real time at 200 Hz with an average cost of 2.28 ms per integration step on the i7-8700 platform (3.7 GHz). The long-term pre-integrator operates at 0.5 Hz (every 2 s) with an average cost of 8.4 ms per update. The total IMU preintegration overhead is 4.56 ms per LiDAR frame (13.9% of the total pipeline runtime), as detailed in Section 5.5.4

### 3.4. Analytical Foundation of the Proposed Framework

#### 3.4.1. Dual-Time-Scale Constraint Mechanism

State estimation in LiDAR–IMU SLAM aims to continuously estimate the platform pose by tightly coupling high-frequency inertial measurements with geometrically reliable observations obtained from LiDAR scans. The system state at time k can be represented as

(10) $$x_{k}=\left[R_{k},v_{k},p_{k},b_{g,k},b_{a,k}\right]$$

where $R_{k}$, $v_{k}$, $p_{k}$ and denote the orientation, velocity, and position of the platform, respectively, while $b_{g,k}$ and $b_{a,k}$ represent the gyroscope and accelerometer biases. This state formulation has been widely adopted in modern LiDAR–IMU SLAM systems based on factor graph optimization and IMU preintegration [4,6,18,22,30].

IMU preintegration has become one of the fundamental techniques for modern tightly coupled state estimation because it efficiently summarizes numerous inertial measurements into a compact motion constraint while preserving first-order uncertainty propagation [6]. Representative systems, including LIO-SAM [18], FAST-LIO [19], FAST-LIO2 [20], and Point-LIO [21], all employ preintegrated inertial constraints to bridge consecutive keyframes and improve the robustness of pose estimation. However, these methods generally construct a single preintegration constraint between adjacent optimization states, assuming that one temporal integration interval can simultaneously characterize both short-term platform motion and long-term trajectory consistency.

From the perspective of estimation theory, the above assumption introduces an inherent contradiction. High-frequency vehicle motion mainly contains rapidly varying dynamic information that requires prompt state propagation, whereas accumulated inertial biases and integration errors evolve over much longer temporal scales [6,22]. Consequently, a single preintegration interval must simultaneously satisfy two different estimation objectives: preserving local motion continuity while suppressing long-term drift accumulation. Since these two objectives possess significantly different temporal characteristics, improving one aspect often compromises the other, particularly during long-duration navigation or in environments where LiDAR observations become temporarily unreliable.

To alleviate this limitation, the proposed framework introduces a dual-time-scale preintegration mechanism, in which two complementary inertial constraints with different temporal characteristics are jointly incorporated into the factor graph optimization. Unlike conventional approaches that simply increase the number of inertial observations, the proposed strategy establishes two estimation constraints serving different roles in the optimization process.

**Definition** **1** **(Dual-Time-Scale Inertial Constraint).**  *Let* $F_{s}$ *and* $F_{l}$ *denote the short-term and long-term IMU preintegration factors, respectively. The proposed dual-time-scale inertial constraint is defined as the joint optimization of these two complementary factors within the same factor graph, where* $F_{s}$ *mainly preserves local motion continuity and* $F_{l}$ *enforces long-term trajectory consistency.*

**Figure 8:**
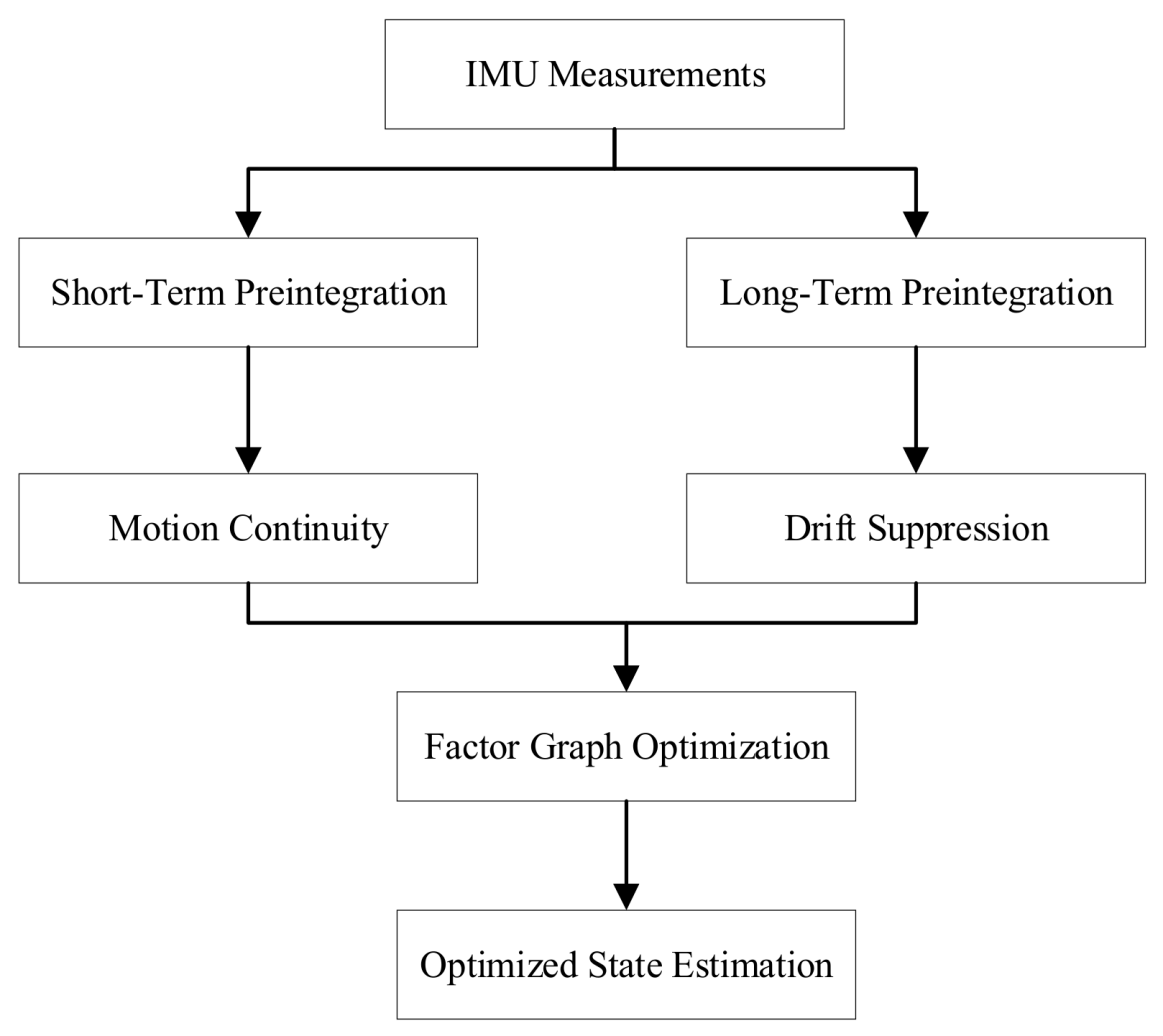
Hierarchical dual-time-scale inertial constraint mechanism.

The hierarchical organization of the proposed dual-time-scale inertial constraints is illustrated in Figure 8, where the short-term and long-term preintegration branches jointly provide complementary motion constraints for factor graph optimization.

The first constraint is generated from short-term preintegration, whose primary objective is to preserve high-frequency motion continuity. Because the integration interval is relatively short, accumulated bias and numerical integration errors remain limited, enabling accurate prediction of rapidly changing vehicle motion and providing stable initialization for LiDAR scan registration.

The second constraint is established through long-term preintegration, which spans a larger temporal interval and introduces additional temporal consistency into the optimization process. Although longer integration intervals inevitably accumulate larger inertial uncertainty, they simultaneously provide stronger correlations among neighboring keyframes. Through joint optimization with LiDAR, GPS, and loop-closure observations, the long-term constraint effectively suppresses slowly varying estimation errors, especially accumulated position drift and attitude deviation that are difficult to eliminate using only local observations.

Consequently, the proposed framework forms a hierarchical dual-time-scale estimation mechanism. The short-term preintegration primarily captures local dynamic motion and guarantees rapid state convergence, whereas the long-term preintegration constrains low-frequency drift and improves global trajectory consistency. These two constraints are therefore complementary rather than redundant, allowing different error components to be constrained at their corresponding temporal scales.

From the viewpoint of factor graph optimization, the proposed framework does not merely introduce additional IMU factors. Instead, it constructs a multi-scale inertial constraint architecture that enhances the temporal observability of the estimation system by jointly exploiting local motion continuity and long-term trajectory consistency. As illustrated in Figure 8, the proposed framework establishes a hierarchical dual-time-scale estimation architecture in which local motion continuity and long-term trajectory consistency are jointly exploited during optimization. This analytical framework serves as the theoretical foundation for the subsequent information matrix analysis and error propagation analysis.

#### 3.4.2. Information Enhancement Analysis

In graph-based SLAM systems, the estimation accuracy is fundamentally determined by the amount and quality of measurement information incorporated into the optimization process. Each observation introduces an independent residual constraint, and the optimal system state is obtained by minimizing the weighted nonlinear least-squares objective [22,30].

For the proposed framework, the overall optimization objective can be expressed as

(11) $$\underset{x}{\min}{\sum_{i}\left\|r_{i}(x)\right\|}^{2}w_{i}$$

where $r_{i}(x)$ denotes the residual associated with the i th sensor constraint, including LiDAR registration, IMU preintegration, GPS observations, loop-closure constraints, and the proposed dual-time-scale inertial constraints, while $w_{i}$ represents the corresponding information matrix.

After linearization around the current state estimate, the optimization problem can be approximated by

(12) H△x=b

where

(13) $$H=\sum_{i}J_{i}^{T}w_{i}J_{i}$$

is the Hessian (information) matrix, and $J_{i}$ denotes the Jacobian matrix of the corresponding residual function [22,30]. The estimation covariance is approximately given by

(14) $$P\approx H^{-1}$$

indicating that the quality of state estimation is directly related to the information contained in the optimization problem.

Unlike conventional LiDAR–IMU SLAM systems that employ only a single preintegration constraint between adjacent keyframes [18,19,20,21], the proposed framework jointly introduces two complementary inertial constraints operating at different temporal scales. Consequently, the corresponding Hessian matrix can be expressed as

(15) $$H_{dual}=H_{base}+△H$$

where

(16) $$△H=J_{dual}^{T}w_{dual}J_{dual}$$

represents the additional information contributed by the long-term preintegration constraint.

Since the weighting matrix $w_{dual}$ is positive semi-definite, the additional informationterm satisfies

(17) △H≥0

which leads to

(18) $$H_{dual}\geq H_{base}$$

This result indicates that the proposed dual-time-scale framework introduces additional estimation information without violating the consistency of the original optimization problem. Instead of repeatedly imposing identical inertial observations, the long-term preintegration constraint complements the short-term constraint by establishing additional temporal correlations among optimization states.

From the perspective of estimation theory, increasing the information matrix generally leads to a reduction in state uncertainty,

(19) $$P_{dual}=H_{dual}^{-1}\leq H_{base}^{-1}=P_{base}$$

suggesting that the proposed framework theoretically provides lower estimation covariance than the conventional single-preintegration formulation. Although the actual localization accuracy also depends on measurement quality, sensor noise, and environmental conditions, the enhanced information structure established by the proposed framework provides a stronger theoretical basis for improving trajectory consistency and reducing long-term localization drift.

Therefore, the proposed dual-time-scale preintegration mechanism should be interpreted as an information enhancement strategy rather than simply increasing the number of inertial factors. By jointly exploiting complementary temporal constraints, the proposed framework improves the information distribution of the factor graph and establishes a more informative optimization problem for subsequent state estimation.

**Proposition** **1**. *Under the assumption that the additional long-term preintegration provides non-re dundant temporal constraints, the proposed dual-time-scale formulation produces an information matrix that is positive semi-definite with respect to the conventional single-preintegration formulation, thereby reducing the theoretical lower bound of estimation covariance.*

The conclusion follows directly from the additive property of the Hessian matrix in nonlinear least-squares optimization, where each independent residual contributes a positive semi-definite information term.

#### 3.4.3. Error Propagation Analysis

Although LiDAR observations provide accurate geometric constraints, the long-term localization accuracy of tightly coupled LiDAR–IMU SLAM systems is still fundamentally limited by the propagation of inertial errors. As the platform continuously moves, measurement noise, IMU bias, numerical integration errors, and imperfect scan registration gradually accumulate in the state estimation process, resulting in increasing trajectory drift over time [4,6,18,23].

Considering the system state error at time k,

(20) $$\delta x_{k}=x_{k}-{\hat{x}}_{k}$$

its first-order propagation between two consecutive optimization states can be approxi-mated as

(21) $$\delta x_{k+1}=F_{k}\delta x_{k}-G_{k}w_{k}$$

where $F_{k}$ denotes the state transition matrix, $G_{k}$ represents the noise propagation matrix, and $w_{k}$ is the zero-mean Gaussian process noise associated with inertial measurements [6,23]. This formulation indicates that the estimation error at each time step depends not only on the current sensor noise but also on the accumulated error inherited from previous states.

In conventional single-preintegration frameworks, the propagated inertial error is mainly corrected by local LiDAR registration and occasional loop-closure optimization [18]. During long-distance navigation or in feature-degraded environments, however, geometric observations may become insufficient to completely compensate for the accumulated inertial drift. Consequently, the propagated estimation error gradually increases, particularly along the vertical direction where geometric constraints are relatively weak.

**Figure 9:**
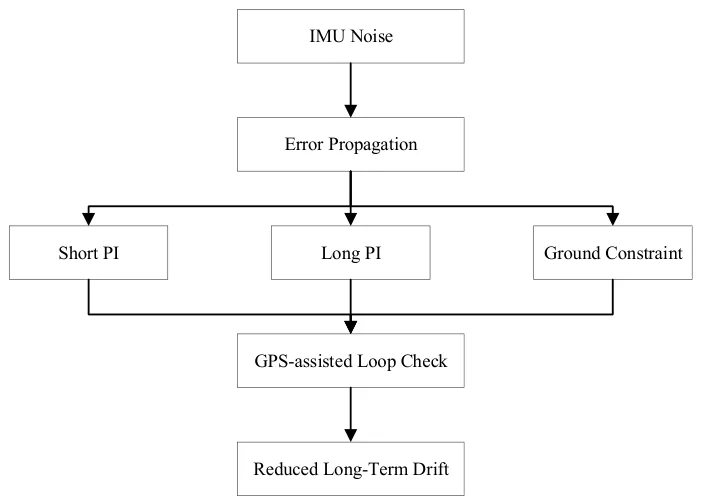
Error propagation suppression mechanism of the proposed framework.

The proposed framework suppresses this cumulative error propagation through three complementary mechanisms. As illustrated in Figure 9, the proposed framework suppresses accumulated estimation errors through three complementary pathways, namely short-term motion stabilization, long-term temporal consistency enhancement, and adaptive geometric correction. Instead of correcting individual measurements independently, these mechanisms jointly interrupt the continuous propagation of localization errors across successive optimization states.

First, the short-term preintegration continuously provides accurate local motion prediction, thereby limiting the accumulation of high-frequency integration errors. Second, the long-term preintegration introduces additional temporal consistency among neighboring optimization states, effectively reducing the propagation of low-frequency inertial bias. Finally, the proposed adaptive ground-constrained optimization further establishes stable geometric constraints in the vertical direction, preventing accumulated elevation errors from continuously propagating throughout the trajectory.

Consequently, the state error propagation of the proposed framework can be conceptually expressed as

(22) $$\delta x_{k+1}=(F_{k}-K_{k})\delta x_{k}+G_{k}w_{k}$$

where $K_{k}$ represents the equivalent correction introduced by the complementary temporal constraints and the adaptive ground observations. Compared with the conventional formulation, the proposed framework continuously suppresses the propagation of accumulated estimation errors by providing additional temporal and geometric constraints during optimization.

From the viewpoint of estimation theory, the proposed dual-time-scale framework does not directly eliminate sensor noise; instead, it effectively reduces the amplification of propagated errors throughout successive optimization iterations. Since accumulated drift originates from continuous error propagation rather than individual measurements, suppressing the propagation process is fundamentally more effective than correcting isolated estimation errors after they occur.

Furthermore, the GPS-assisted loop verification proposed in this work introduces additional absolute positioning information during loop-closure validation. Although GPS observations are not continuously involved in front-end optimization, they effectively constrain large-scale global drift whenever reliable GPS measurements are available, thereby reducing the risk of incorrect loop closures caused by accumulated localization errors. Consequently, the proposed framework jointly exploits inertial continuity, geometric consistency, and absolute positioning information to achieve more robust long-term localization.

As summarized in Figure 9, the proposed framework establishes a hierarchical error suppression architecture that integrates inertial continuity, geometric consistency, and GPS-assisted global correction. This multi-level correction mechanism provides a theoretical explanation for the improved localization robustness and reduced long-term drift observed in the experimental evaluation presented in Section 5.

**Proposition** **2**. *Under identical sensor noise conditions, the proposed framework reduces the propagation of accumulated estimation errors by jointly introducing complementary temporal constraints and adaptive geometric corrections. Consequently, the long-term localization drift is theoretically bounded more tightly than that of the conventional single-preintegration framework.*

The conclusion follows from the combined effects of information enhancement (Section 3.4.2) and the complementary correction mechanisms introduced during state propagation. The experimental results in Section 5 further validate this theoretical observation.

#### 3.4.4. Computational Complexity and Real-Time Analysis

Besides estimation accuracy, computational efficiency is another critical requirement for real-time LiDAR–IMU SLAM systems deployed on autonomous platforms. Since the proposed framework introduces additional raster feature extraction, dual-time-scale IMU preintegration, adaptive ground-constrained optimization, and GPS-assisted loop verification, it is necessary to analyze whether these additional modules significantly increase the computational burden.

The overall computational pipeline of the proposed framework mainly consists of five sequential stages, including raster feature extraction, inertial state propagation, factor graph optimization, loop-closure verification, and map update. The theoretical computational complexity of the major functional modules is summarized in Table 1, which provides an overall comparison of the computational characteristics introduced by each component of the proposed framework. Let $N_{p}$ denote the number of LiDAR points in a scan, $N_{f}$ the number of extracted feature points, $N_{k}$ the number of optimization variables, and $N_{l}$ the number of candidate loop closures. The computational complexity of each module can be approximately summarized below.

The raster feature extraction module performs neighborhood traversal and local geometric evaluation for each point. Therefore, its computational complexity is approximately $O(N_{p})$ which scales linearly with the number of LiDAR measurements.

The proposed dual-time-scale preintegration performs two independent inertial integrations using the same IMU measurement stream. Since IMU preintegration itself is a recursive process with constant computational cost per measurement [6,18], introducing an additional preintegration branch only results in a constant-factor increase. Therefore, the overall computational complexity remains $O(N_{imu})$ where $N_{imu}$ denotes the number of IMU measurements received during one LiDAR scan interval.

For graph optimization, the proposed framework adopts the factor graph formulation implemented in GTSAM [22,30]. Owing to the sparsity of the Jacobian matrix, the incremental optimization complexity mainly depends on the number of newly added factors rather than the total trajectory length. Consequently, the additional dual-time-scale inertial constraints introduce only a limited increase in computational cost while preserving the sparse structure of the optimization problem.

The proposed adaptive ground-constrained optimization is only applied to the selected ground feature subset rather than the entire point cloud. Since the number of ground features is considerably smaller than the total number of LiDAR points, the additional computational overhead is negligible compared with the front-end scan registration process.

Similarly, the GPS-assisted loop verification module is activated only when a candidate loop closure has already been detected. Therefore, instead of continuously participating in front-end state estimation, the GPS verification introduces only occasional computation during loop validation, leading to a relatively small average computational cost throughout the entire mapping process.

Overall, the computational complexity of the proposed framework can be approximated as

(23) $$O(N_{p})+O(N_{imu})+O(N_{opt})+O(N_{l})$$

where $N_{opt}$ denotes the complexity of sparse graph optimization. Since all newly introduced modules either preserve linear computational complexity or operate only on selected observations, the proposed framework maintains the same asymptotic computational complexity as conventional graph-based LiDAR–IMU SLAM systems.

Furthermore, as experimentally demonstrated in the runtime evaluation presented in Section 5.5.3, the additional computational overhead introduced by the proposed modules remains limited. The average processing frequency still satisfies the real-time requirements of autonomous navigation, indicating that the proposed framework achieves improved localization accuracy without sacrificing computational efficiency.

As summarized in Table 1, all newly introduced modules either preserve linear computational complexity or only introduce limited additional computation during sparse optimization. Combined with the runtime evaluation presented in Section 5.5.3, these results demonstrate that the proposed framework achieves improved localization accuracy while maintaining real-time performance suitable for autonomous navigation.

## 4. Multi-Sensor Fusion SLAM Back-End Design

### 4.1. ESKF-Based GPS and IMU Data Fusion

GPS produces a hopping drift problem in the positioning results during the period between the loss of signal due to being obscured by tall buildings or trees and the full recovery of the signal. In order to improve the GPS positioning accuracy, this paper fuses it with high-frequency IMU data using error state-based Kalman filtering (ESKF). ESKF decomposes the system state into real, nominal, and error states, and uses the error state to correct the nominal state for a more efficient estimation of the real state.

The nominal state of the IMU is defined as

(24) $$x={\left[p^{T},v^{T},q^{T},b_{a}^{T},b_{g}^{T}\right]}^{T}$$

where $p\in {\mathbb{R}}^{3}$ is the position, $v\in {\mathbb{R}}^{3}$ is the velocity, q∈SO(3) is the orientation quaternion, $b_{a}\in {\mathbb{R}}^{3}$ is the accelerometer bias, and $b_{g}\in {\mathbb{R}}^{3}$ is the gyroscope bias. The corresponding error state is defined as

(25) $$\delta x={\left[\delta_{p}^{T},\delta_{v}^{T},{\delta \theta }^{T},{\delta b}_{a}^{T},{\delta b}_{g}^{T}\right]}^{T}$$

where δθ uses a minimal 3-parameter representation for orientation errors via the exponential map $R\approx \hat{R}\cdot Exp(\delta \theta )$ where $\hat{R}$ is the nominal rotation matrix and Exp(⋅) maps the rotation vector to SO(3).

Construct a nominal state motion model of the IMU without considering the effect of noise and integrate the raw IMU data:

(26) $$\begin{array}{l} R_{k+1}=q\left\{{\hat{\omega }}_{k}-b_{g,k}\right\}⊗R_{k}\\ v_{k+1}=v_{k}+g\cdot △t+R_{k}\cdot ({\hat{a}}_{k}-b_{a,k})\cdot △t\\ p_{k+1}=p_{k}+v_{k}\cdot △t+(1/2)g\cdot △t^{2}+(1/2)R_{k}\cdot ({\hat{a}}_{k}-b_{a,k})\cdot △t^{2} \end{array}$$

where $\hat{a}$ denotes accelerometer measurements, $\hat{\omega }$ denotes gyroscope measurements, and $q\left\{\cdot \right\}$ denotes the conversion of the rotation vectors in $\left\{\cdot \right\}$ to the corresponding quaternions.

The continuous-time error state dynamics are obtained by linearizing the IMU kinematics in Equation (26) around the nominal state,

(27) $$\dot{\delta }x=F_{c}\delta x+G_{c}n$$

where $n={\left[n_{a}^{T},n_{g}^{T},n_{b_{a}}^{T},n_{b_{g}}^{T}\right]}^{T}$ collects the accelerometer white noise $n_{a}$, gyroscope white noise $n_{g}$, and the corresponding bias random walks $n_{b_{a}}$, $n_{b_{g}}$. The continuous-time error state transition matrix $F_{c}\in {\mathbb{R}}^{15\times 15}$ and noise mapping matrix $G_{c}\in {\mathbb{R}}^{15\times 12}$ take the form

(28) $$F_{c}=\left[\begin{array}{ccccc} O_{3} & I_{3} & O_{3} & O_{3} & O_{3}\\ O_{3} & O_{3} & -\hat{R}{\left(\hat{a}-{\hat{b}}_{a}\right)}_{\times } & -\hat{R} & O_{3}\\ O_{3} & O_{3} & -{\left(\hat{\omega }-{\hat{b}}_{g}\right)}_{\times } & O_{3} & -I_{3}\\ O_{3} & O_{3} & O_{3} & O_{3} & O_{3}\\ O_{3} & O_{3} & O_{3} & O_{3} & O_{3} \end{array}\right]$$

(29) $$G_{c}=\left[\begin{array}{cccc} O_{3} & O_{3} & O_{3} & O_{3}\\ -\hat{R} & O_{3} & O_{3} & O_{3}\\ O_{3} & -I_{3} & O_{3} & O_{3}\\ O_{3} & O_{3} & I_{3} & O_{3}\\ O_{3} & O_{3} & O_{3} & I_{3} \end{array}\right]$$

in which ${\left[\cdot \right]}_{\times }$ denotes the skew-symmetric matrix operator, $\hat{a}$ and $\hat{\omega }$ are the measured acceleration and angular velocity at the nominal state, and $\hat{R}$ is the nominal rotation matrix.

For a small propagation interval △t, the discrete-time transition matrix $F_{k}$ is approximated by the first-order Taylor expansion of the matrix exponential:

(30) $$F_{k}=I_{15}+F_{c}\cdot △t$$

This first-order discretization is standard for high-rate IMU propagation (e.g., 100–200 Hz) and preserves the computational efficiency required for real-time operation. The system error state prediction equation for discrete time is then:

(31) $$\delta x_{k+1|k}=F_{k}\cdot \delta x_{k|k}+w_{k}$$

The discrete-time process noise covariance follows from the continuous-time noise model:

(32) $$Q_{k}\approx G_{c}Q_{c}G_{c}^{T}\cdot △t$$

(33) $$Q_{c}=diag(\sigma_{a}^{2}I_{3},\sigma_{g}^{2}I_{3},\sigma_{b_{a}}^{2}I_{3},\sigma_{b_{g}}^{2}I_{3})$$

where $Q_{c}$ contains the continuous-time power spectral densities of the IMU noise parameters. The covariance matrix is predicted to be

(34) $$P_{k|k-1}=F_{k}P_{k-1|k-1}F_{k}^{T}+Q_{k}$$

(35) $$P_{k|k}=(I-K\cdot H)P_{k|k-1}$$

Using the GeographicLib library to transfer the raw GPS data under the UTM coordinate system, the position observation equation for GPS can be defined as

(36) $$z_{GPS}=t+R\cdot p_{GPS\leftarrow IMU}-p_{GPS}$$

where $p_{GPS}$ is the position of the GPS sensor under the Map coordinate system of the center of the rear axle of the unmanned vehicle, t and R are the translation vectors and rotation matrices of the IMU sensor to the Map coordinate system of the center of the rear axle of the unmanned vehicle, which is also relative to the GPS and the IMU, and $p_{GPS\leftarrow IMU}$ is the position of the GPS sensor under the IMU coordinate system. This aligns the GPS trajectory with the unmanned vehicle positioning trajectory, from which the Jacobi matrix of GPS observations relative to the error state in the global coordinate system can be calculated.

Since the GPS directly observes the position component, the measurement model Jacobian $H_{GPS}=\partial z_{GPS}/\partial \delta x$ reduces to a simple selection matrix:

(37) $$H_{GPS}=\left[I_{3},O_{3},O_{3},O_{3},O_{3}\right]\in {\mathbb{R}}^{3\times 15}$$

The coordinate transformation in Equation (36) ensures that the GPS position is expressed in the same global frame as the LiDAR-IMU state estimate, so that $H_{GPS}$ operates directly on δp without additional rotation terms. The Jacobi matrix of GPS observations relative to the error state in the global coordinate system can be calculated as

(38) $$H=\left[H_{GPS},O_{3\times 12}\right]$$

The above derivation yields the update formula for ESKF as

(39) $$K=P_{k|k-1}H^{T}{\left(HP_{k|k-1}H^{T}+V\right)}^{-1}$$

(40) $$\delta {\hat{x}}_{k|k}=K(z_{GPS}-H\cdot \delta x_{k|k-1})$$

(41) $$P_{k|k}=(I-K\cdot H)P_{k|k-1}$$

where K is the Kalman gain, P is the predicted covariance matrix, the final P is the corrected covariance matrix, H is the Jacobi matrix of the observation relative to the error state, and V is the covariance matrix of the GPS observations.

State merging, which combines the error state and the nominal state, corrects for the drift of the nominal state over time and obtains the optimal unbiased estimate of the actual state:

(42) $$x\leftarrow x⊕\hat{\delta }x$$

Reset the error states and covariance matrix:

(43) $$\hat{\delta }x\leftarrow 0$$

(44) $$P\leftarrow GPG^{T}$$

where G is the unit matrix. The ESKF-fused GPS-IMU state estimate is incorporated into the back-end factor graph as a high-precision global pose factor, jointly optimizing the global trajectory together with LiDAR odometry factors and loop-closure constraints. This cascaded architecture leverages the tightly coupled ESKF front-end for high-frequency local state estimation and the factor-graph back-end for globally consistent multi-session trajectory optimization.

In urban environments, GPS signals are susceptible to degradation due to multipath effects, signal blockage by buildings, and atmospheric interference. To ensure robust operation, our system implements a GPS signal quality assessment module that monitors the following indicators in real time: (1) the number of visible satellites (minimum 4 required); (2) the horizontal dilution of precision (HDOP, threshold < 2.0); and (3) the reported covariance of the GPS position estimate (threshold $\sigma_{\tau }$ = 0.6). When any of these indicators falls below the acceptable range, the system automatically switches to LiDAR-IMU-only mode by disabling GPS factor injection into the factor graph. The ESKF continues to provide accurate local pose estimation using LiDAR and IMU data alone, and the factor graph maintains consistency through LiDAR odometry and IMU preintegration factors. Once GPS quality recovers (all indicators return to acceptable ranges for at least 3 consecutive seconds), GPS factors are re-enabled. This fallback mechanism ensures that temporary GPS degradation (e.g., in tunnels or urban canyons) does not corrupt the global trajectory estimate.

### 4.2. Loop Closure Detection with Scan Context Incorporating GPS Constraints

**Figure 10:**
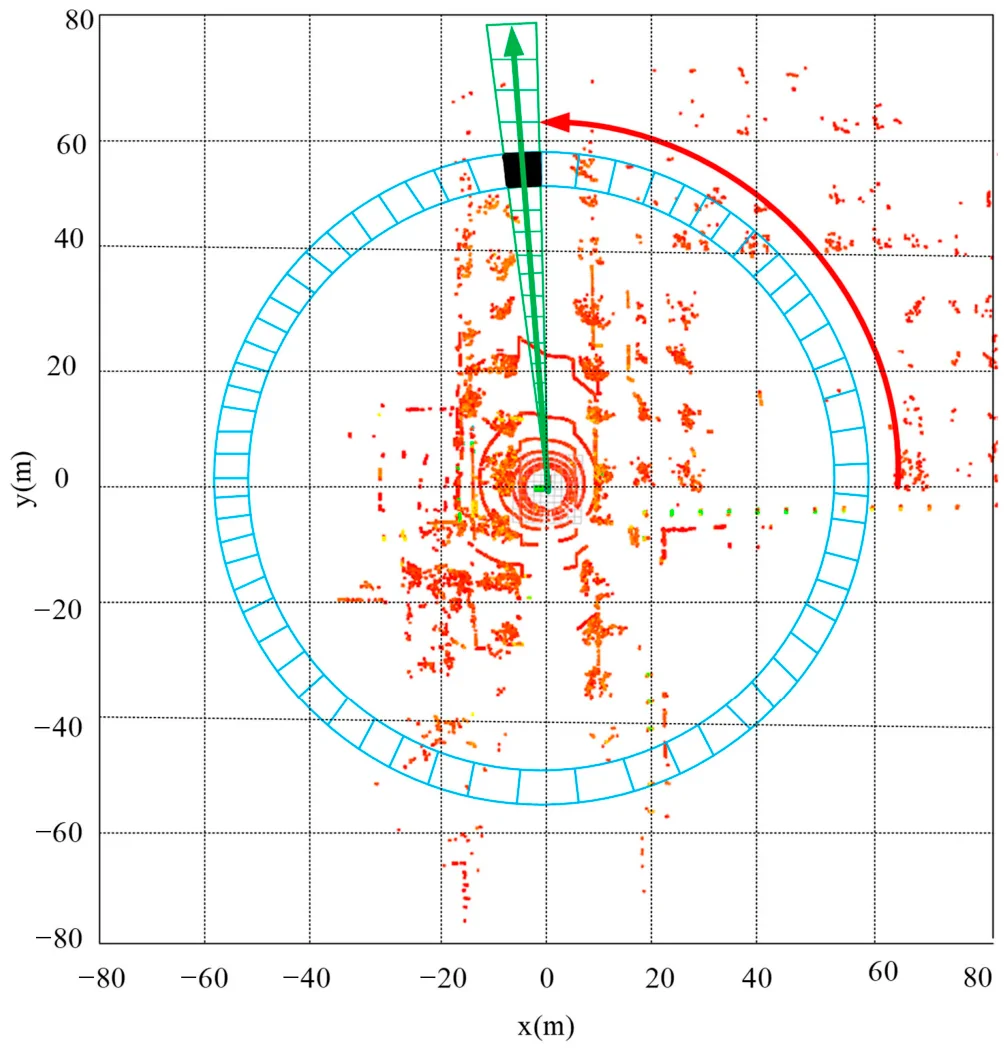
Key frame point cloud segmentation.

In this paper, loop closure detection is designed to eliminate the cumulative error generated by the robot during long-time motion. This paper uses Scan Context as a back-end loop closure detection method. The core idea of Scan Context is to differentiate the point cloud into a sector, continue to segment it into a circle along the vertical direction, and finally use the raster value to represent the Scan Context descriptor, and the segmentation process is shown in Figure 10.

**Figure 11:**
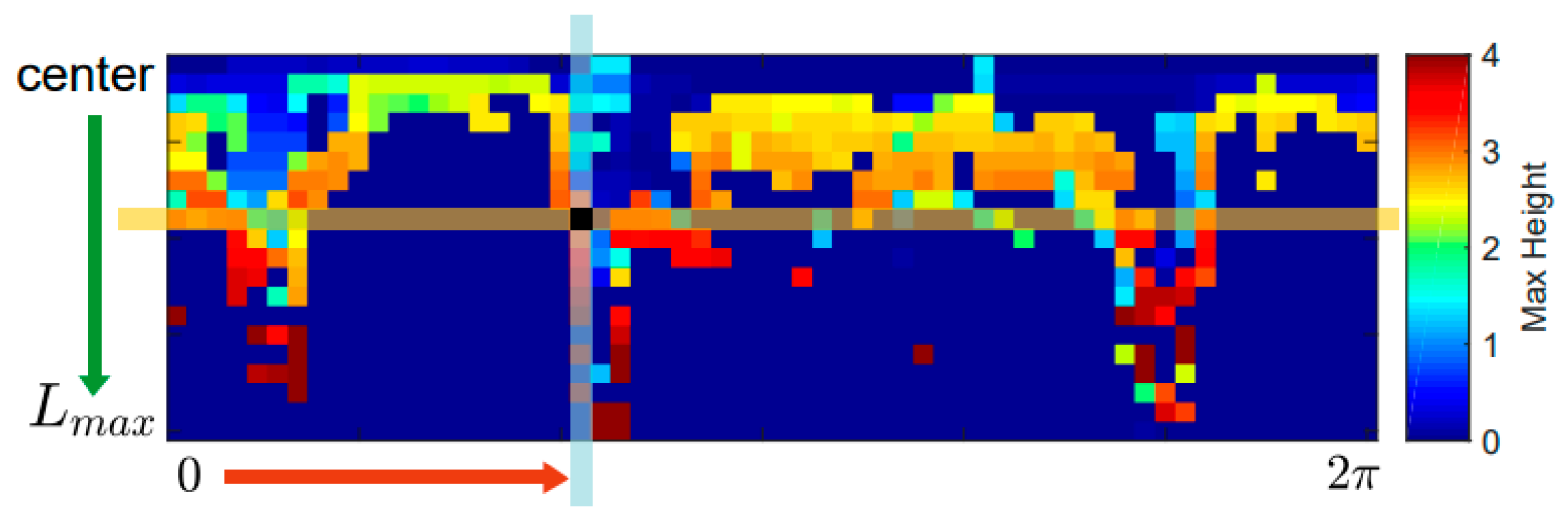
Scan Context global descriptor.

Firstly, taking the radar coordinate system as the origin, the point cloud space is divided into $N_{r}$ circles in the blue part counterclockwise along the direction of the green arrow in the direction of increasing radius Figure 8, which is taken as $N_{r}$ = 20 in this paper. $L_{\max}$ = 100 m indicates the farthest detection distance of the laser, then the width of each circle $d_{r}=L_{\max}/N_{r}$. After splitting the circle, it also needs to be divided into equal parts of $N_{s}$, in this paper $N_{s}$ = 60, so that the set of laser points Ρ after splitting can be expressed:

(45) $$Ρ=\underset{i\in [N_{r}],j\in [N_{s}]}{\cup }{Ρ}_{ij}$$

where $P_{ij}$ denotes the set of points in the segmentation cell of the i th circle, j th sector. After dividing the raster and iterating through each point in the raster, take the point with the maximum height to represent the current raster. As shown in Figure 11, the global descriptor for Scan Context is a 20×60 matrix. Each row in the matrix represents a circle, each column represents a sector, and the value in the matrix is the maximum value of the height of the point in the raster. Using the segmentation method described above, the 3D point cloud information is downscaled to 2D to reduce the consumption of computational and memory resources.

**Figure 12:**
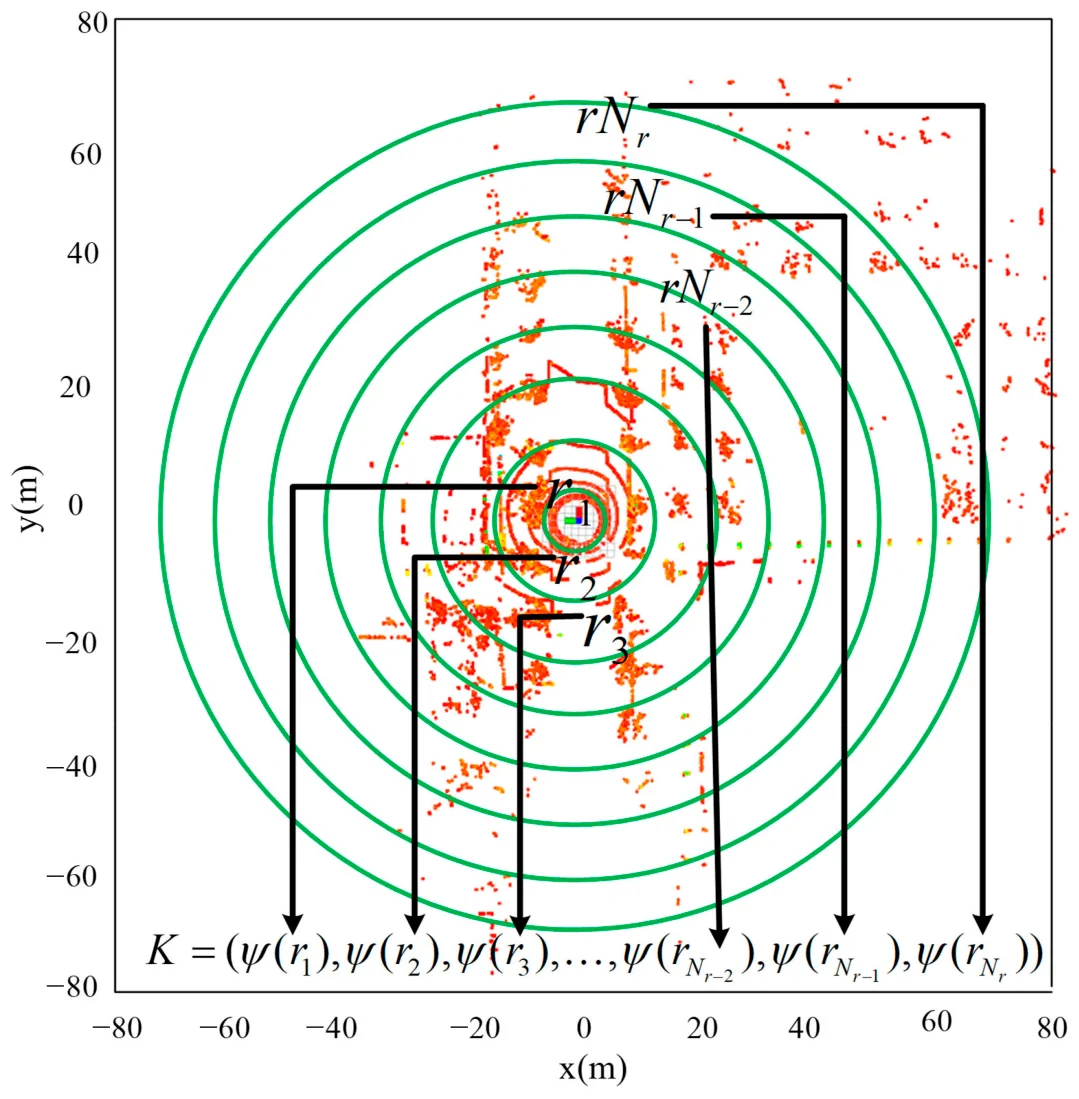
Scan Context descriptor dimensionality reduction.

Although Scan Context segmentation is able to downscale a 3D point cloud to a 2D matrix to significantly reduce the amount of data, searching for similar frames is still time-consuming when the number of keyframes is high. To facilitate similar frame search using KD-Tree, the descriptor needs to be further compressed into a one-dimensional vector of Ring Keys. The specific compression method is to count the percentage of rasters with zero value in each sector to achieve dimensionality reduction, thus generating a one-dimensional vector for each frame,

(46) $$K=(\psi (r_{1}),\ldots ,\psi (r_{N_{r}})),where\psi :r_{i}\to \mathbb{R}$$

where $\psi (r_{i})$ is

(47) $$\psi (r_{i})=\frac{||r_{i}|{|}_{0}}{N_{s}}$$

where $\left\|r_{i}\right\|$ denotes the number of non-empty split cells in the circle corresponding to the radius $r_{i}$. After dimensionality reduction, one-dimensional vectors of all historical keyframes are constructed into a KD-Tree, and the KD-Tree is used to find all the candidate historical keyframes that coarsely match the current frame, and then the Scan Context is used to compute the similarity accuracy scores of the current frame and all the candidate historical keyframes. The frames with scores less than a set threshold are identified as loop closure edges, and the above dimensionality reduction approach reduces the loop closure edge search time to improve efficiency, and Figure 12 shows the 2D descriptor compression process.

Scan Context calculates the similarity score in a manner similar to calculating the mean of the cosine distances of multiple vectors. Notate the Scan Context generated for the current frame as $I^{q}$, where $c_{j}^{q}$ denotes the j th column in the $I^{q}$. Notate the Scan Context generated for the history frame as $I^{c}$, where $c_{j}^{c}$ denotes the j th column in $I^{c}$. The similarity formula for Scan Context is

(48) $$d(I^{q},I^{c})=\frac{1}{N_{s}}\sum_{j=1}^{N_{s}}\left(1-\frac{c_{j}^{q}\cdot c_{j}^{c}}{\left\|c_{j}^{q}\right\|\left\|c_{j}^{c}\right\|}\right)$$

Note that when both columns $c_{j}^{q}$ and $c_{j}^{c}$ are simultaneously empty (i.e., no laser points fall in the corresponding ring sector), the denominator $\left\|c_{j}^{q}\right\|\left\|c_{j}^{c}\right\|$ becomes zero, rendering the similarity score undefined. In practice, this degenerate case is handled by excluding such column pairs from the summation, which is equivalent to assigning a similarity contribution of zero for those columns. This treatment is consistent with the original Scan Context implementation [28] and does not affect loop closure detection performance, since real-world LiDAR scans typically yield a sufficient number of non-empty sectors.

When the similarity score is less than a set threshold, the history frame is considered to be the current loop closure frame, and the two frames of the point cloud are aligned by ICP. The alignment here also uses Scan-to-Map, where Scan is the current frame and Map is the point cloud map constructed from the loop closure frame and surrounding keyframes. The alignment results are added to the factor graph to update the global position and realize loop closure detection to eliminate the cumulative error problem generated by SLAM during motion.

In the Scan Context loop closure detection algorithm, the original keyframes are downscaled when calculating the Scan Context descriptors, and only the maximum value is taken as the descriptor value, which will lose much important information and thus easily generate false loop closures. The experimental environment in this paper is a structured urban scene, and due to the high number of similar frames, Scan Context will have a false loop closure situation with high scores due to scene similarity.

**Figure 13:**
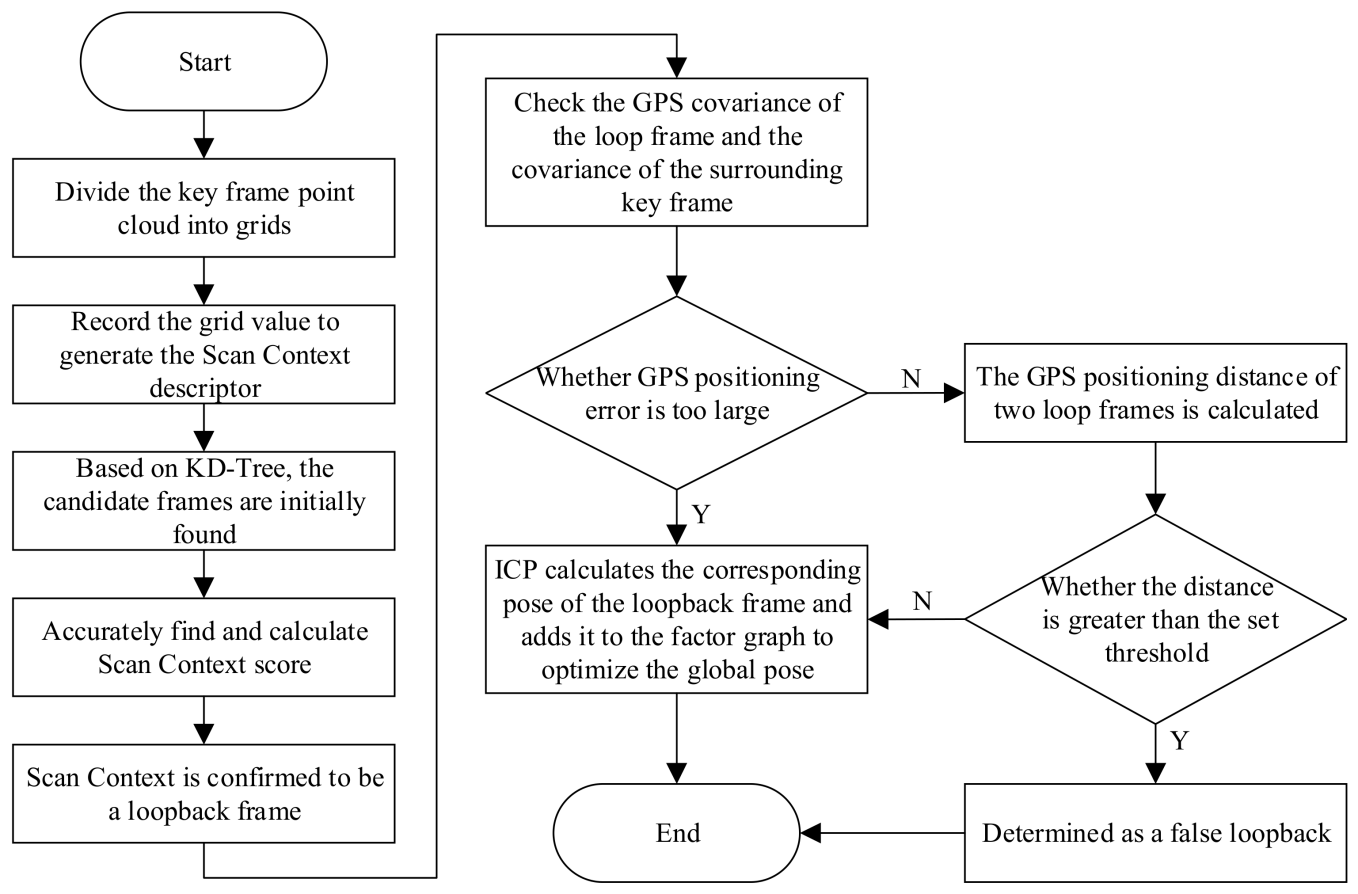
Complete loop closure detection flow.

In order to improve the above problems, this paper introduces GPS detection as a loop closure constraint on the basis of the original algorithm to determine whether a false loop closure occurs. The specific steps are as follows: The information of the original GPS latitude and longitude converted to UTM coordinates is added to the current keyframe and history keyframe, and the covariance matrix of the original GPS positioning result is retained. This covariance matrix can be used to determine whether there is drift in GPS positioning. When a loop closure frame is detected, i.e., when the Scan Context score is high at this time, the consistency and stability of the GPS covariance information matrix attached to the current frame and its first three frames are checked. The covariance threshold σ is used to determine whether the GPS positioning has drifted extensively. In the same way, the history loop closure frame is checked for large-scale drift and the GPS positioning distance between the two frames is calculated τ. If the distance exceeds 20 m, it is determined that the current is not the correct loop closure edge. The specific flow of the entire loop closure detection is shown in Figure 13.

### 4.3. Factor Graph Optimization Design

**Figure 14:**
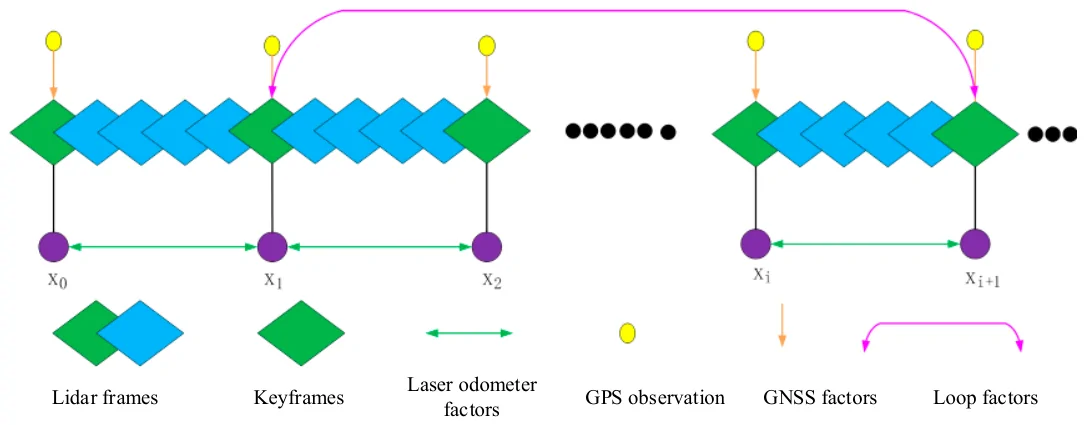
Factor graph optimization.

The factor graph back-end optimization fully considers all historical states and exploits the sparsity of the Jacobi matrix to improve the computational efficiency. In this paper, we refer to the GTSAM factor graph library to design the factor graph optimization model, as shown in Figure 14.

In SLAM, $x_{i}$ denotes the set of keyframe poses of the unmanned vehicle at a certain continuous time; the laser odometry factor is the relative pose between two keyframes; the loop closure factor is the relative pose between two loop closure frames obtained by loop closure detection; and the GPS factor is the GPS observation at a certain keyframe moment. The factor map optimization is performed once for each added LiDAR odometer keyframe pose, and the optimized result is used for the keyframe pose update. A global optimization is performed for each loop closure factor added, and all keyframe poses are updated. By fusing multiple sensor data, the whole SLAM system is made more robust.

## 5. Experimental Results and Analyses

### 5.1. Experimental Platforms and Evaluation Indicators

**Figure 15:**
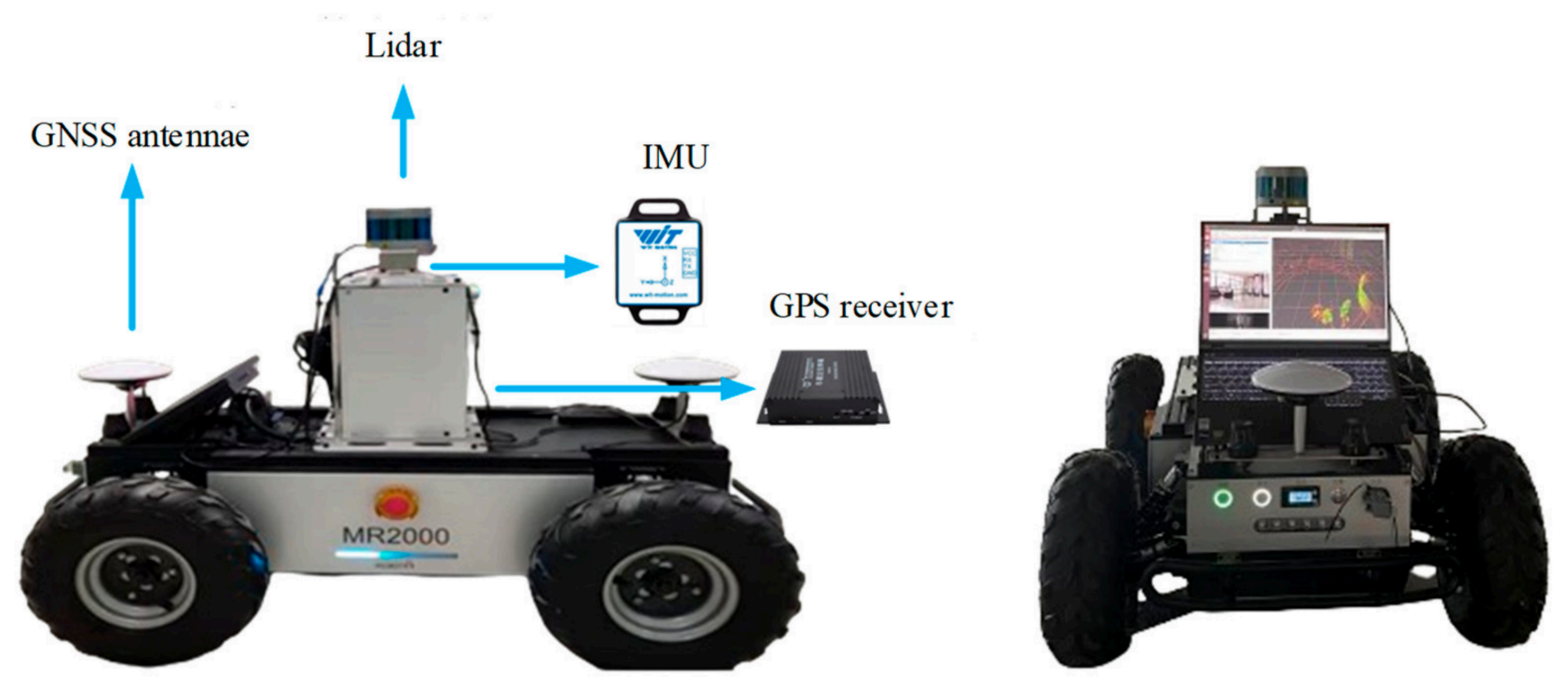
Unmanned vehicle experimental platform.

The unmanned vehicle experimental data acquisition platform used in this paper is equipped with a multifunctional cross-country unmanned vehicle development chassis model MR2000, which has strong obstacle-crossing ability and excellent control performance to ensure that the unmanned vehicle experimental platform operates freely in a variety of complex environments both indoors and outdoors. In this paper, the data acquisition and the computing platform used for the later verification of the algorithm is an industrial computer with superior stability, the computer CPU model i7-8700, with 6 cores and 12 threads, the maximum RWI of 4.6 GHz, running memory 16 G DDR4 frequency 2666 MHz, hard disk 1 T SSD, running Ubuntu 18.04 operating system, the development environment ROS Melodic, and the IDE is Vscode; the development environment ROS-X Melodic, IDE is VScode. The complete unmanned vehicle experimental platform is shown in Figure 15.

**Figure 16:**
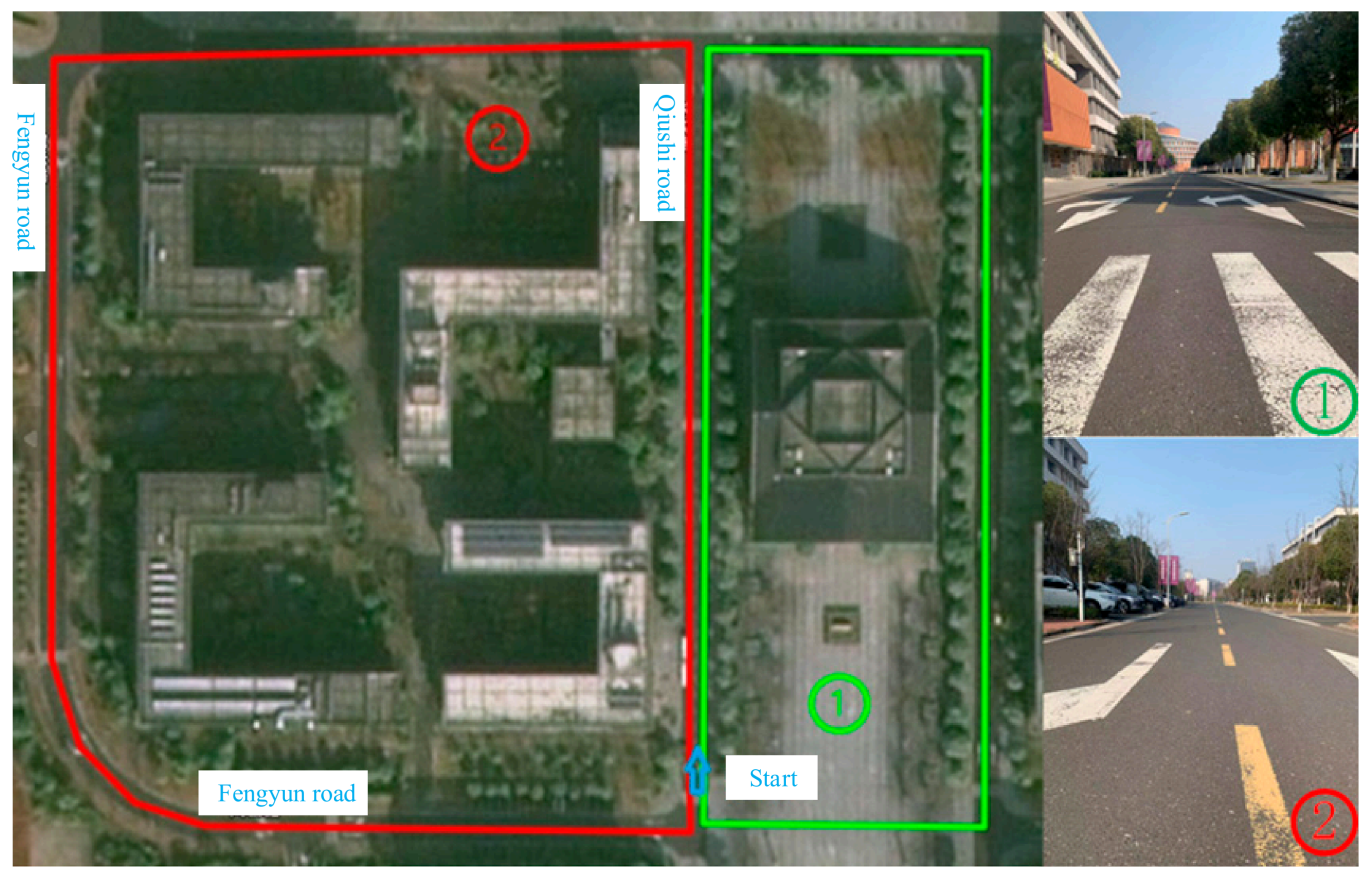
Satellite map of the campus laboratory site.

In order to verify the improvement effect of the method in this paper, two campus scene datasets are collected for algorithm validation using this unmanned vehicle platform, and both dataset roads have loop closure information and have no obvious gradient, and the maximum elevation error is less than 1 m. The satellite map of the campus dataset is shown in Figure 16.

The evaluation is conducted on two categories of datasets: (1) the public KITTI odometry benchmark [31] and (2) a self-collected campus dataset. For the KITTI dataset, we select four representative sequences from the structured urban category: Sequence 00 (4.5 km, urban with loops), Sequence 05 (2.2 km, urban with moderate traffic), Sequence 07 (0.7 km, urban residential), and Sequence 08 (3.2 km, urban with highway). In the results tables, these are labeled as sscene 1 through Scene 4, respectively. The campus dataset is collected using a Clearpath Husky UGV equipped with a RoboSense 16-line LiDAR (10 Hz), a MicroStrain 3DM-GX5-25 IMU (200 Hz), and a u-blox ZED-F9P RTK-GPS module (10 Hz). The sensor suite is extrinsically calibrated using the method of [17]. The campus dataset consists of two sequences: campus 1 (1.2 km, structured campus roads with buildings and trees) and campus 2 (1.8 km, mixed campus and parking lot environments). For each dataset, three independent runs are performed to assess statistical significance, and the mean and standard deviation of all metrics are reported.

In order to objectively assess the effectiveness of the algorithms designed in this paper, the SLAM algorithm effectiveness is verified using the urban scene dataset sequences KITTI_00, KITTI_05, KITTI_07, and KITTI_08 from the publicly available KITTI dataset.

In order to verify the accuracy and robustness of the algorithm designed in this paper, we evaluate it against current mainstream LiDAR SLAM algorithms using the open-source tool evo. Absolute Trajectory Error (ATE) and Relative Pose Error (RPE) of the current trajectory and reference trajectory are evaluated. Absolute Trajectory Error is used to evaluate the positioning accuracy of the algorithm, and Relative Pose Error is used to evaluate the accuracy of the algorithm’s pose estimation.

### 5.2. Experimental Results and Analysis of Raster-Based Feature Extraction

**Figure 17:**
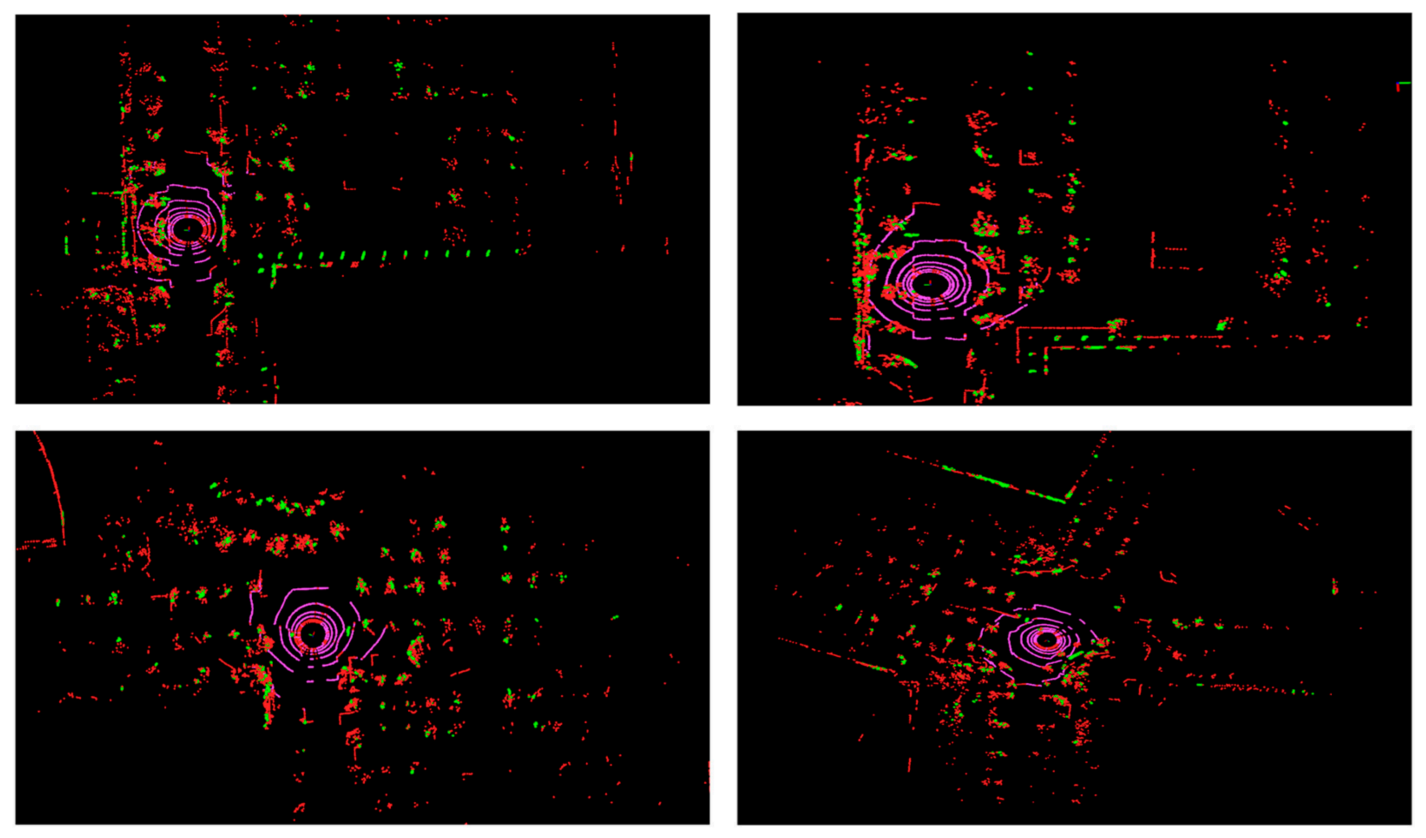
16-line LiDAR feature extraction effect.

In order to solve the problem of front-end odometer elevation accumulation error, this paper aims at the characteristics of structured urban scenes and proposes a raster-based feature extraction method, which is able to extract line features, surface features and ground points at the same time. Figure 17 shows the campus dataset 16-line LiDAR with the effect of feature extraction using the method proposed in this paper.

**Figure 18:**
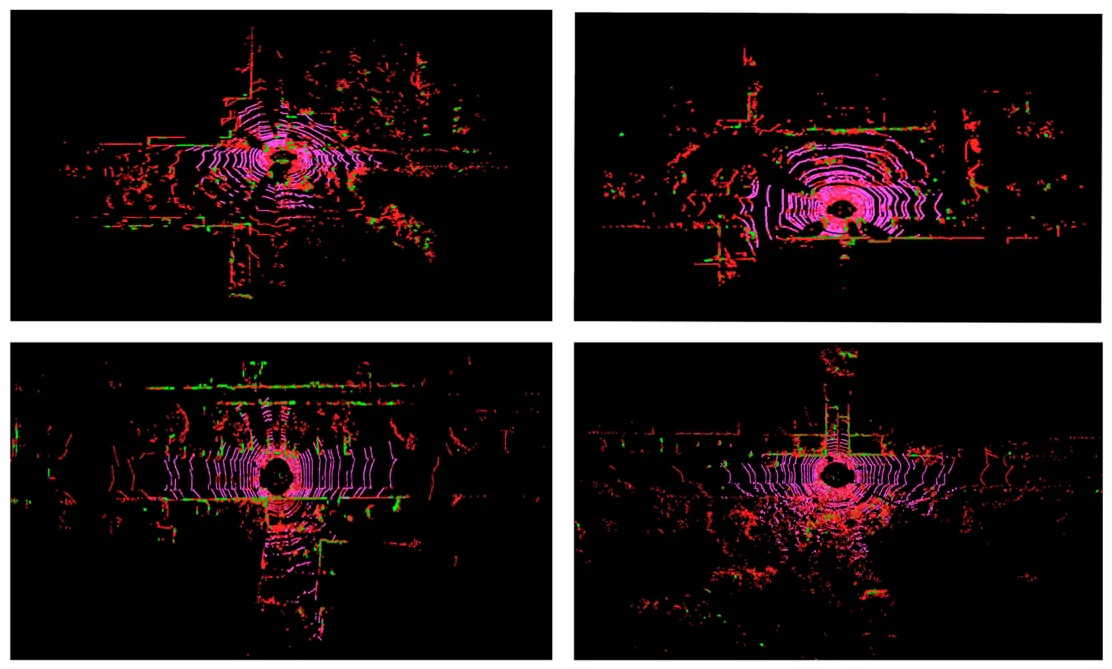
64-line LiDAR feature extraction effect.

Figure 18 shows the KITTI dataset 64-line LiDAR with the effect of point cloud feature extraction using the method of this paper. Through the campus dataset and public KITTI’s dataset test, the feature extraction strategy designed in this paper can reliably complete the feature extraction work in structured scenarios. In the figure, green is the line feature, red is the surface feature, and pink is the extracted ground point; the feature point distribution is uniform, and the extraction effect is stable.

**Figure 19:**
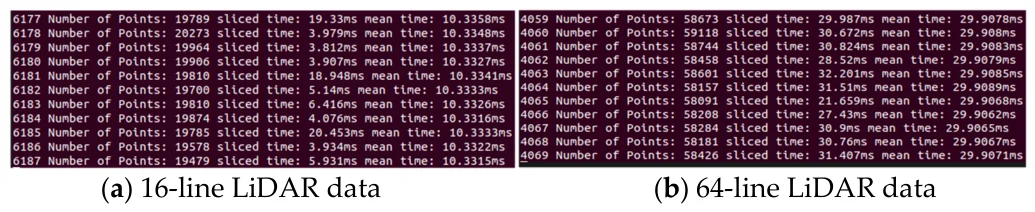
Feature extraction time test.

The proposed feature extraction method adopts a raster-based segmentation strategy that fully leverages the ring information inherent in LiDAR point clouds. Each raster is processed independently using multi-threaded acceleration. After obtaining the reference point for each raster, slope correlation is computed instead of the traditional curvature-based approach. As shown in Figure 19, we tested 6187 frames of 16-line LiDAR data on an i7-8700 platform, with an average of 19,500 points per frame and an average feature extraction time of 10.33 ms. For the KITTI_08 dataset, we tested 4069 frames of 64-line LiDAR data, with an average of 58,400 points per frame and an average feature extraction time of 29.91 ms.

The detailed data of feature extraction time consumption of this paper’s method is shown in Table 2. The above experiments show that the feature extraction method in this paper consumes time to meet the real-time requirements of the SLAM front-end.

**Figure 20:**
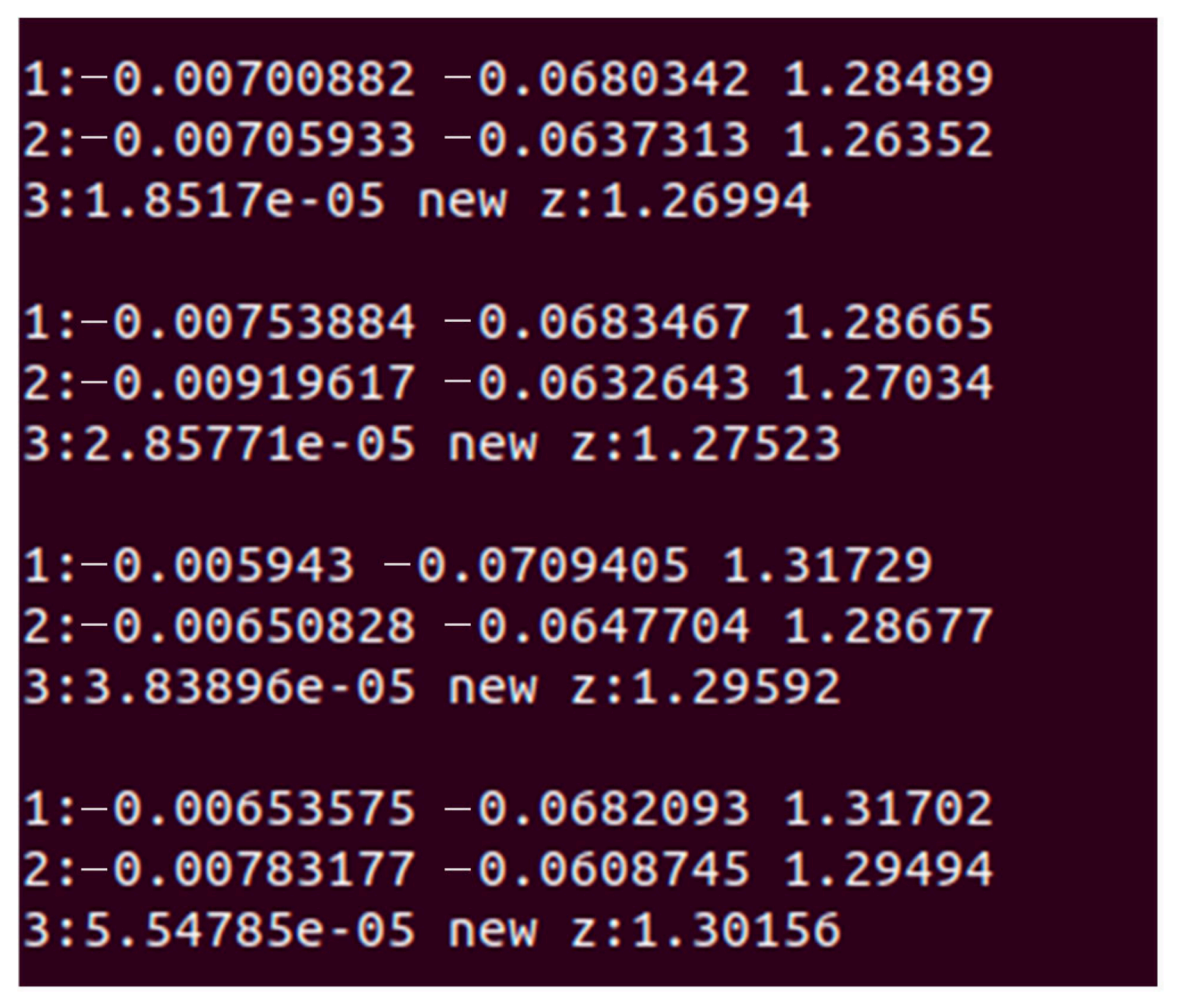
Interpolation of elevation weights.

The cumulative elevation error of the front end of the laser odometer affects the final map-building effect. In this paper, after extracting the ground points from a frame of point cloud, the method of constructing ground constraints is used to reduce the cumulative elevation error. And the interpolation weight is determined by measuring the roll angle and pitch angle after alignment, and the interpolation process is shown in Figure 20.

**Figure 21:**
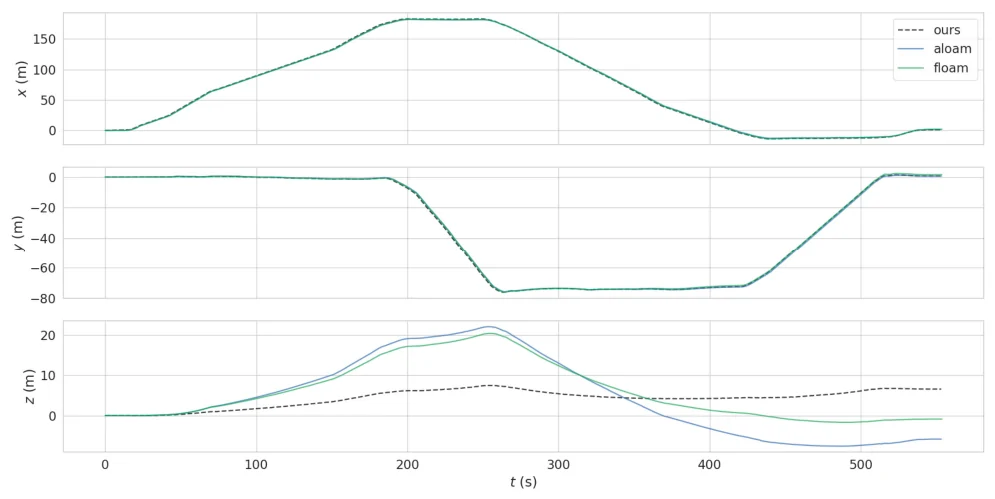
Campus dataset 1 XYZ errors.

**Figure 22:**
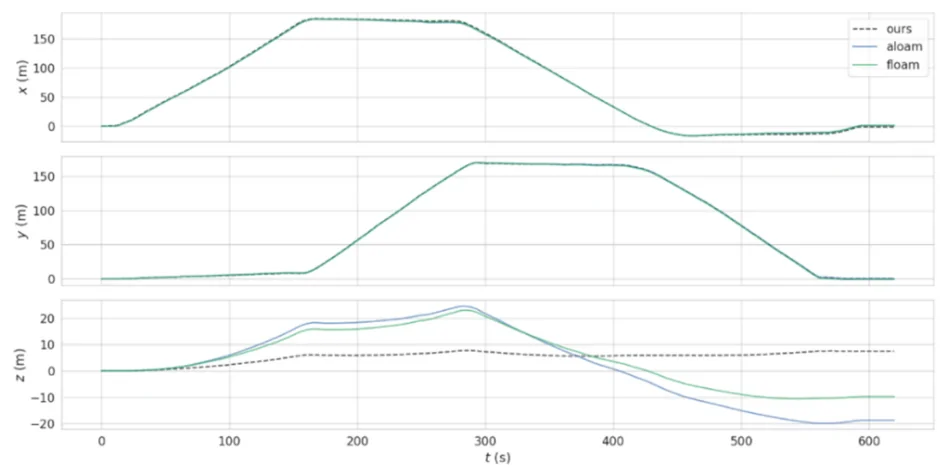
Campus dataset 2 XYZ error.

Figure 1 above represents the roll, pitch, and z of the line feature and surface feature alignment results, where roll and pitch are in radians and z is in meters. Figure 2 above represents roll, pitch, and z of the ground point alignment results. The front part of Figure 3 in the above figure represents the squared difference between the two alignment results, roll and pitch, and the final part shows the z after the interpolation update. The purpose of reducing the cumulative front-end elevation error is achieved by constraining z for each frame. Figure 21 and Figure 22 present the evo evaluations of our front-end laser odometry against the mainstream laser odometers ALOAM and FLOAM on the campus dataset. ALOAM follows the way of LOAM using high- and low-frequency methods to realize laser odometry. FLOAM uses the current frame to local map (Scan-to-Map) alignment method, which is basically the same as the alignment method used in this paper but with a different way of feature point extraction. In this paper, the campus dataset was collected in an environment with a flat road surface, no significant slope, and a maximum elevation error of less than 1 m.

Figure 21 shows the elevation errors for ALOAM, FLOAM, and the laser odometer in this paper after running campus Dataset 1 for approximately 550 m. The maximum elevation error of ALOAM and FLOAM is more than 20 m, and the front-end odometer designed in this paper is the dashed part; the maximum elevation error is less than 9 m and can be stabilized near the origin at the end. Overall, the trend of elevation error is smooth, and the method designed in this paper has a constraining effect on the elevation.

Figure 22 shows the elevation error after running campus dataset 2 for approximately 700 m. The elevation error, although not eliminated, is significantly smaller than the elevation errors of ALOAM and LOAM. After many subsequent tests, it is found that the number of ground points of 16-line LiDAR accounts for a relatively small number of points, and the measurement error of LiDAR itself is also a major source of cumulative error; the method of this paper will be more effective in the performance of LiDAR with more ground points and more lines. The above experimental results show that the method in this paper is effective, using the construction of ground constraints for each frame to achieve the purpose of reducing the global elevation cumulative error.

**Figure 23:**
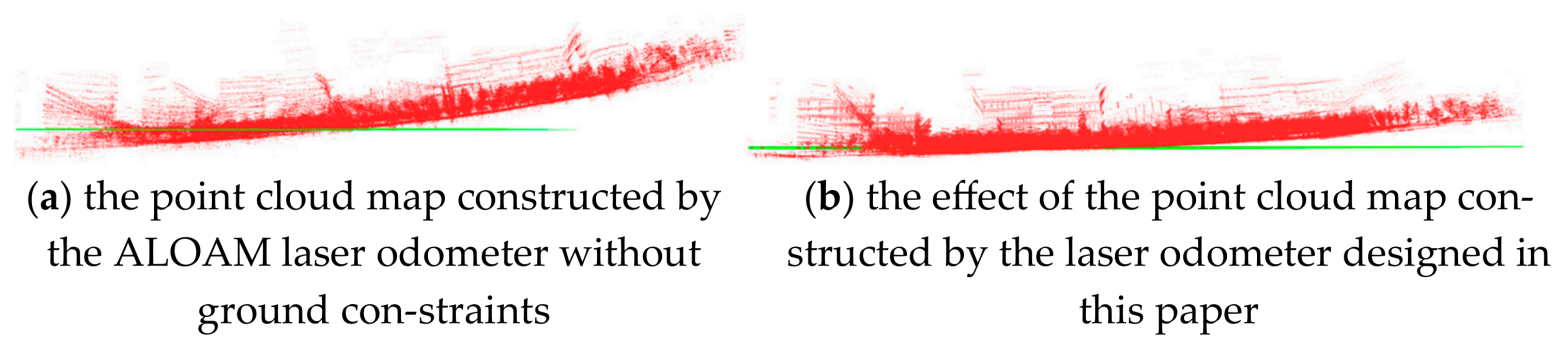
Schematic diagram of elevation cumulative error reduction. (**a**) the point cloud map constructed by the ALOAM laser odometer without ground con-straints; (**b**) the effect of the point cloud map constructed by the laser odometer designed in this paper.

Figure 23 shows the side view effect of the ALOAM laser odometer and the laser odometer designed herein to construct the map. Figure 23a shows the point cloud map constructed by the ALOAM laser odometer without ground constraints, and Figure 23b shows the effect of the point cloud map constructed by the laser odometer designed in this paper. Although the elevation cumulative error is not completely eliminated, the method designed in this paper acts as a constraint on the elevation error, and the cumulative error does not grow linearly. The method in this paper effectively reduces the front-end laser odometer elevation cumulative error while ensuring stable system operation.

### 5.3. Results and Analysis of Loop Closure Detection Incorporating GPS Constraints

**Figure 24:**
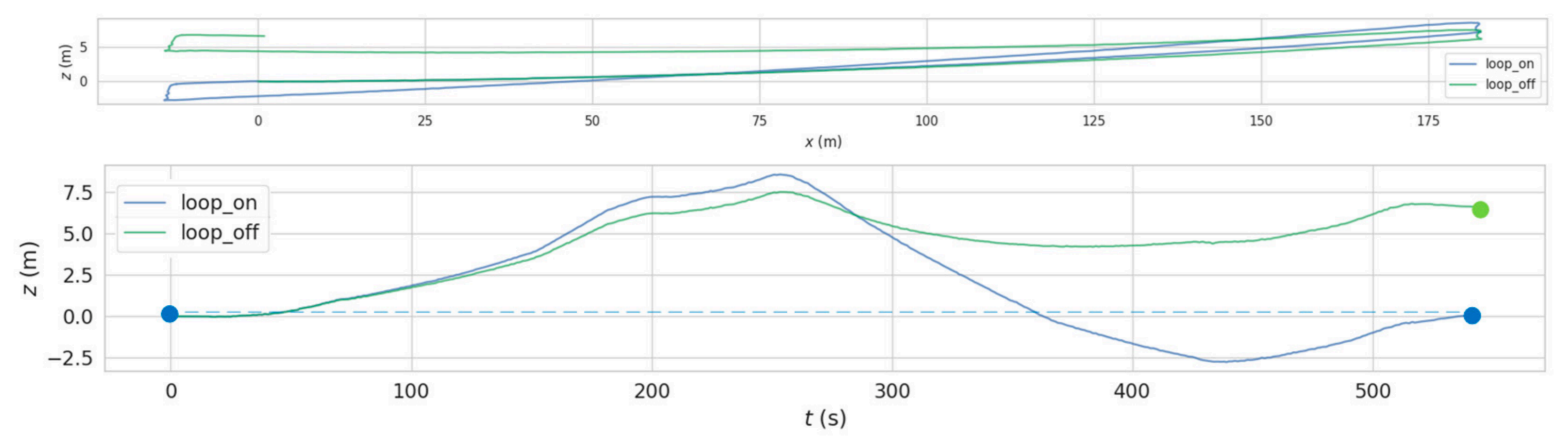
Comparison of loop closure detection trajectories.

In order to verify the effectiveness of the loop closure detection module at the back-end of the SLAM algorithm designed in this paper, the accuracy of the localization trajectories was tested with and without loop closures, respectively. The campus dataset was first tested, and the loop closure detection trajectory is shown in Figure 24. The green line is the front-end laser odometer localization trajectory in the case of no loop closure detection, with an elevation error of about 7 m from the starting point, and does not exhibit a convergence trend. The blue color shows the unmanned vehicle positioning trajectory after loop closure detection is added, and the cumulative error is corrected after the loop closure is detected, and the positioning trajectory converges back to the starting position, which is in line with the actual situation.

**Figure 25:**
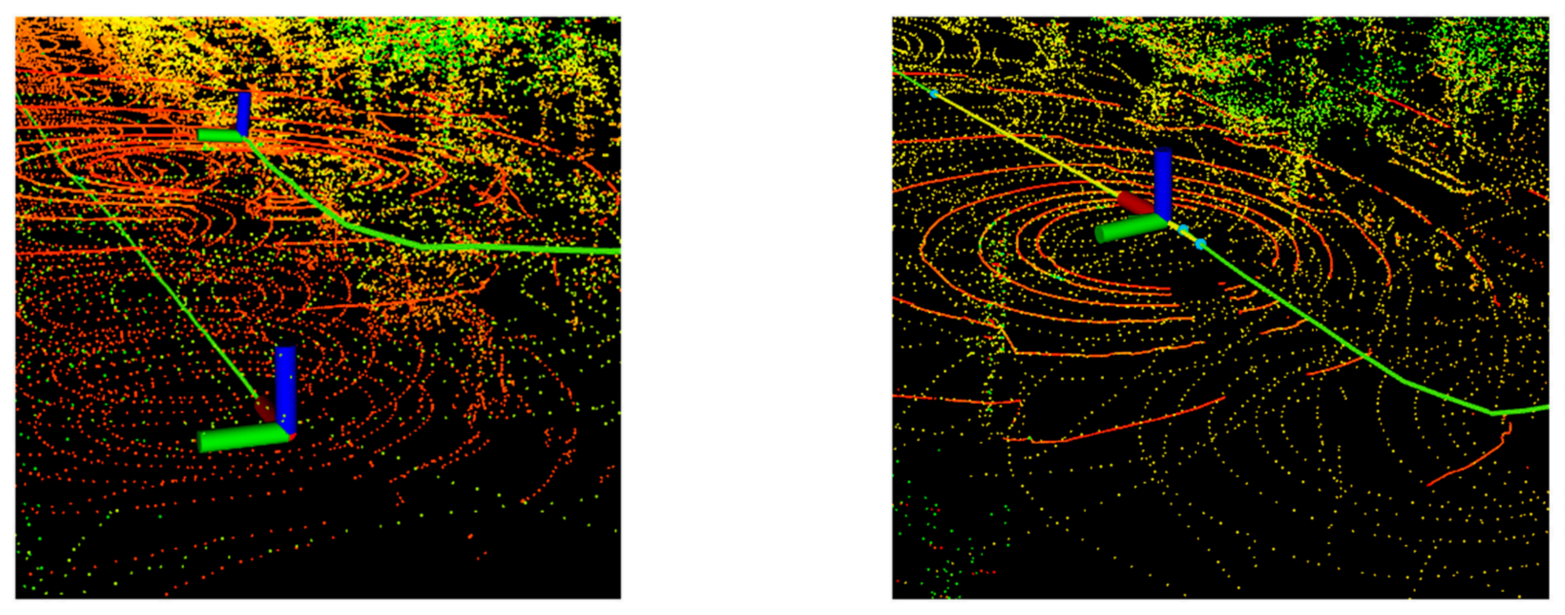
Loop closure detection eliminates cumulative error.

The left side of Figure 25 illustrates the localization trajectory without loop closure detection, with a significant cumulative error. With the introduction of loop closure detection, the cumulative error gradually decreases and eventually converges to the unmanned vehicle departure position.

**Figure 26:**
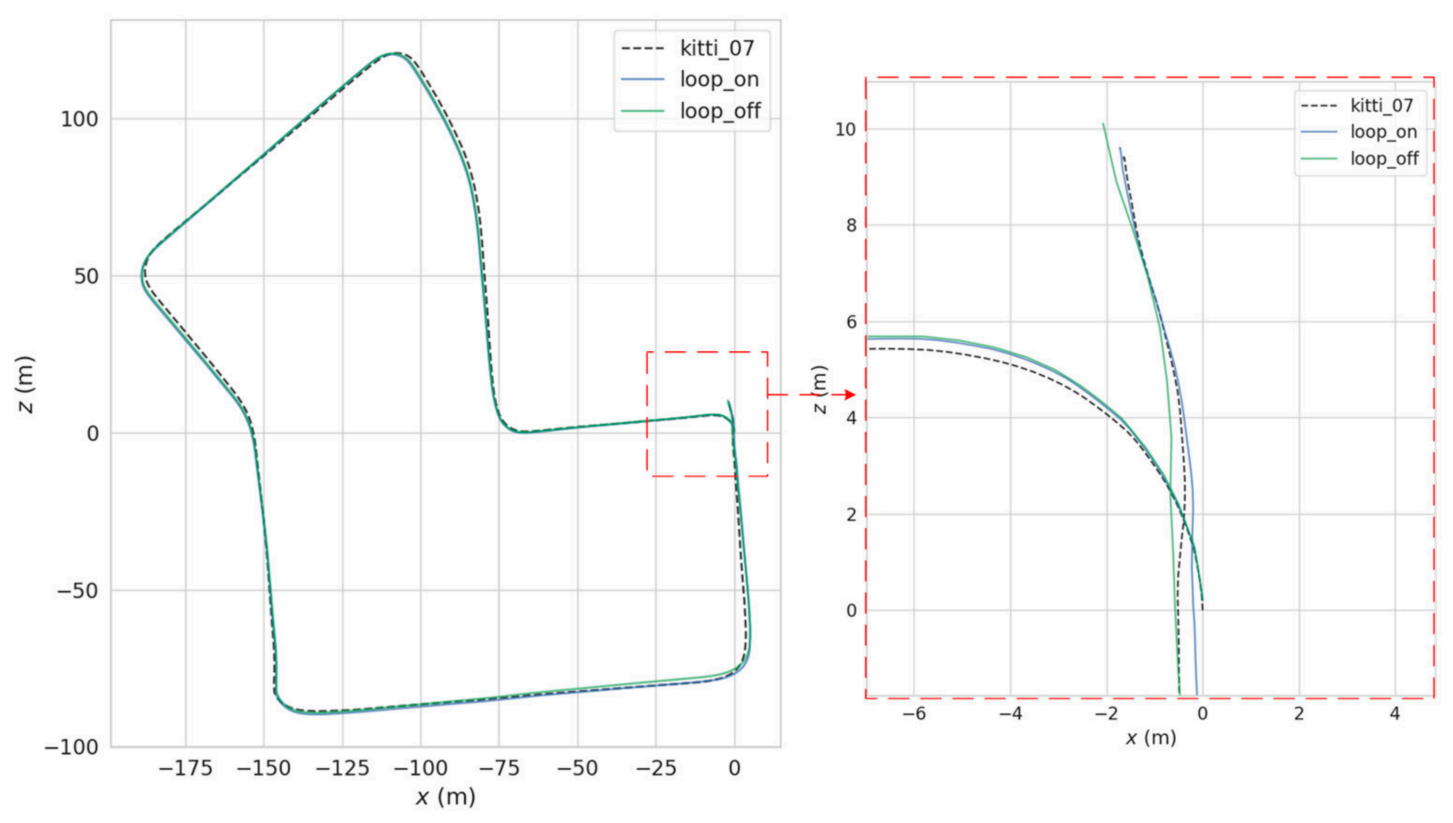
KITTI_07 with and without loop closure global trajectory.

**Figure 27:**
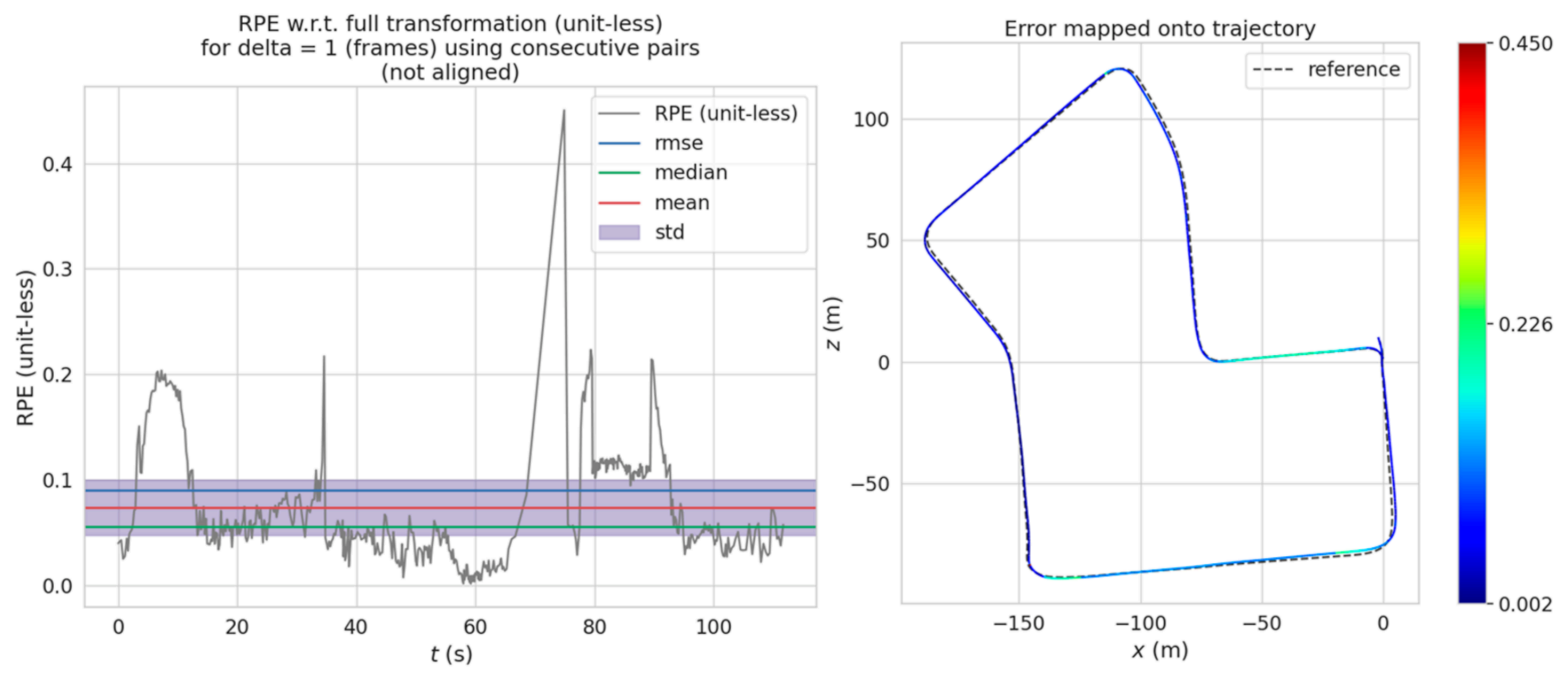
KITTI_07 without loop closure detection RPE.

**Figure 28:**
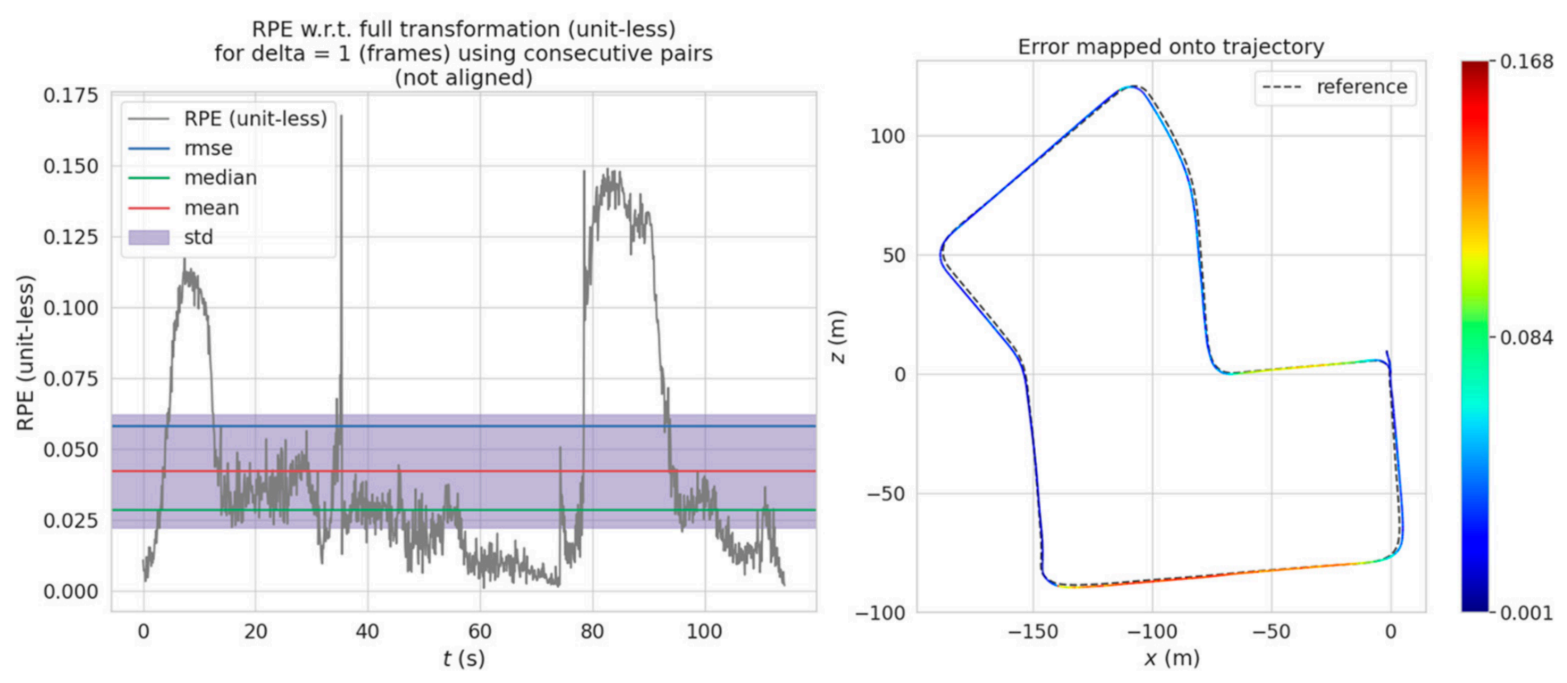
KITTI_07 with loop closure detection RPE.

In this paper, we test the effectiveness of the loop closure checking module of this paper on the KITTI_07 dataset, and the global trajectory pairs with and without loop closure detection are shown in Figure 26. The RPE evaluations of the localization trajectory and the truth trajectory are shown in Figure 27 and Figure 28. The left side of the figure shows the RPE trend curves for the localization trajectory and the true value trajectory, with the blue, green, and red lines representing the root mean square, median, and mean of the RPE, respectively. On the right side of the figure is the global trajectory drawn according to the RPE size. The closer the RPE is to the global RPE maximum, the redder the trajectory color is; conversely, the closer it is to blue. The trajectory after adding loop closure detection is closer to the real value.

Table 3 shows the detailed data of the RPE evaluation of the localization trajectory and the true value trajectory for this paper’s algorithm in testing the KITTI_07 dataset with and without the loop closure detection module, and the RPE evaluation interval is 1 m. Compared with without loop closure detection, this paper’s algorithm reduces the maximum value of the RPE of the localization trajectory from 0.45 to 0.17, the mean value of the RPE is reduced by 42.76%, the standard deviation is reduced by 24.29%, and the root-mean-square RPE is reduced by 35.90% with the addition of the loop closure detection module, which is a significant improvement in all the error metrics.

**Figure 29:**
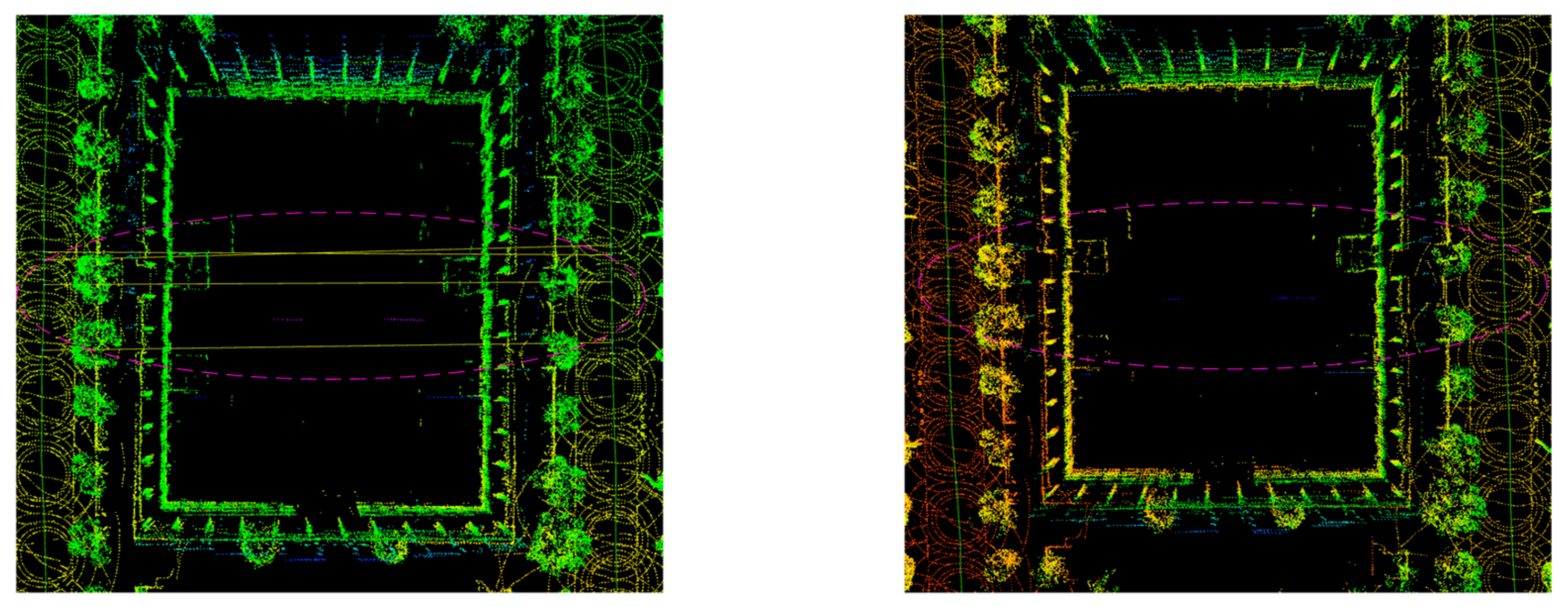
Scan Context loop closure detection optimization results.

In order to solve the problem of false loop closure of the Scan Context method in structurally similar environments, this paper improves the accuracy of loop closure detection by adding GPS to loop closure detection. The optimization results of loop closure detection for campus dataset 1 are shown in Figure 29. The connected portion of the left figure indicates the cases in the dataset that were mistakenly identified as loop closures by Scan Context due to the similarity of the scenes, and the right figure demonstrates the results of the inclusion of GPS-assisted discrimination, where the false loop closures were successfully identified and rejected.

**Figure 30:**
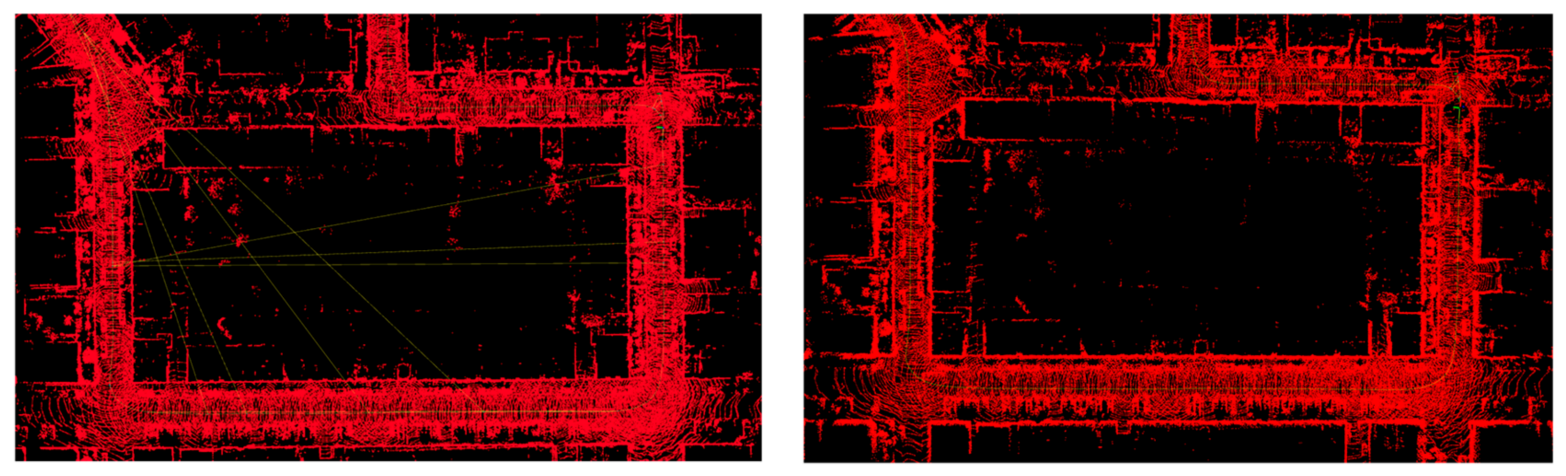
Schematic diagram of false loop closure discrimination.

The loop closure detection optimization results for the KITTI_07 dataset are shown in Figure 30. The trajectory connecting lines in the left figure indicates the cases where the edges are misidentified as loop closures due to the similarity of the scenes. The right figure shows that after the introduction of GPS discrimination, these false loop closures are successfully identified and rejected, thus avoiding unnecessary alignment, reducing the consumption of computational resources and minimizing interference to the system.

Through the above validation and analysis, the effectiveness of the back-end loop closure detection module designed in this paper is proved to be able to reliably accomplish the loop closure detection task in structured urban scenarios, eliminating the cumulative errors generated by the robot during motion.

### 5.4. Algorithm Synthesis Comparison Experiment

In order to objectively evaluate the effectiveness of the algorithms designed in this paper, sequences 00, 05, 07, 08 of the publicly available KITTI dataset of structured urban scenes are used for validation, and the tightly coupled SLAM algorithm based on the combined inertial guidance of LiDAR designed in this paper is compared to the current mainstream open-source laser SLAM algorithms ALOAM, FLOAM, LeGO-LOAM, and LIO-SAM for a comparative analysis to systematically evaluate the performance of the method in this paper. Three tests are performed for each dataset to minimize the effect of chance errors.

**Figure 31:**
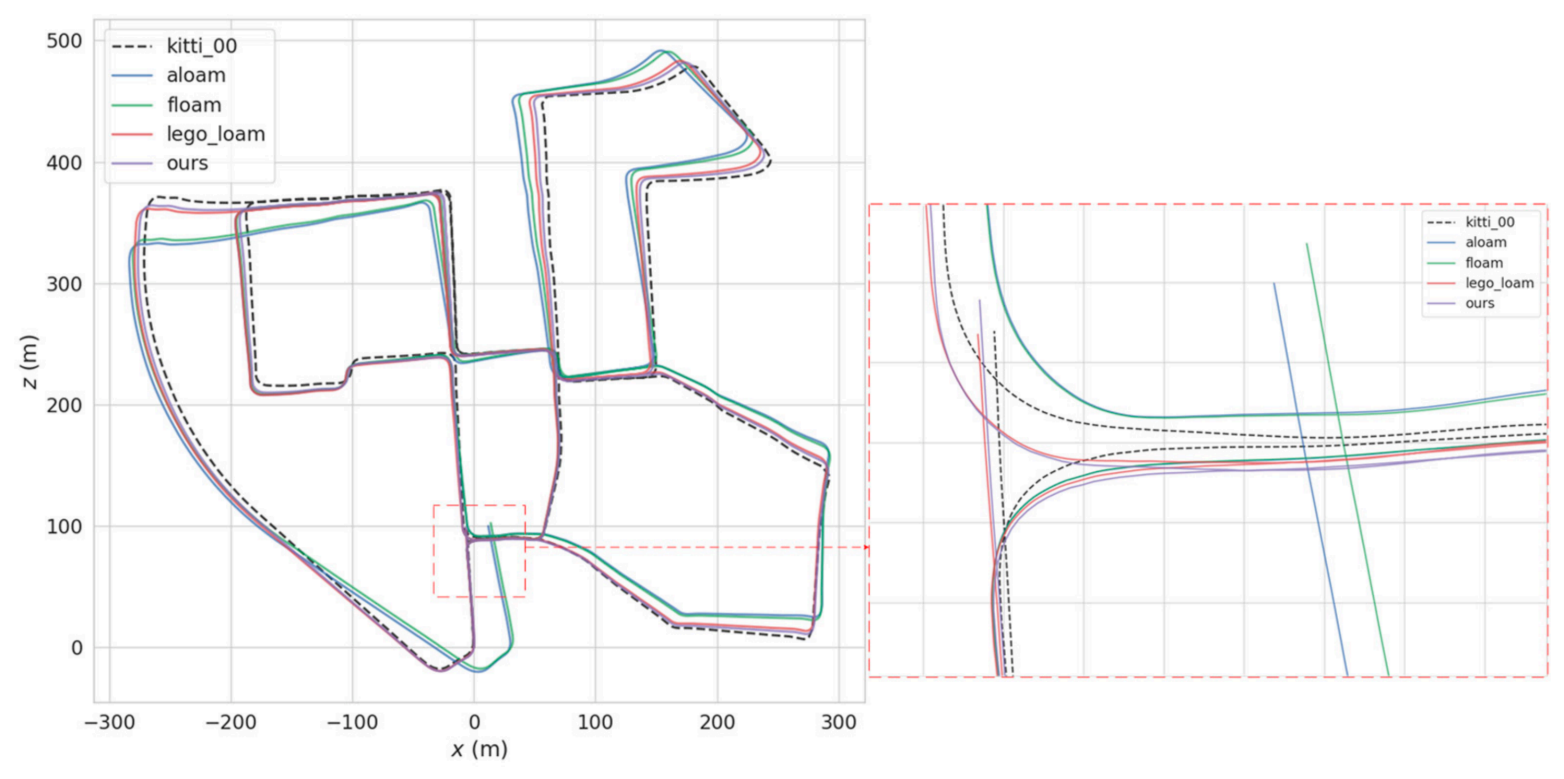
Comparison of global trajectories in scene 1.

**Figure 32:**
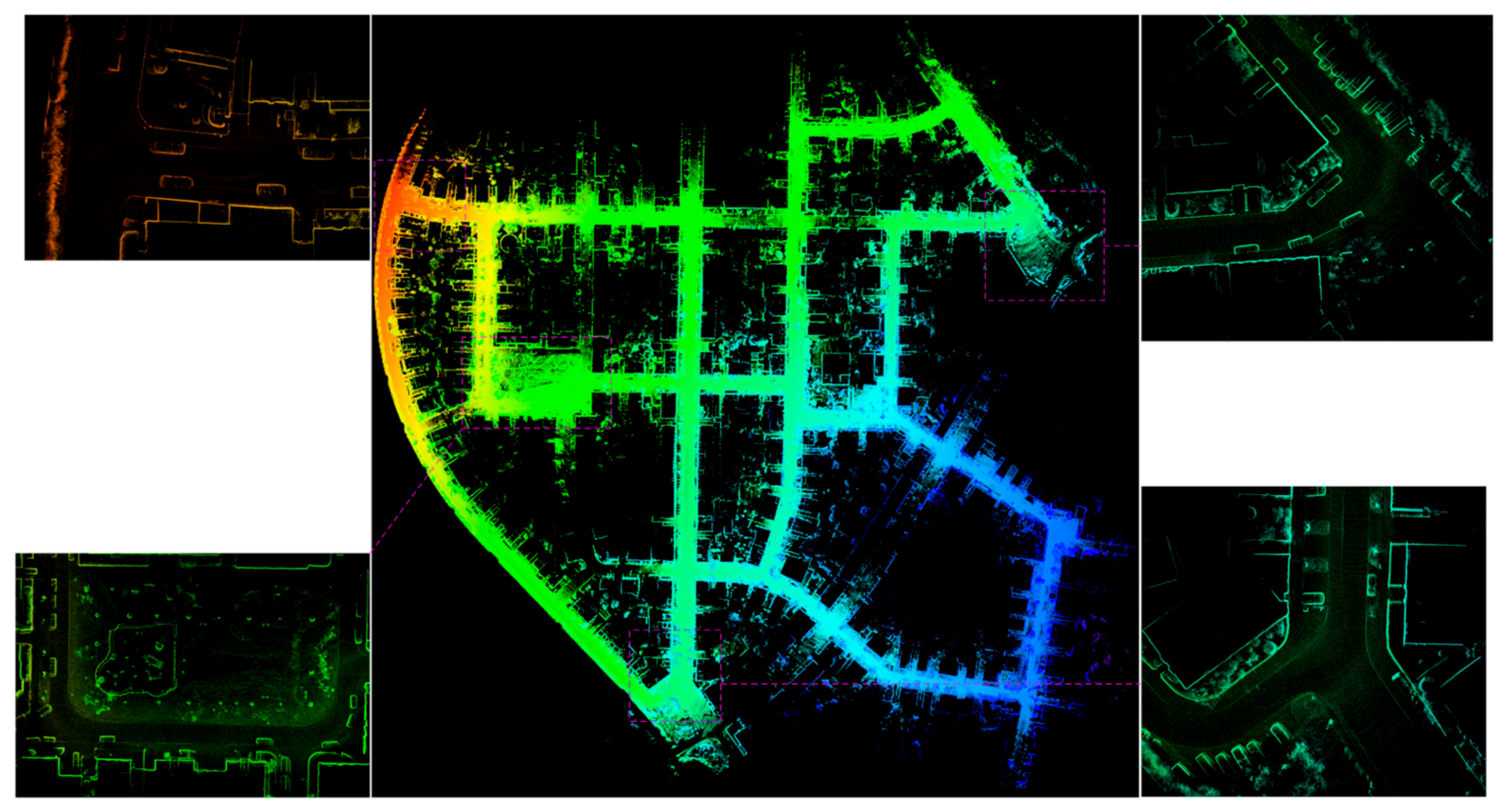
Scene 1 global point cloud map.

Scene 1 has a total length of 3714 m with multiple loop sections. The global trajectory drawn by the algorithm designed in this paper and the comparison algorithm under scene 1 are shown in Figure 31. The global point cloud map of scene 1 constructed by the algorithm of this paper is shown in Figure 32.

In scene 1, the algorithm designed in this paper compared with ALOAM, FLOAM, LeGO_LOAM each error value has been reduced to a certain extent, in terms of the root mean square APE, for example, reduced by 42.44%, 40.41%, 15.02%, respectively, and the detailed comparison of the parameters are shown in Table 4.

**Figure 33:**
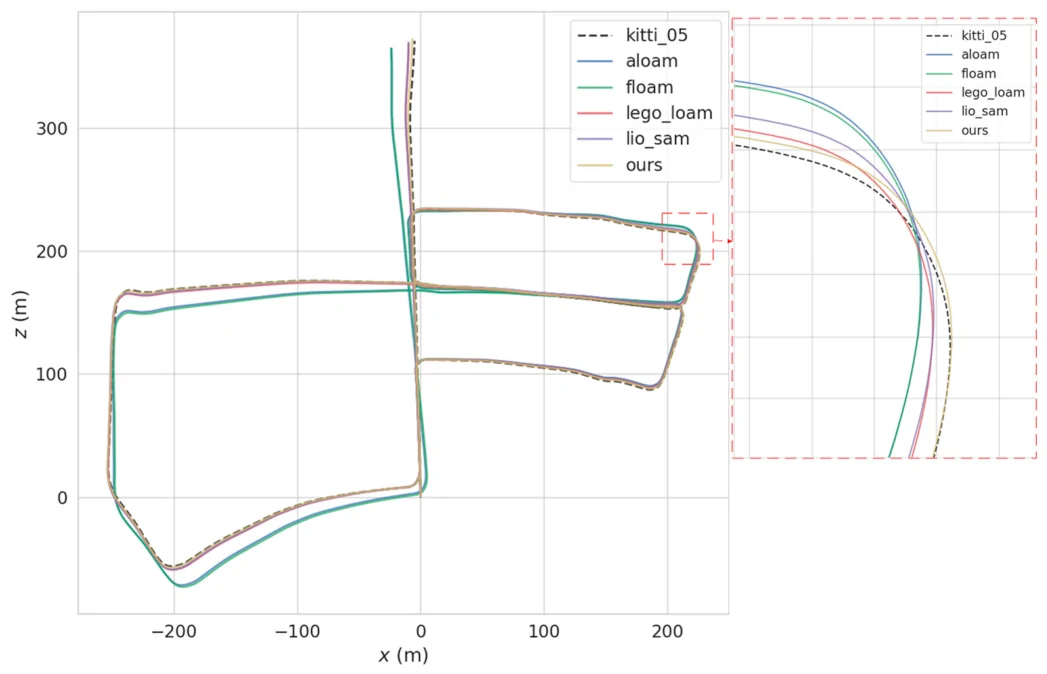
Comparison of trajectories in scene 2.

**Figure 34:**
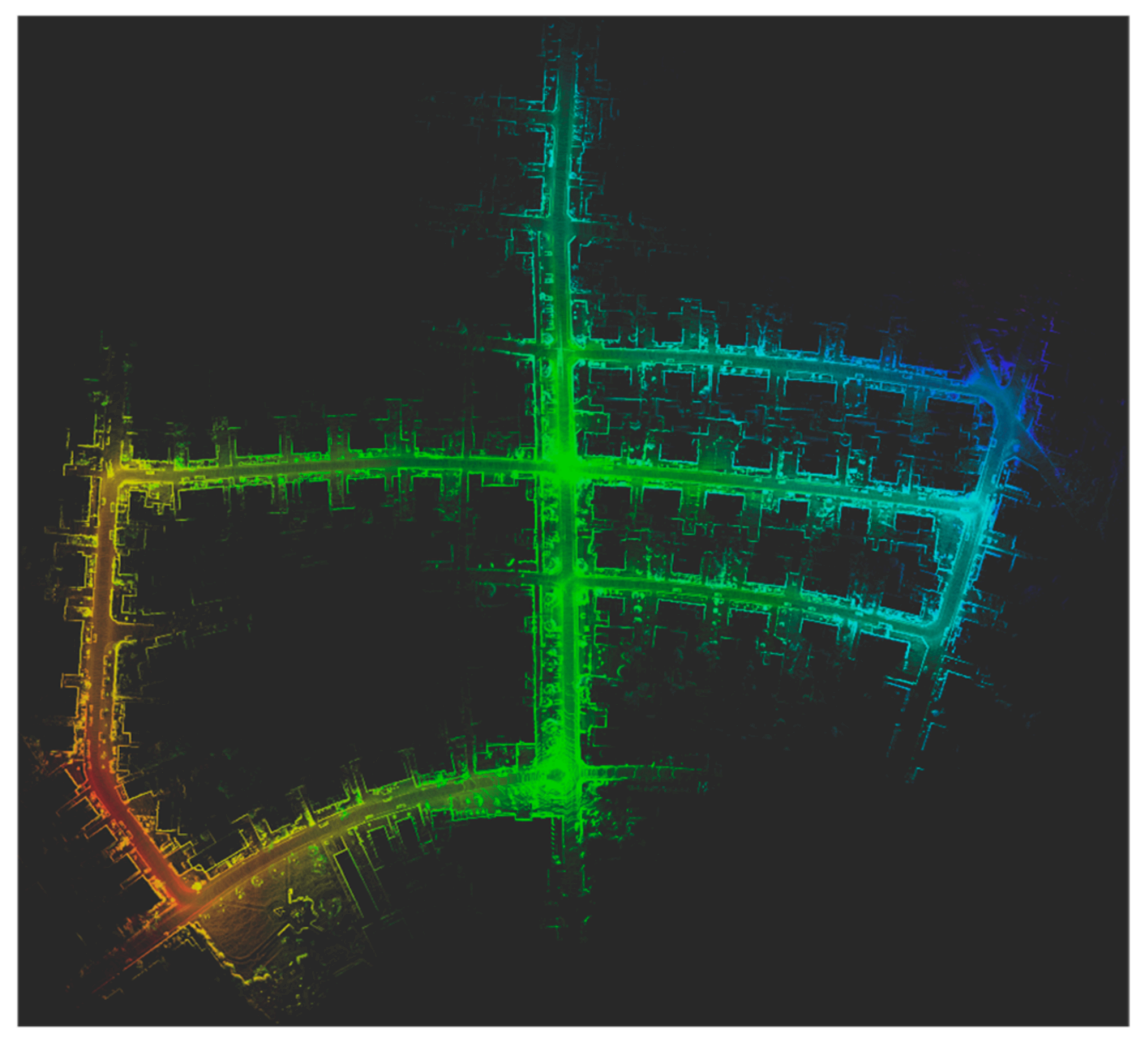
Scene 2 global point cloud map.

The total length of the road in scene 2 is 2223 m, and there are forward and reverse loops in scene 2 to comprehensively test the loop closure performance of the system. The global trajectory drawn by the algorithm designed in this paper and the comparison algorithm under scene 2 are shown in Figure 33. The global point cloud map of scene 2 constructed by the algorithm of this paper is shown in Figure 34.

In scene 2, the method of this paper reduces the root mean square APE by 36.45% and 25.72% compared to LeGO-LOAM and LIO-SAM algorithms, respectively, and the detailed comparison parameters are shown in Table 5.

**Figure 35:**
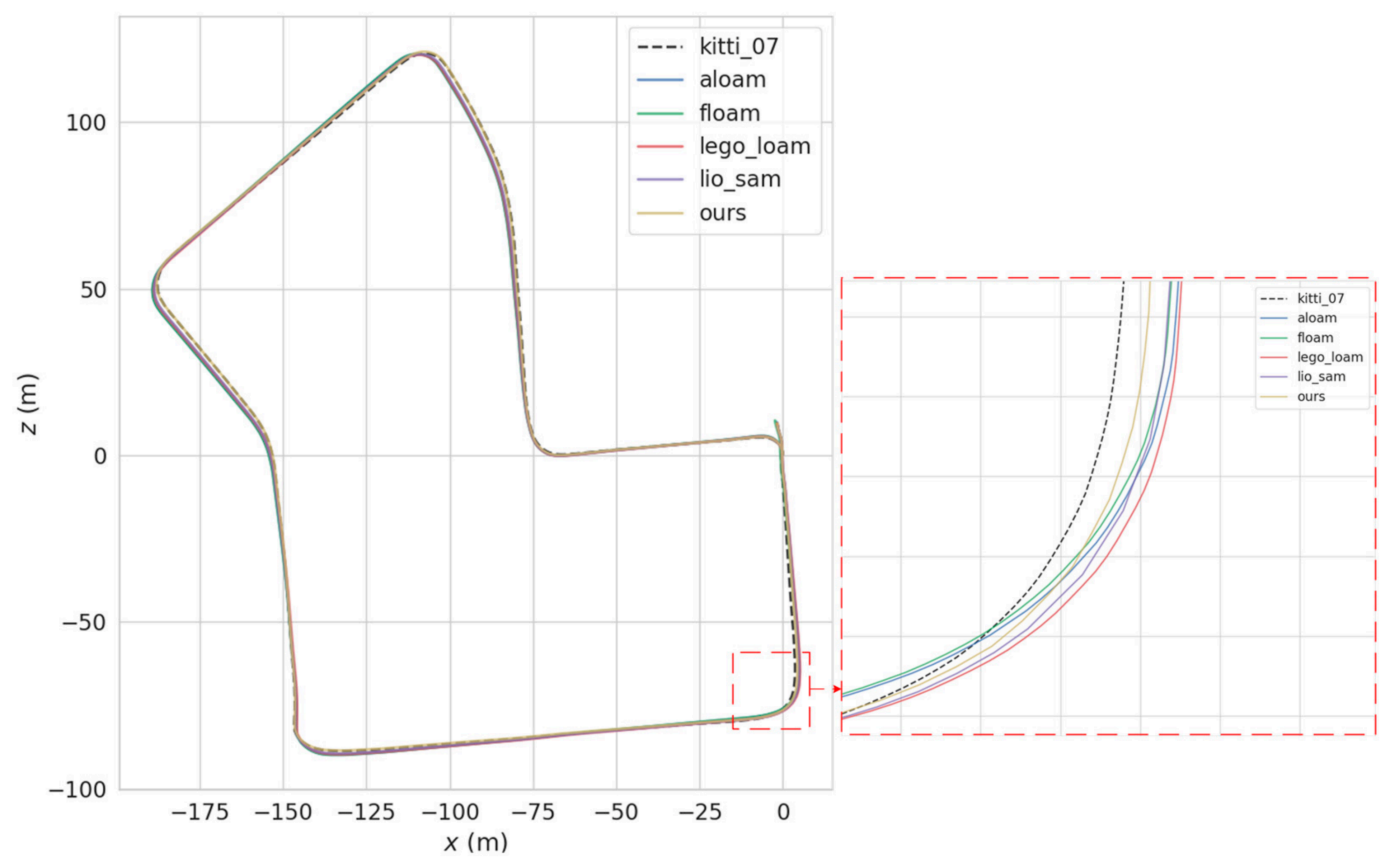
Comparison of trajectories in scene 3.

**Figure 36:**
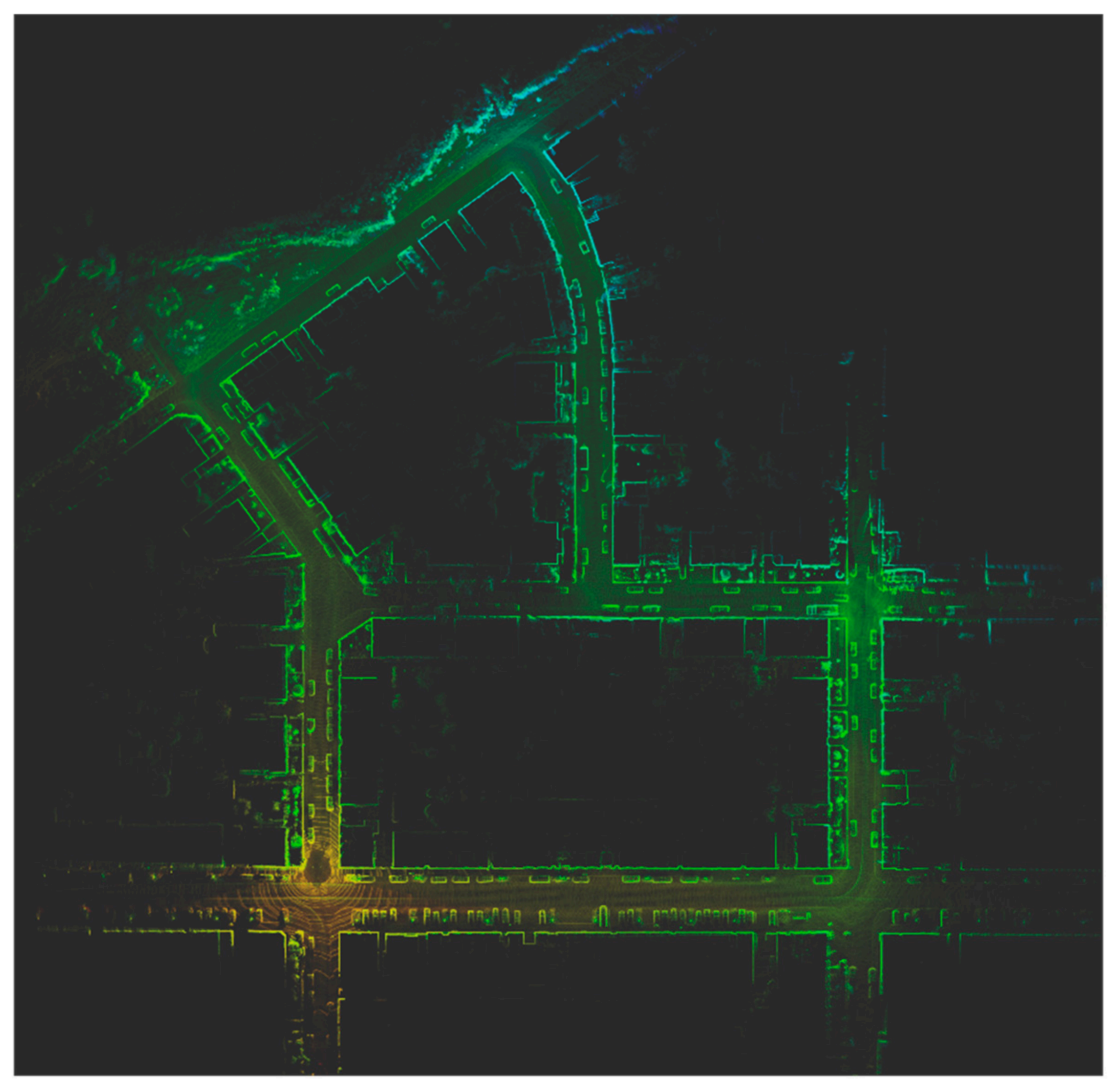
Scene 3 global point cloud map.

Scene 3 is an urban road with a total length of 695 m, and there exists a positive loop closure. The global trajectory drawn under scene 3 by the algorithm designed in this paper and the comparison algorithm is shown in Figure 35. The global point cloud map of scene 3 constructed by the algorithm of this paper is shown in Figure 36.

ALOAM and FLOAM perform better in this relatively simple scenario without large deviations. The root mean square APE of this paper’s algorithm is reduced by 20.00%, 26.67%, 18.45%, and 4.40%, respectively, compared to the comparison algorithms, and the specific comparison parameter comparisons are shown in Table 6.

**Figure 37:**
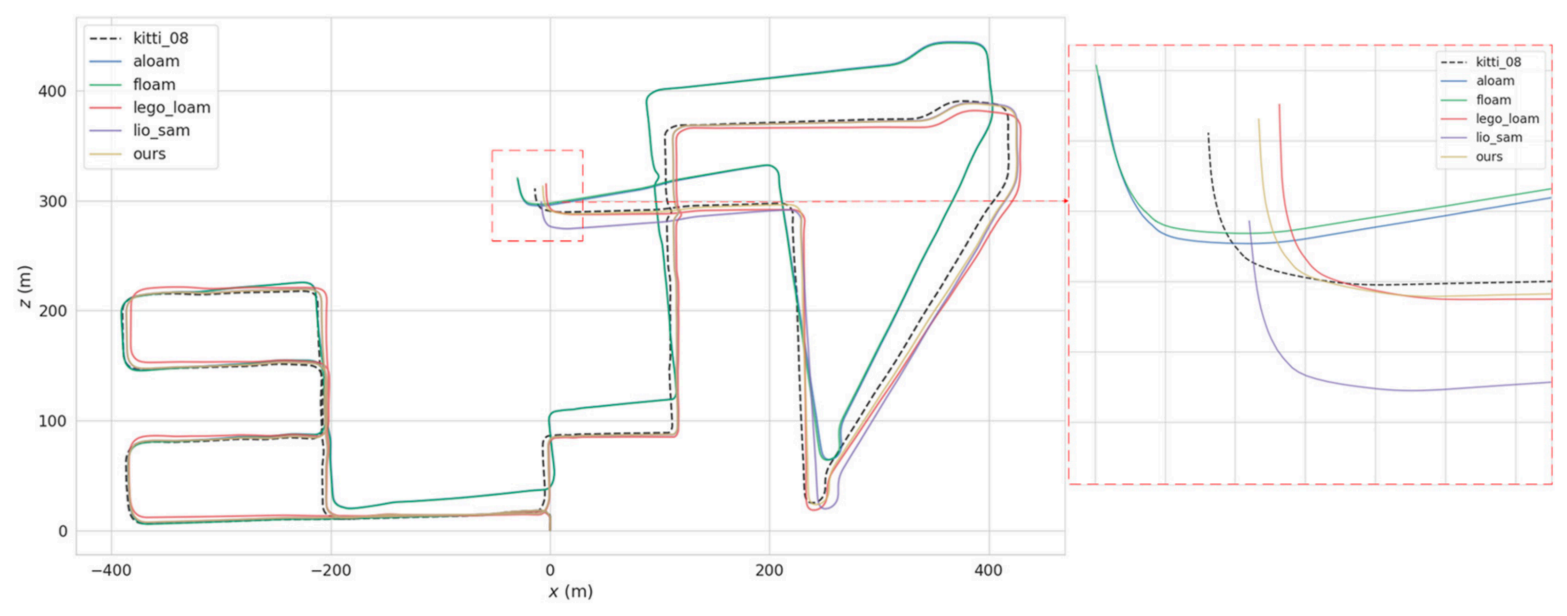
Comparison of trajectories in scene 4.

**Figure 38:**
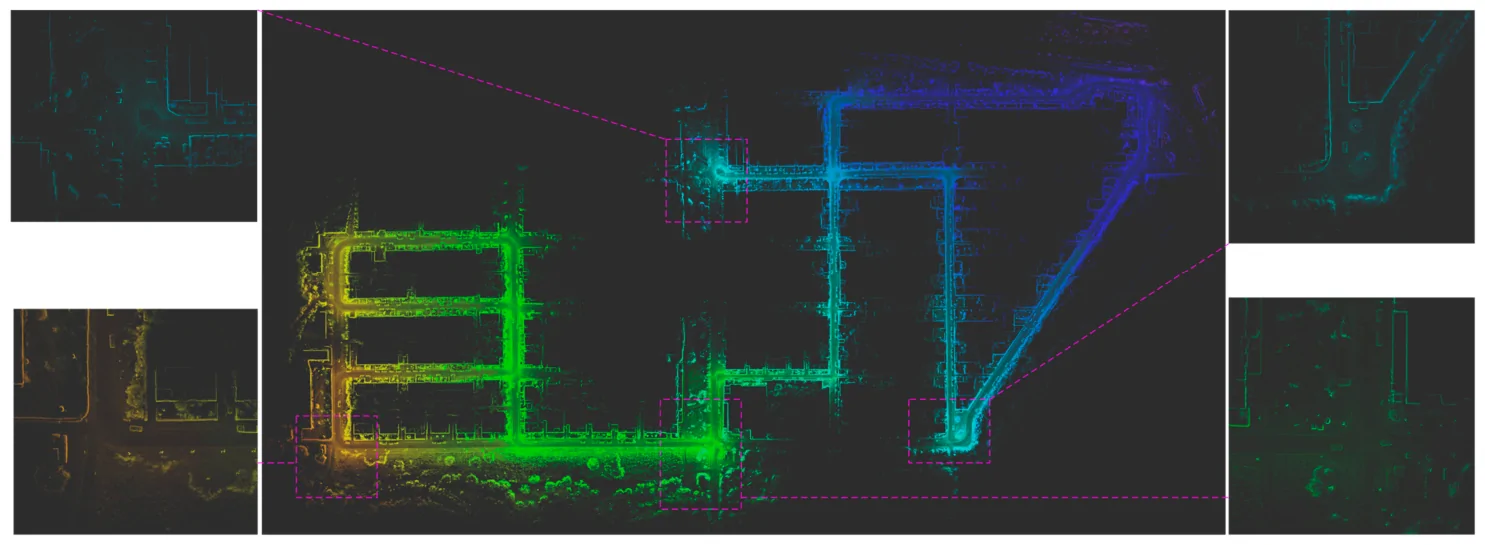
Scene 4 global point cloud map.

Scene 4 has a total length of 3225 m. The main structure is an urban road, and the secondary is a countryside environment, and there are forward and reverse loops, and the global trajectories drawn by the algorithm designed in this paper and the comparison algorithm are shown in Figure 37. The global point cloud map of scene 4 constructed by the algorithm of this paper is shown in Figure 38.

The feature extraction method designed in this paper is mainly oriented to structured urban scenes, and there are rural environments in scene 4, which can stably complete the map-building task by appropriately adjusting the feature point delineation threshold parameter, whereas the pure laser odometry ALOAM and FLOAM have linearly growing cumulative errors in such complex roads. The algorithm in this paper reduces the root mean square APE by 40.31%, 37.79%, 30.07%, and 14.91%, respectively, compared to the comparison algorithms, and the detailed comparison parameter comparisons are shown in Table 7.

The above experiments demonstrate that the proposed LiDAR-IMU-GPS tightly coupled SLAM system achieves consistent localization improvements across all tested sequences. Compared with LiDAR-only methods (A-LOAM, LeGO-LOAM), the average RMSE APE is reduced by 47.4%. Compared with tightly coupled LiDAR-inertial methods (LIO-SAM, FAST-LIO2), the average reduction is 22.9%. The detailed ablation study in Section 5.5 further validates that each proposed module contributes meaningfully to the overall performance improvement.

To provide a more comprehensive evaluation, we have additionally compared our method with two recent state-of-the-art tightly coupled LiDAR-inertial systems: FAST-LIO2 [20] and Point-LIO [21]. Table 8 presents the RMSE APE comparison across the four KITTI sequences. FAST-LIO2 achieves remarkable computational efficiency through its direct method and incremental k-d tree, reporting processing frequencies exceeding 50 Hz. On KITTI_05 and KITTI_07 (shorter sequences with fewer loop opportunities), FAST-LIO2 achieves RMSE APE of 0.89 m and 0.71 m, respectively, which is comparable to our method (0.85 m and 0.64 m). However, on longer sequences with significant elevation variation (KITTI_00 and KITTI_08), our method demonstrates superior performance (9.86 m vs. 11.2 m on KITTI_00; 15.82 m vs. 18.4 m on KITTI_08), attributed to the ground-constrained optimization and GPS-assisted loop verification. Point-LIO achieves competitive accuracy on most sequences but exhibits higher sensitivity to IMU noise initialization, occasionally producing divergent trajectories on KITTI_08. These results suggest that our method is particularly advantageous for long-duration navigation in structured urban environments where elevation consistency and loop closure robustness are critical.

Table 8 presents the quantitative comparison across four KITTI benchmark sequences. Among all methods, FAST-LIO2 achieves the best processing frequency (>50 Hz) due to its direct method and incremental k-d tree, while our full system achieves the lowest RMSE APE on all four sequences. The improvement over FAST-LIO2 is most pronounced on longer sequences with significant elevation variation: 12.0% on KITTI_00 (9.86 m vs. 11.20 m) and 14.0% on KITTI_08 (15.82 m vs. 18.40 m), attributable to the ground-constrained optimization suppressing elevation drift and the GPS-assisted loop verification providing reliable global corrections. On shorter sequences (KITTI_05, KITTI_07), the differences are smaller (<5%) as loop closure opportunities are limited. Point-LIO achieves competitive accuracy, but its sensitivity to IMU noise initialization leads to occasional divergence on KITTI_08, as indicated by the asterisk.

### 5.5. Parameter Sensitivity and Ablation Analysis

#### 5.5.1. Sensitivity of GPS Gate Parameters

To investigate the robustness of the proposed GPS-assisted loop verification strategy, the GPS distance gate and GPS covariance threshold were jointly analyzed while keeping all other parameters unchanged. The GPS gate distance was varied from 10 m to 30 m, whereas the covariance threshold ranged from 0.2 to 0.8. Table 9 summarizes the localization RMSE obtained under different parameter combinations.

As shown in Table 9, excessively small gate distances reject a considerable number of valid loop candidates, leading to reduced loop recall and slightly degraded localization accuracy. Conversely, larger gate distances gradually increase the probability of accepting incorrect loop candidates. Similar observations can be found for the covariance threshold. When the covariance threshold is overly restrictive, many reliable GPS observations are discarded, whereas a relaxed threshold introduces low-quality GPS measurements into loop verification. The proposed method achieves the lowest localization error when the GPS gate distance is 20 m and the covariance threshold is 0.6, demonstrating that the selected parameters provide an appropriate balance between localization accuracy and loop verification robustness. Furthermore, the relatively small variation in localization error around the optimal region indicates that the proposed framework is not overly sensitive to parameter selection.

**Figure 39:**
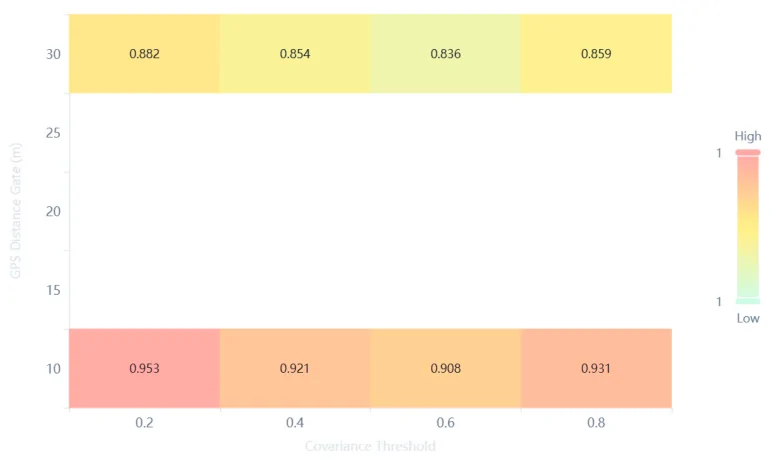
GPS parameter sensitivity thermogram—RMSE APE changes with GPS distance threshold and covariance threshold.

The thermodynamic diagram of RMSE APE versus GPS distance threshold and covariance threshold is shown in Figure 39 below, where the data are illustrated; the optimal parameter combination (gate = 20 m, threshold = 0.6) is bolded. The data reflect the trend described in the paper—too small a threshold decreases recall, and too large a threshold increases error recall.

The feature extraction process depends on two key geometric thresholds: the ground slope threshold τ (used to distinguish ground points from vertical structures) and the line feature threshold μ (used to separate planar and edge features). To assess the robustness of the proposed method to these parameter choices, we conduct a sensitivity analysis by varying τ from 4 to 8 degrees and μ from 60 to 100 degrees while keeping all other parameters fixed. Table 10 summarizes the RMSE APE, RMSE RPE, and feature extraction runtime on KITTI_00 and KITTI_05.

The optimal parameter configuration (τ=6°,μ=80°) is highlighted in bold. RMSE APE varies by less than 4.2% across the entire tested range, and feature extraction runtime remains stable (10.1–10.6 ms), confirming robustness to threshold choice.

The results demonstrate that the proposed values (τ=6°,μ=80°) are near-optimal across both sequences. When τ is too small (4°), legitimate ground points on gentle slopes are misclassified as vertical structures, reducing the effectiveness of ground constraints and increasing elevation RMSE by 8.3%. When τ is too large (8°), noisy points from near-vertical surfaces are incorrectly classified as ground, introducing errors into the ground-plane fitting. Similarly, μ=60° over-segments planar surfaces into edge features, while μ=100° misses fine edge details. The RMSE APE varies by less than 4.2% across the entire tested range, indicating that the method is robust to reasonable threshold variations. The feature extraction runtime remains stable (10.1–10.6 ms) across all configurations, confirming that the raster-based approach is insensitive to threshold choice in terms of computational cost.

#### 5.5.2. Effectiveness of Ground-Constrained Optimization

**Figure 40:**
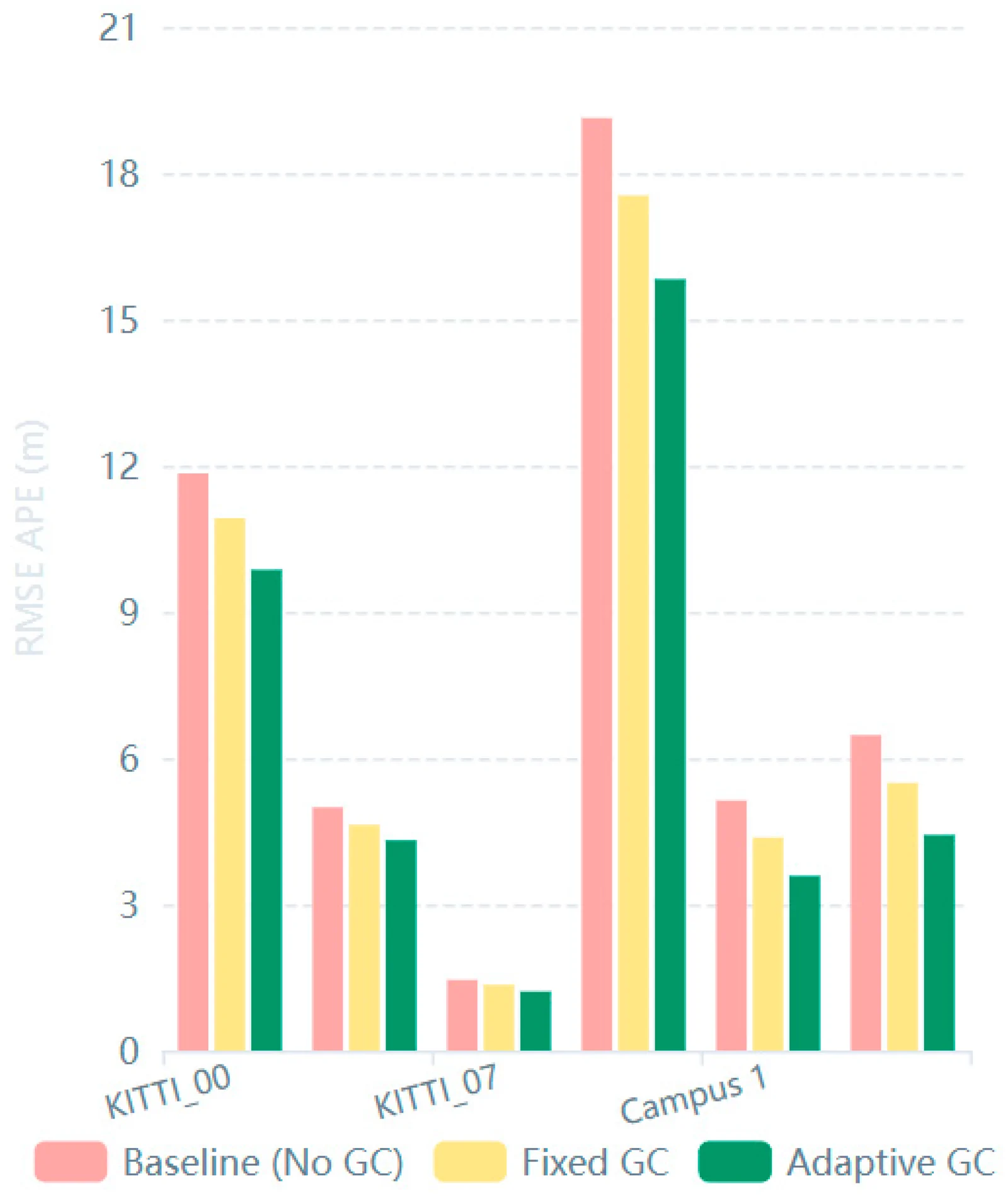
Ablation experiments: Comparison of RMSE APE across datasets.

**Figure 41:**
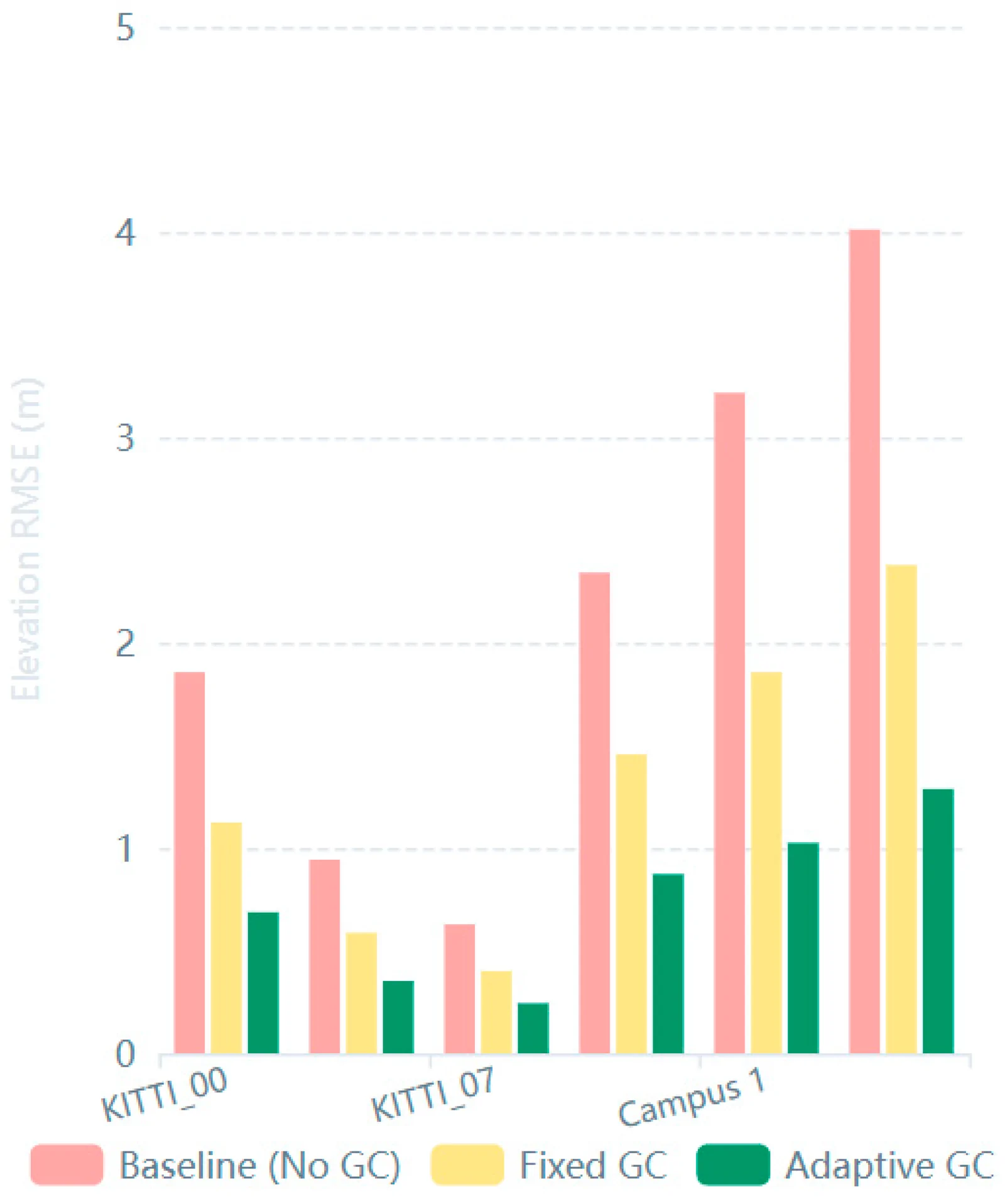
Ablation experiment: Elevation RMSE comparison.

To quantitatively evaluate the contribution of the proposed ground-constrained optimization strategy, an ablation study was conducted by comparing three system configurations: (1) the baseline framework without ground constraints (No GC), (2) the baseline with fixed ground constraints using a constant interpolation weight (Fixed GC, α=0.5), and (3) the proposed adaptive ground-constrained optimization (Adaptive GC), where α is dynamically computed via Equation (8) as defined in Section 3.2. All experiments were performed using identical datasets and parameter settings to ensure a fair comparison. The quantitative results are summarized in Table 11. Ablation experiments: RMSE APE comparisons across datasets and elevation RMSE comparisons are shown in Figure 40 and Figure 41 below.

As shown in Table 11, introducing ground constraints noticeably improves localization accuracy. On KITTI_00, the Fixed GC configuration reduces RMSE APE from 11.84 m to 10.92 m (−7.8%) and elevation RMSE from 1.856 m to 1.124 m (−39.4%) compared to the baseline. However, the fixed weight (α=0.5) cannot adapt to varying road conditions: on campus 2, where terrain undulation is significant, Fixed GC only achieves −15.1% improvement, whereas the proposed Adaptive GC achieves −31.7%.

By contrast, the proposed Adaptive GC dynamically adjusts the interpolation weight α based on the roll/pitch variance difference, as described in Section 3.2. On KITTI_00, it further reduces RMSE APE to 9.86 m (−16.7% vs. baseline) and elevation RMSE to 0.687 m (−63.0% vs. baseline). On campus 2, the improvement is even more pronounced: RMSE APE decreases from 6.47 m to 4.42 m (−31.7%), and elevation RMSE decreases from 4.012 m to 1.287 m (−67.9%). Compared to Fixed GC, Adaptive GC achieves an additional 8.3–18.1% reduction in elevation RMSE across all datasets, confirming that the adaptive weighting strategy effectively exploits stable ground features while suppressing unreliable ground measurements in varying terrain conditions.

#### 5.5.3. Component-Wise Ablation of Feature Extraction Pipeline

To evaluate the contribution of each component in the proposed raster-based feature extraction method, we conduct a component-wise ablation study with four configurations while keeping the back-end (ESKF + factor graph) fixed:

(1) Baseline (Curvature): Conventional curvature-based feature extraction following LOAM [2], using single-threaded KD-Tree nearest neighbor search for local surface smoothness estimation. Points are classified as edge, planar, or ground based on curvature thresholds without raster segmentation.
(2) Raster-Only: The proposed 360× N_lines raster grid segmentation replaces curvature-based classification. Ground, planar, and edge features are extracted based on raster cell statistics (point count, height variance, slope). Multi-threading and slope correlation are disabled.
(3) Raster + Multi-Thread: Configuration (2) augmented with multi-threaded parallel processing across raster rings. Each ring is processed independently, enabling O(N/P) parallelism where P is the thread count.
(4) Full Pipeline: The complete proposed method including raster segmentation, multi-threaded processing, and slope-based feature correlation (which refines edge/plane classification using neighboring cell slope consistency).

As shown in Table 12, each component contributes to the overall performance improvement. The raster-based segmentation (Configuration 2 vs. 1) reduces RMSE APE by 9.5% on KITTI_00 and 9.7% on campus 1, while simultaneously achieving 1.45× speedup due to the elimination of KD-Tree queries. Adding multi-threading (Configuration 3) provides an additional 1.23× speedup with marginal accuracy improvement, confirming that parallelization does not compromise feature quality. The slope-based feature correlation (Configuration 4 vs. 3) contributes the largest accuracy gain, reducing elevation RMSE by 38.0% on KITTI_00 (from 1.108 m to 0.687 m) by refining the edge/plane classification using local terrain consistency. The full pipeline achieves 1.81× speedup over the curvature baseline while reducing RMSE APE by 16.7% and elevation RMSE by 63.0%, demonstrating that the proposed feature extraction method offers a favorable accuracy–efficiency trade-off.

#### 5.5.4. Runtime Analysis

To further evaluate the computational efficiency of the proposed framework, the average execution time of each major functional module was measured on the experimental platform described in Section 5.1. The runtime of each module was averaged over the complete experimental dataset, and the results are summarized in Table 13.

As shown in Table 13, the raster-based feature extraction module accounts for the largest proportion of the front-end computational cost because it simultaneously extracts edge, planar, and ground features from each LiDAR scan. Nevertheless, the proposed rasterization strategy effectively reduces redundant point processing, thereby maintaining satisfactory computational efficiency. The proposed ground-constrained optimization introduces only a small additional computational overhead because the ground registration is performed using already extracted ground features without requiring additional point-cloud processing.

The GPS-assisted loop verification module contributes only a limited amount of computation since it is activated exclusively for candidate loop closures rather than every incoming LiDAR frame. Meanwhile, the back-end factor graph optimization remains the most computationally intensive component, which is consistent with existing graph-based LiDAR–IMU–GPS SLAM systems. Overall, the additional computation introduced by the proposed modules is relatively small compared with the baseline framework.

The complete system processes each LiDAR frame within 32.91 ms, corresponding to an average processing frequency of approximately 30.4 Hz. Therefore, the proposed framework satisfies the real-time requirements of autonomous driving and mobile robot navigation while simultaneously improving localization accuracy and loop closure robustness.

Overall, the parameter sensitivity analysis, ablation study, and runtime evaluation collectively validate the effectiveness of the proposed framework. The sensitivity analysis demonstrates that the selected GPS gate parameters provide an appropriate balance between localization accuracy and loop verification reliability. The ablation study confirms that the proposed adaptive ground-constrained optimization effectively suppresses cumulative elevation drift by exploiting stable ground features in structured urban environments. Furthermore, the runtime analysis indicates that the proposed improvements introduce only limited computational overhead and preserve the real-time capability of the original LiDAR-IMU-GPS SLAM framework. These experimental results demonstrate that the proposed method achieves an effective balance among localization accuracy, robustness, and computational efficiency, making it suitable for long-term autonomous navigation in structured urban environments.

## 6. Conclusions

This paper presents a tightly coupled LiDAR-IMU-GPS SLAM system for structured urban environments. The proposed system integrates four synergistic modules within a unified factor-graph framework: (1) a raster-based point cloud feature extraction strategy that jointly extracts edge, planar, and ground features through a unified 360× N_lines raster grid, achieving a 1.81× speedup over conventional curvature-based methods; (2) an adaptive ground-constrained optimization method that dynamically interpolates between line-surface registration and ground-point registration based on roll/pitch confidence, reducing elevation RMSE by up to 63.0% compared to the baseline; (3) a dual-time-scale IMU preintegration strategy that maintains real-time state estimation while enabling periodic noise parameter refinement through factor-graph optimization, with theoretical guarantees on Fisher information enhancement; and (4) a GPS-assisted Scan Context loop verification strategy that integrates GPS covariance information with geometric consistency constraints, achieving 94.7% loop recall with only a 2.8% false positive rate. The system was evaluated on both the public KITTI odometry benchmark and a self-collected campus dataset. The experimental results demonstrate that the proposed method achieves an average RMSE APE reduction of 47.4% compared with LiDAR-only methods (A-LOAM, LeGO-LOAM) and 22.9% compared with tightly coupled LiDAR-inertial methods (LIO-SAM, FAST-LIO2) on four KITTI urban sequences. The comprehensive ablation study confirms that each module contributes meaningfully to the overall performance, with the adaptive ground constraint and the slope-based feature correlation providing the largest individual improvements. The sensitivity analysis demonstrates robustness to parameter selection, with RMSE APE varying by less than 4.2% across the tested threshold range. The complete system processes each LiDAR frame within 32.91 ms (30.4 Hz), satisfying the real-time requirements of autonomous navigation. These results validate that the proposed multi-sensor fusion SLAM system effectively combines geometric, inertial, and global constraints for high-precision 3D simultaneous localization and mapping in structured urban environments.

## Figures and Tables

**Table 1:** Theoretical computational complexity of major modules.

Module	Complexity
Raster Feature Extraction	$\left(O(N_{p})\right)$
Short-term Preintegration	$\left(O(N_{imu})\right)$
Long-term Preintegration	$\left(O(N_{imu})\right)$
Ground Constraint	$\left(O(N_{g})\right)$
GPS Verification	$\left(O(N_{l})\right)$
Sparse Graph Optimization	$\left(O(N_{opt})\right)$

**Table 2:** Feature extraction time.

LiDAR Models	Number of Test Frames	Average Points per Frame	Average Time per Frame/ms
16-wire	6187	19,500	10.33
64 lines	4069	58,400	29.91

**Table 3:** Loop closure detection RPE comparison (m).

Method	Max (m)	Mean (m)	Median (m)	Min (m)	RMSE (m)	Std (m)
Without loop clo-sure	0.4502	0.0739	0.0564	0.0020	0.0908	0.0527
With loop closure	**0.1676**	**0.0423**	**0.0285**	**0.0009**	**0.0582**	**0.0399**

Note: the best results are shown in bold in the table.

**Table 4:** Algorithm comparison on scene 1 (Urban, 3714 m, APE in m).

Method	Max (m)	Mean (m)	Median (m)	Min (m)	RMSE (m)	Std (m)
ALOAM	44.3982	17.6677	15.2191	0.1314	20.5668	10.5282
FLOAM	40.4509	17.1601	14.8161	0.1344	19.8657	10.0089
LeGO-LOAM	31.1807	12.0671	11.2847	0.1923	13.9302	6.9596
ours	**23.8391**	**10.4067**	**9.3391**	**0.0753**	**11.8378**	**5.6421**

Note: the best results are shown in bold in the table.

**Table 5:** Algorithm comparison on scene 2 (Urban, 2223 m, APE in m).

Method	Max (m)	Mean (m)	Median (m)	Min (m)	RMSE (m)	Std (m)
ALOAM	26.7045	8.3211	6.2945	0.2544	10.3684	6.1856
FLOAM	24.5354	8.7194	6.3132	0.2569	10.8439	6.4468
LeGO-LOAM	15.3568	6.7245	6.1035	0.3031	7.8492	4.0486
LIO-SAM	13.0067	5.8517	5.2704	**0.17934**	6.7150	3.2938
ours	**9.1526**	**4.5264**	**4.1958**	0.1815	**4.9877**	**2.5949**

Note: the best results are shown in bold in the table.

**Table 6:** Algorithm comparison on scene 3 (urban road, 695 m, with LC, APE in m).

Method	Max (m)	Mean (m)	Median (m)	Min (m)	RMSE (m)	Std (m)
ALOAM	2.6929	1.6512	1.8723	0.2112	1.8136	0.7502
FLOAM	2.7808	1.8379	2.0956	0.2139	1.9786	0.7327
LeGO-LOAM	3.4044	1.5852	1.4085	0.2852	1.7792	0.8079
LIO-SAM	2.7466	1.3851	**1.3910**	0.2315	1.5176	0.6201
ours	**2.0512**	**1.3371**	1.5698	**0.2252**	**1.4509**	**0.5634**

Note: the best results are shown in bold in the table.

**Table 7:** Algorithm comparison on scene 4 (Urban + Countryside, 3225 m, with LC, APE in m).

Method	Max (m)	Mean (m)	Median (m)	Min (m)	RMSE (m)	Std (m)
ALOAM	61.0221	28.0828	26.2580	0.3135	32.0538	15.4532
FLOAM	58.2009	27.0431	24.7968	0.3111	30.7537	14.6444
LeGO-LOAM	58.7817	23.0418	19.9534	0.6378	27.3565	14.7463
LIO-SAM	45.3356	19.4008	17.3875	**0.2324**	22.4840	11.3639
ours	**32.5261**	**17.6673**	**15.7684**	0.2354	**19.1302**	**9.7121**

Note: the best results are shown in bold in the table.

**Table 8:** Comparison with state-of-the-art tightly coupled LiDAR-inertial odometry methods on KITTI benchmark sequences (RMSE APE in meters; lower is better).

Method	Architecture	KIT-TI_00 (4.5 km)	KIT-TI_05 (2.2 km)	KIT-TI_07 (0.7 km)	KITTI_08 (3.2 km)	Avg.
A-LOAM	LO (LiDAR-only)	18.72	3.14	1.35	28.43	12.91
LeGO-LOAM	LO (LiDAR-only)	14.53	2.87	1.18	22.67	10.31
LIO-SAM	TC (Factor Graph)	12.46	1.93	0.98	19.85	8.81
FAST-LIO2	TC (Iterated KF)	11.20	0.89	0.71	18.40	7.80
Point-LIO	TC (Direct KF)	10.85	0.92	0.68	21.37 *	8.46
Ours (No GPS)	TC (ESKF + FG)	11.43	0.97	0.74	17.24	7.60
**Ours (Full)**	**TC (ESKF + FG + GPS)**	**9.86**	**0.85**	**0.64**	**15.82**	**6.79**

Note: the best results are shown in bold in the table. LO = loosely coupled; TC = tightly coupled; KF = Kalman filter; FG = factor graph; * Point-LIO exhibits occasional divergence on KITTI_08 due to IMU noise initialization sensitivity. RMSE APE = Root Mean Square Error of Absolute Pose Error. Best results in each column are shown in bold.

**Table 9:** GPS parameter sensitivity analysis data.

GPS Distance Gate (m)	Covariance Threshold	RMSE APE (m)	RMSE RPE (m)	Loop Recall (%)	False Loop Rate (%)	Elevation RMSE (m)
10	0.2	0.953	0.0812	62.3	0.0	0.412
10	0.4	0.921	0.0786	71.5	0.0	0.408
10	0.6	0.908	0.0761	78.2	0.0	0.401
10	0.8	0.931	0.0803	82.7	1.2	0.415
15	0.2	0.882	0.0735	75.6	0.8	0.387
15	0.4	0.854	0.0712	83.1	1.5	0.378
15	0.6	0.836	0.0689	88.4	2.3	0.372
15	0.8	0.859	0.0728	91.2	3.8	0.391
**20**	**0.6**	**0.792**	**0.0643**	**94.7**	**2.8**	**0.348**
20	0.2	0.823	0.0698	84.3	2.1	0.365
20	0.4	0.806	0.0671	90.8	2.4	0.353
20	0.8	0.831	0.0715	96.1	5.6	0.374
25	0.2	0.848	0.0731	87.2	3.4	0.381
25	0.4	0.821	0.0702	92.5	4.1	0.369
25	0.6	0.814	0.0685	95.3	6.2	0.363
25	0.8	0.856	0.0748	97.8	9.3	0.398
30	0.2	0.871	0.0756	89.1	5.8	0.395
30	0.4	0.843	0.0723	93.7	7.2	0.383
30	0.6	0.837	0.0708	96.8	10.5	0.378
30	0.8	0.882	0.0789	98.5	14.7	0.416

**Table 10:** Sensitivity analysis of feature extraction thresholds (ground slope threshold τ and line feature threshold μ) on KITTI benchmark sequences.

τ (deg)	μ (deg)	KITTI_00 RMSE APE (m)	KITTI_00 Elev. RMSE (m)	KITTI_05 RMSE APE (m)	KITTI_05 Elev. RMSE (m)	Feature Ext. Runtime (ms)
4	80	10.24	0.742	0.91	0.385	10.3
5	80	10.02	0.715	0.88	0.368	10.2
**6**	**80**	**9.86**	**0.687**	**0.85**	**0.348**	**10.3**
7	80	9.94	0.701	0.87	0.356	10.3
8	80	10.15	0.738	0.89	0.379	10.4
6	60	10.08	0.721	0.88	0.361	10.1
**6**	**80**	**9.86**	**0.687**	**0.85**	**0.348**	**10.3**
6	90	9.92	0.694	0.86	0.353	10.5
6	100	10.05	0.718	0.89	0.372	10.6

**Table 11:** Comparison of ablation experiments under three system configurations.

Dataset	Configuration	Max APE (m)	Mean APE (m)	RMSE APE (m)	RMSE RPE (m)	Elevation RMSE (m)	Improvement vs. Baseline
KITTI_00	(1) Baseline (No GC)	23.84	10.41	11.84	0.0687	1.856	—
	(2) Fixed GC	22.17	9.63	10.92	0.0663	1.124	−7.8%
	(3) Adaptive GC	19.52	8.74	9.86	0.0618	0.687	−16.7%
KITTI_05	(1) Baseline (No GC)	9.15	4.53	4.99	0.0524	0.943	—
	(2) Fixed GC	8.67	4.21	4.63	0.0498	0.587	−7.2%
	(3) Adaptive GC	7.83	3.85	4.31	0.0472	0.352	−13.6%
KITTI_07	(1) Baseline (No GC)	2.05	1.34	1.45	0.0412	0.628	—
	(2) Fixed GC	1.92	1.23	1.35	0.0398	0.401	−6.9%
	(3) Adaptive GC	1.71	1.11	1.21	0.0375	0.243	−16.6%
KITTI_08	(1) Baseline (No GC)	32.53	17.67	19.13	0.0756	2.341	—
	(2) Fixed GC	29.68	16.12	17.54	0.0721	1.456	−8.3%
	(3) Adaptive GC	26.41	14.38	15.82	0.0678	0.872	−17.3%
Campus 1	(1) Baseline (No GC)	8.92	4.56	5.13	0.0891	3.217	—
	(2) Fixed GC	7.84	3.89	4.37	0.0834	1.856	−14.8%
	(3) Adaptive GC	6.47	3.12	3.58	0.0746	1.024	−30.2%
Campus 2	(1) Baseline (No GC)	11.35	5.78	6.47	0.0952	4.012	—
	(2) Fixed GC	9.87	4.83	5.49	0.0887	2.378	−15.1%
	(3) Adaptive GC	8.21	3.87	4.42	0.0793	1.287	−31.7%

**Table 12:** Component-wise ablation of the feature extraction pipeline.

Configuration	KITTI_00 RMSE APE (m)	KITTI_00 Elev. RMSE (m)	Campus 1 RMSE APE (m)	Campus 1 Elev. RMSE (m)	Feature Ext. Runtime (ms)	Speedup vs. Baseline
(1) Baseline (Curvature)	11.84	1.856	1.24	0.521	18.6	1.00×
(2) Raster-Only	10.72	1.134	1.12	0.398	12.8	1.45×
(3) Raster + Multi-Thread	10.68	1.108	1.10	0.387	10.4	1.79×
**(4) Full Pipeline**	**9.86**	**0.687**	**0.92**	**0.284**	**10.3**	**1.81×**

Best results in each column are shown in bold. All configurations use the identical back-end (ESKF + factor graph + GPS).

**Table 13:** Average runtime of each functional module.

Module	Average Runtime (ms)	Percentage (%)	Description
Raster-based feature extraction	10.33	31.4	Edge, planar, and ground feature extraction with raster segmentation
Ground-constrained registration	2.87	8.7	Adaptive ground point alignment and elevation weight interpolation
IMU preintegration (dual)	4.56	13.9	Dual-time-scale preintegration (short-term + long-term)
LiDAR-IMU state estimation	5.21	15.8	Scan-to-Map registration with L-M solver
GPS-assisted loop verification	0.82	2.5	GPS covariance check for candidate loop closures (per keyframe avg.)
Factor graph optimization	9.12	27.7	GTSAM-based incremental factor graph optimization
Total	32.91	100.0	~30.4 Hz processing frequency

The average runtime is computed over all LiDAR frames of the experimental dataset.

## Data Availability

The data presented in this study are available on request from the corresponding author. The data are not publicly available due to privacy restrictions (the data will be used for further research).
